# Volume-transmitted GABA waves pace epileptiform rhythms in the hippocampal network

**DOI:** 10.1016/j.cub.2023.02.051

**Published:** 2023-04-10

**Authors:** Vincent Magloire, Leonid P. Savtchenko, Thomas P. Jensen, Sergyi Sylantyev, Olga Kopach, Nicholas Cole, Olga Tyurikova, Dimitri M. Kullmann, Matthew C. Walker, Jonathan S. Marvin, Loren L. Looger, Jeremy P. Hasseman, Ilya Kolb, Ivan Pavlov, Dmitri A. Rusakov

**Affiliations:** 1 UCL Queen Square Institute of Neurology, University College London, Queen Square, London WC1N 3BG, UK; 2 Rowett Institute, University of Aberdeen, Ashgrove Road West, Aberdeen AB25 2ZD, UK; 3 Janelia Research Campus, Howard Hughes Medical Institute, Ashburn, VA, USA; 4 Howard Hughes Medical Institute, University of California, San Diego, La Jolla, CA 92093, USA; 5 GENIE Project Team, Janelia Research Campus, Howard Hughes Medical Institute, Ashburn, VA, USA

**Keywords:** epilepsy, brain rhythms, tonic GABA conductance, extracellular GABA, iGABASnFR2, GAT-1, GABA uptake, spiking neural networks, volume transmission

## Abstract

Mechanisms that entrain and pace rhythmic epileptiform discharges remain debated. Traditionally, the quest to understand them has focused on interneuronal networks driven by synaptic GABAergic connections. However, synchronized interneuronal discharges could also trigger the transient elevations of extracellular GABA across the tissue volume, thus raising tonic conductance (*G*_tonic_) of synaptic and extrasynaptic GABA receptors in multiple cells. Here, we monitor extracellular GABA in hippocampal slices using patch-clamp GABA “sniffer” and a novel optical GABA sensor, showing that periodic epileptiform discharges are preceded by transient, region-wide waves of extracellular GABA. Neural network simulations that incorporate volume-transmitted GABA signals point to a cycle of GABA-driven network inhibition and disinhibition underpinning this relationship. We test and validate this hypothesis using simultaneous patch-clamp recordings from multiple neurons and selective optogenetic stimulation of fast-spiking interneurons. Critically, reducing GABA uptake in order to decelerate extracellular GABA fluctuations—without affecting synaptic GABAergic transmission or resting GABA levels—slows down rhythmic activity. Our findings thus unveil a key role of extrasynaptic, volume-transmitted GABA in pacing regenerative rhythmic activity in brain networks.

## Highlights

• GABA sniffer and optical sensor report [GABA]_e_ rises before interictal events
• Interneuronal network models relate rhythmicity to volume-transmitted GABA actions
• Multi-cell recordings support the concept of periodical GABA-driven disinhibition
• GABA transporters tune the rhythms of interictal events

## Teaser

Magloire et al. show that periodic epileptiform discharges are preceded by transient waves of extracellular GABA, which appear critical for the timing of such brain network rhythms.

## Introduction

GABA is the principal inhibitory neurotransmitter in the brain. It mediates conventional fast inhibitory synaptic transmission through ionotropic Cl^−^-permeable GABA_A_ receptors (GABA_A_Rs), as well as slower transmission mediated by GABA_B_ receptors. However, in certain conditions, such as postsynaptic cell depolarization or high intracellular chloride concentration ([Cl^−^]), GABA_A_R current can become excitatory.^1^ In addition, there is a slow GABA_A_R-mediated conductance (*G*_tonic_), often referred to as “tonic inhibition,” which reflects recurrent receptor activation by GABA present throughout the extracellular space. Historically, microdialysis studies *in vivo* have suggested that the ambient extracellular GABA concentration ([GABA]_e_) is relatively stable, although it may change with physiological state.^2^ Indeed, long-lasting changes in average [GABA]_e_ should be constrained by GABA transporters.^3^^,^^4^ However, the dialysis technique usually reports [GABA]_e_ averaged over many seconds, whereas the affinity of the main GABA transporter GAT-1 (*K*_m_ = 10–20 μM)^5^^,^^6^^,^^7^ is too low to prevent faster, low-micromolar-range fluctuations in [GABA]_e_.

The dynamics of *G*_tonic_ have been associated with the firing of interneurons^8^ and are implicated in epileptiform discharge initiation.^4^^,^^9^ Changes in *G*_tonic_ can also alter the excitability of principal cells (PCs),^10^^,^^11^^,^^12^^,^^13^ influencing their network behavior,^14^^,^^15^ including basic rules of coincidence detection.^16^^,^^17^^,^^18^ While a collapse in inhibitory activity has been associated with epileptiform events,^19^ interneurons appear to maintain or increase their firing rate before the onset of ictal activity,^20^^,^^21^ thus implying a role in the generation of ictal events.^22^^,^^23^ In addition, seizure-like events in pyramidal neurons can be preceded by fast-spiking (FS) neuronal firing,^24^ leading to an outward GABA_A_R current and a rise in extracellular K^+^.^23^ Optogenetic activation of GABAergic cells can paradoxically engage both ictal and interictal discharges in both *ex vivo* and *in vivo* acute models of epilepsy^25^^,^^26^^,^^27^^,^^28^^,^^29^ (reviewed in Magloire et al.^30^). However, when monitored in bulk, the majority of interneurons do not increase their activity during spontaneous seizures *in vivo*,^31^ reflecting the heterogeneous nature of their involvement.^32^^,^^33^^,^^34^ While these observations implicate complex dynamic interactions between network inhibition and excitation in the generation of interictal and ictal discharges, how these interactions regulate epileptiform rhythms remains poorly understood.

Intriguingly, we have previously found a bell-shaped relationship between *G*_tonic_ (controlled through either dynamic clamp or by changing ambient [GABA]_e_), and the firing and synchronization of interneuronal networks.^12^^,^^15^ Because *G*_tonic_ itself depends on interneuronal firing, this type of relationship suggests, at least in theory, an inherent capacity for self-organizing rhythms or periodic waves of activity.^35^ Genetically encoded optical GABA sensors have reported prominent [GABA]_e_ transients (10- to 100-ms timescale) during interictal spikes *in vivo*,^36^ arguing that fluctuations in *G*_tonic_ may have a role in periodic network activity in the intact brain.

We therefore hypothesized that the synchronized firing of hippocampal interneurons should drive transient, volume-transmitted changes in [GABA]_e_, which could in turn control *G*_tonic_ and thus regulate rhythmic network activity such as interictal events (IIEs). We tested this hypothesis by combining outside-out membrane patch (GABA sniffer) recordings,^37^^,^^38^ the novel optical GABA sensor iGABASnFR2,^39^ an improved version of iGABASnFR,^36^ and optogenetic stimulation of FS, parvalbumin-positive (PV+) interneurons, with computer simulations of spiking neural networks.^40^^,^^41^ We also asked whether reducing GABA uptake, which should curb extracellular GABA waves but not synaptic GABAergic transmission or ambient [GABA]_e_, would alter the rhythm of IIEs.

## Results

### Transient [GABA]_e_ rises tend to precede IIEs

**Figure 1:**
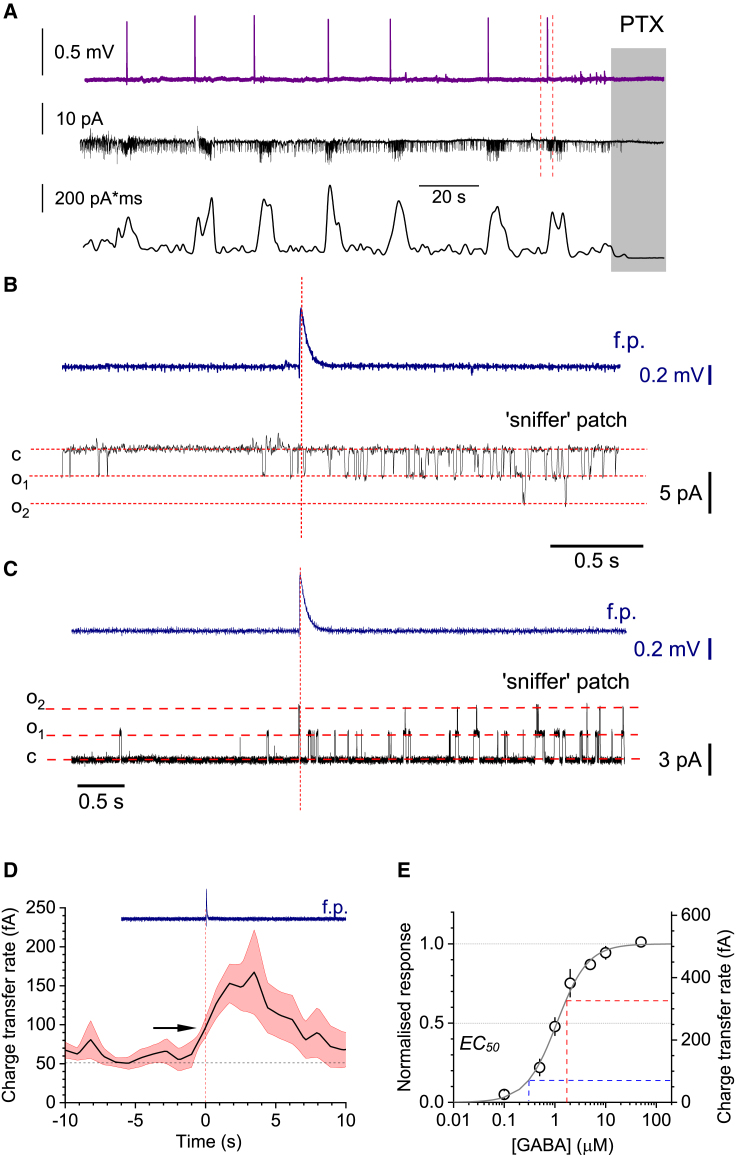
Rhythmic fluctuations of extracellular GABA during interictal activity in the hippocampal area CA1 (A) Local field potentials (LFPs, top trace), sniffer-patch single-channel activity (middle), and charge dynamics (bottom) during interictal discharges in hippocampal slices. Gray shade, picrotoxin (PTX) application at the end of trials. See also Figure S1A. (B) Fragment from (A) (between dotted lines), expanded to show the LFP (top, blue) and patch recording (bottom, black) details. High-chloride pipette solution was used to record inward GABA_A_R-mediated channel openings (V_m_ = −70 mV). (C) Similar setting to (B), but with low chloride solution to document outward currents (V_m_ = 0 mV). (D) Averaged time course of charge transfer rate (equal to current; mean ± SEM for slice-average traces, n = 6 slices) at the onset of individual field potential (f.p.) IIE (vertical dashed line, blue trace). Arrow, interictal discharge onset coincides with GABA_A_R activity reaching ∼35% of its peak. (E) Normalized and absolute charge transfer values recorded in sniffer patches at different [GABA]_e_ (dots, mean ± SEM; n = 4–6 for individual values; gray line, sigmoid best fit). Dashed lines, [GABA]_e_ during resting level (blue) and peak (red) of channel activity recorded. See Figures S1B–S1G for field-potential and single-cell recordings.

First, we elicited epileptiform activity in acute hippocampal slices perfused with a 0 [Mg^2+^]/5 mM [K^+^] solution; in the CA1 region, this activity generated brief (<200 ms) periodic field-potential spikes reflecting IIEs^42^^,^^43^ (Figure 1A, top trace). To monitor the [GABA]_e_ dynamics in these settings, we used an outside-out “sniffer” patch, which reports GABA_A_R channel activity,^37^ as previously described.^18^^,^^38^ Channel opening frequency appeared synchronized with IIEs (Figure 1A, middle), yet a detailed analysis revealed that it always started to increase 0.5–1 s before IIEs (Figures 1B, 1C, and S1A; summary in Figure 1D). We took special care to prepare uniformly shaped sniffer patch pipettes for these experiments, and used a multi-channel rapid solution exchange to calibrate [GABA]_e_ against GABA_A_R channel activity.^44^ We thus estimated an EC_50_ value of ∼1.1 μM, which corresponds to a charge transfer rate (or current) of 256 ± 57 fC·s^−1^ (fA; mean ± SEM here and below unless shown otherwise; n = 6; Figure 1E; note that the average of individual peak values differs from the peak value of the average charge transfer trace centered at the IIE onset). Thus, we estimated that [GABA]_e_ rises from its resting level of ∼0.3 μM to a peak of 1.5–2 μM during IIEs (Figure 1E).

We next explored the cellular basis of these observations. The spiking activity of individual FS PV+ interneurons increased substantially from their basal level several seconds before the peak of CA1 pyramidal cell bursts that represent IIEs (Figures S1B and S1C). This was faithfully reflected by the elevated GABAergic input to CA1 pyramidal cells before the IIEs (Figures S1D and S1E). In contrast, pyramidal cell spiking appeared time-locked to the epileptiform discharges (Figures S1F and S1G). These observations reflect earlier findings that interictal bursting is preceded by an increase in firing of FS PV+ interneurons,^19^^,^^23^^,^^29^^,^^45^ which we suggest represents, at least partly, a “global” wave of [GABA]_e_ detected with the sniffer patch (Figure 1).

### Interictal spikes are preceded by a [GABA]_e_ elevation landscape across tissue

**Figure 2:**
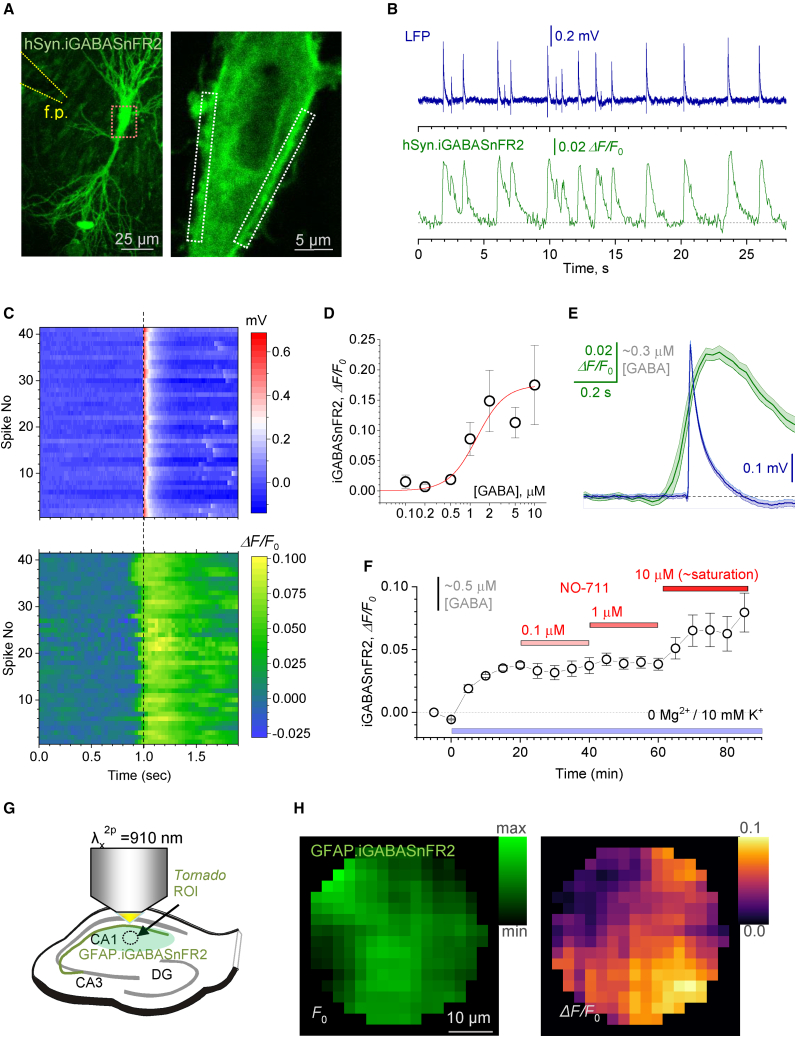
Optical registration of [GABA]_e_ dynamics using iGABASnFR2 expressed in hippocampal slices (A) CA1 pyramidal cell expressing iGABASnFR2 with a field pipette electrode (f.p., left) and two imaging ROIs near the cell soma periphery (right, zoomed-in fragment shown by red rectangle on the left; λ_x_^2p^ = 910 nm). (B) One-slice example, fEPSPs (top) and iGABASnFR2 signal (bottom) recorded during interictal spikes. Dotted line, apparent baseline reflecting the resting/equilibrated [GABA]_e_. See Figure S2A for further detail. (C) Diagram showing 41 interictal spikes aligned with respect to their peak times (top), and the corresponding iGABASnFR2 signals (bottom; false color scale). See Figure S2B for details of field potential recordings. (D) iGABASnFR2 fluorescence increment against increasing [GABA]_e_ in acute hippocampal slice preparation (mean ± SEM; n = 7 slices). Line, best-fit sigmoidal Hill function at *E* = 0.175; *K*_*d*_ = 1.26 μM, n = 2 (STAR Methods). (E) Summary of LFP and iGABASnFR2 recordings shown in (B) and (C), aligned in time, as indicated: traces and shaded area, mean ± 95% confidence interval (n = 41). (F) Average iGABASnFR2 signal (mean ± SEM, n = 3 hippocampal slices, 5–10 ROIs each) monitored in slices in control conditions, under epileptiform conditions (10 mM [K^+^]_out_), and under partial GAT-1 transporter blockade achieved by using sub-saturation concentrations of the blocker NO-711, as indicated. Approximate scale bar for [GABA] in (E) and (F) is derived from data in (D). (G) Experimental diagram: imaging IIE-driven GABA release landscape in CA1 neuropil with high spatiotemporal resolution using astroglia-expressed iGABASnFR2. See Figure S2C for further illustration. (H) Fluorescence landscape of iGABASnFR2 (15 spiral-scans/30 ms average) in baseline conditions (before an IIE; left) and the Δ*F*/*F*_0_ GABA signal landscape immediately after an IIE (right). Average for four consecutive IIEs.

Although the sniffer patch can detect [GABA]_e_ changes with high sensitivity, it reports average [GABA]_e_ near the slice surface, potentially far away from the GABA release sites—and at any rate, averaged over many cells and environments. To understand whether [GABA]_e_ displays similar dynamics within the pyramidal cell layer (a key target area of FS interneurons), deep within the tissue, we used the recently developed optical GABA sensor iGABASnFR2,^39^ a novel version of iGABASnFR.^36^ First, we expressed the sensor in a proportion of CA1 and CA3 pyramidal cells under the *hSynapsin-1* promoter, in organotypic hippocampal slices (Figure 2A): sparse expression of iGABASnFR2 should reduce the overall extracellular presence of GABA-binding sites and therefore minimize any potential buffering effects on the [GABA]_e_ dynamics, as demonstrated earlier for glutamate and its sensors.^46^^,^^47^

We imaged iGABASnFR2 fluorescence within microscopic regions of interest (ROIs) near the periphery of individual pyramidal cell somata using two-photon excitation microscopy, as described earlier,^48^^,^^49^ while recording field potentials nearby (Figure 2A). Periodic IIEs were consistently accompanied by transient elevations of iGABASnFR2 fluorescence that, again, started to rise before individual IIEs (Figures 2B, 2C, and S2A): the [GABA]_e_ rise onset was consistently 80–90 ms prior to IIE onset. Although the inter-spike interval in this preparation (2–4 s) was shorter than that in acute slices (15–20 s; Figure 1A), the recovery of [GABA]_e_ to its resting (equilibrated) level took a similar fraction of the periodic cycle in both preparations. In the context of network dynamics (see below), this correspondence is consistent with the role of the GABA wave time course in pacing the network rhythm.

To translate our imaging data into (approximate) [GABA]_e_, we recorded iGABASnFR2 fluorescence against increasing [GABA] applied externally, in isolated acute hippocampal slice preparations. The resulting dependence (Figure 2D) suggested that, during IIEs, the peak iGABASnFR2 signal of 0.074 ± 0.006 Δ*F*/*F*_0_ (mean ± 95% CI) corresponds to an ∼1.1-μM increase in [GABA]_e_ (Figure 2E). This estimate is in excellent agreement with the sniffer patch data (Figure 1E).

In such experiments, high [K^+^]_out_/low [Mg^2+^] (“epileptogenic artificial cerebrospinal fluid [aCSF]”) boosts cell excitability, prompting generation of IIEs. To understand whether this manipulation also increases ambient [GABA]_e_, we monitored iGABASnFR2 fluorescence in normal aCSF, after introducing epileptogenic aCSF, and under the partial blockade of the main GABA transporter GAT-1, using increasing concentrations of its blocker NO-711 (Figure 2F). The results suggest that introducing an epileptogenic solution raises [GABA]_e_ by 0.7–0.8 μM, and that the partial blockade of GAT-1 does not change this new equilibrium until the blockade reaches a saturation level (Figure 2F). We also asked whether fluctuations in [K^+^]_out_ could be involved in pacing IIEs, but our electrophysiological recordings argued against it, in present conditions, by indicating no K^+^-driven depolarizing shifts before IIEs (Figure S2B).

Finally, we asked whether [GABA]_e_ elevations were homogeneous across the neuropil. To address this, we used acute slices with iGABASnFR2 expressed in astroglia (Figure 2G), which provided nearly contiguous territories of expression (Figure S2C), so that we could image GABA release landscapes during IIEs. These experiments also require high temporal resolution, such as millisecond-range *Tornado*-scanning, demonstrated before,^48^ to avoid rapid spatial equilibration of the diffusing GABA signal. A characteristic snapshot of the iGABASnFR2 signal during an IIE (30 ms Δ*F*/*F*_0_ window) illustrates the heterogeneous landscape of GABA release (Figure 2H), which should reflect uneven distribution of active GABAergic axons.

### Biophysical basis of [GABA]_e_-dependent network rhythms

**Figure 3:**
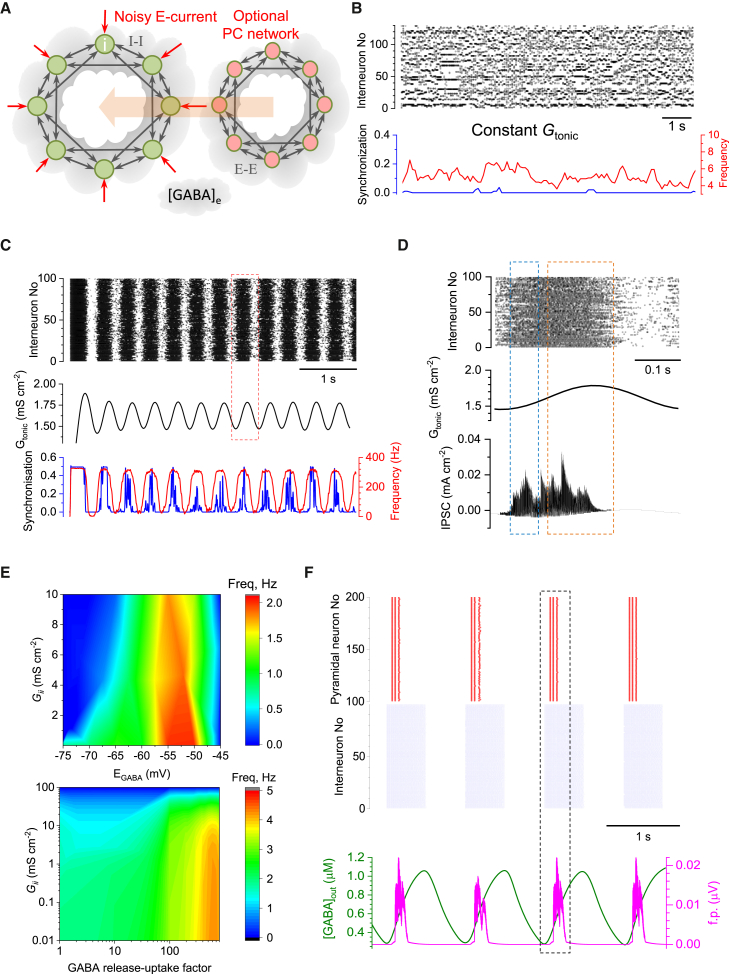
[GABA]_e_-dependent *G*_tonic_ steers rhythmic activity of a modeled interneuron network across a range of parameters (A) Schematic. FS interneuron network model (*I-I*, GABAergic synaptic connections), with external excitatory inputs (*E*-current); volume-transmitted [GABA]_e_ signal generating *G*_tonic_; and an optional network of pyramidal cells incorporated (*E-E*, excitatory connections). (B) Raster plot of interneuronal network spiking (top), network synchronization coefficient (bottom, blue), and time course of average spiking frequency (bottom, red) for simulated network with constant *G*_tonic_ value (0.44 mS cm^−2^). (C) Raster plot as in (B), but with *G*_tonic_ driven by [GABA]_e_ (middle) calculated from integrated interneuronal discharges; network synchrony and mean network frequency show clear periodicity (bottom). Key model parameters: cell number N = 100, intra-network peak synaptic conductance *G*_*ii*_ = 0.1 mS cm^−2^; *E*-currents (Poisson series) with average synaptic conductance *g*_s_ = 0.02 mS cm^−2^, decay constant tau = 3 ms, and frequency *f*_*s*_ = 100 Hz; GABA_A_R reversal potential *V*_*GABA*_ = −56 mV, GABA release factor *A*_*f*_ = 1.35 × 10^−7^ nS cm^−2^ ms^−1^, *G*_pump_ = 0.003 ms^−1^ (STAR Methods). (D) Fragment from (C) (red dotted rectangle) enlarged, with the integrated IPSC time course (bottom). Blue and orange dotted rectangles indicate high-frequency, non-synchronized, and oscillating and synchronized IPSC periods, respectively. (E) Parameter-space heatmaps: rhythm generation over a range of *G*_*ii*_ and *E*_*GABA*_ (*R* = 1, top), and *G*_*ii*_ and release-uptake factor *R* (STAR Methods; *E*_*GABA*_ = −65 mV, bottom). Deep blue area indicates no detectable rhythmic activity. (F) Top: raster plot for a twinned network (red, pyramidal neurons; blue, interneurons), with weak internetwork (*I-E* and *E-I*) and strong intra-network (*I-I* and *E-E*) connections. Bottom: time course of field potential (f.p.; simulated for pyramidal neurons at 250 μm from the network, tissue conductance of 100 mS cm^−1^; STAR Methods) and [GABA]_e_, as indicated. Key model parameters: N = 200 (100 pyramidal cells and 100 interneurons), *G*_*ii*_ = 0.216 mS cm^−2^, *G*_ee_ = 0.003 mS cm^−2^; internetwork peak synaptic conductance *G*_ei_ = 0.00012 mS cm^−2^ and *G*_ie_ = 0.00064 mS cm^−2^, *E*-currents (Poisson series) *g*_si_ = 0.3 mS cm^−2^, tau = 3 ms, *f*_*s*_ = 20 Hz; *I*-current (Poisson series) *g*_se_ = 0.003 mS cm^−2^, decay constant tau = 3 ms, *f*_*s*_ = 20 Hz, *V*_*GABA*_ = −58 mV, *A*_*f*_ = 2 × 10^−7^ nS cm^−2^ ms^−1^, *G*_pump_ = 0.003 ms^−1^. See Figures S3 and S4 for further exploration of the model parameter space.

We next explored the mechanistic relationship between [GABA]_e_ fluctuations and IIE rhythms, by expanding our earlier models^15^^,^^40^^,^^41^ to simulate a network of FS hippocampal interneurons equipped with stochastic excitatory inputs (Figure 3A). While previous modeling studies have proposed a plausible network machinery of high-frequency rhythms, including sharp-wave ripples,^50^^,^^51^^,^^52^ they did not consider mechanisms relevant to IIE generation: 0.2- to 1.0-Hz frequency range or the potential role of GABA waves. One theoretical study neatly suggested extracellular K^+^ waves as the volume-transmitted feedback driving 2- to 5-Hz network oscillations,^53^ but such [K^+^]_out_ fluctuations could also regulate glutamate and GABA uptake. Here, we postulated that interneuronal discharges generated both IPSCs and volume-transmitted [GABA]_e_ rises (Figure 3A) that contributed to *G*_tonic_ in multiple cells.^40^^,^^41^ *G*_tonic_ was thus set as a monotonic function of [GABA]_e_, which in turn was determined by (1) a scalable [GABA]_e_ increment upon each individual synaptic discharge and (2) GABA uptake by GAT-1 providing an exponential [GABA]_e_ decay^6^^,^^54^ (STAR Methods).

We simulated networks of varied sizes, over a plausible range of synaptic GABA release intensities and GABA uptake rates. It was clear that under constant [GABA]_e_ (hence *G*_tonic_), cells would fire stochastically at a near-constant average frequency with little synchronization (Figure 3B). In striking contrast, when [GABA]_e_ is driven by interneuronal spiking, the network slips into periodic activity (Figures 3C and 3D) over a wide range of frequencies, depending on the excitatory drive (*E*-current), synaptic connectivity strength (*G*_*ii*_), and the GABA-release-uptake scaling factor *R* (Figure 3E; STAR Methods). There was a robust relationship between interneuronal spiking, synchronization parameter *k* (STAR Methods), [GABA]_e_, and *G*_tonic_ during periodic bursts of activity (Figures 3C–3E and S3). Interestingly, larger networks (containing 400–1,500 cells) tended to have a more regular activity cycle (Figure S4), consistent with classical theory predicting less variability near a system’s Hopf bifurcation point,^55^ which occurs toward the peak of *G*_tonic_.

Finally, we explored the relationship between cell spiking activity, *G*_tonic_, and [GABA]_e_ in a twinned network configuration incorporating both interneurons and PCs equipped with GABA_A_Rs (Figure 3A). First, such networks turned out to show stable behavior under external stimuli when connectivity between PCs and interneurons was ∼100 times weaker than PC-PC or interneuron-interneuron connectivity. We next found that the PC network activity was highly sensitive to *G*_tonic_ generated by the interneuronal network, over a wide range of stochastic excitatory input. PCs showed brief, highly synchronized bursts of activity that followed periodic rises of interneuronal spiking (Figure 3F). Strikingly, the PC spiking bursts occurred on the ascending phase of [GABA]_e_ waves (Figure 3F), thus displaying some distinct features of the epileptiform events that we observed experimentally (Figures 1D, 2B, 2C, and 2E).

### Self-maintained waves of tonic GABA_A_ conductance in hippocampal slices

To test whether the relationships suggested by the network models were valid, we carried out several experiments. First, we asked whether the dynamics of *G*_tonic_ predicted by simulations (Figures 3A–3D) occur in hippocampal circuits in the absence of glutamatergic transmission. We therefore recorded GABA_A_R-mediated whole-cell currents from CA1 pyramidal neurons in the presence of AMPA, NMDA, and GABA_B_ receptor blockers.

**Figure 4:**
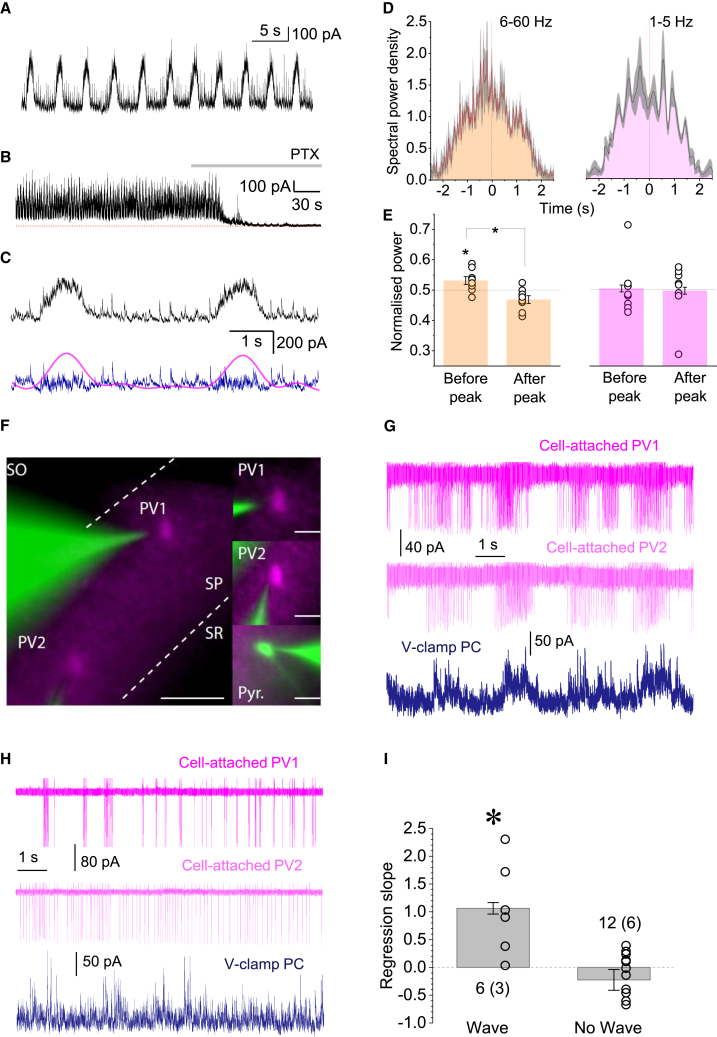
Slow wave-like oscillations and synchronization of FS PV+ interneuron spiking activity during GABA waves (A) Trace. Example of GABA_A_R-mediated currents received by a CA1 pyramidal neuron (voltage-clamp with low intracellular Cl^−^; V_h_ = 0 mV). (B) Slow, wave-like oscillations as in (A) are readily blocked by the GABA_A_R antagonist picrotoxin (PTX). (C) Traces as in (A) expanded: raw data (black), and its 2 Hz low-pass (*G*_tonic_; magenta) and high-pass (blue) filtered components. (D) Example, average spectrogram plots (mean ± 95 CI; high and low frequency bands, as indicated); data over multiple IIEs in one slice. (E) Summary of the analysis shown in (D), for n = 7 slices: bar graphs, relative spectral power (bars, mean ± SEM; dots, individual experiments), before and after the *G*_tonic_ peak, as indicated. ^∗^p < 0.05, one-sample (left) and paired-sample (middle) Student’s t test. (F) Illustration of dual cell-attached recordings of FS PV+ interneurons (green, 50 μM Alexa Fluor 488; magenta, tdTomato) and voltage-clamp recording of a CA1 pyramidal cell (50 μM AF488). Scale bars, 50 μm (left) and 20 μm (right). (G) Example time course of spiking activity of two simultaneously recorded FS PV+ cells (top and middle traces), and IPSCs in a pyramidal neuron (bottom, V_hold_ = +10 mV) in high-K^+^ solution (10 mM) and 0 Mg^2+^. Rhythmic network waves are present. (H) Experiment as in (G) but with no detectable rhythmic waves; notations as in (G). (I) Summary. Regression slope (mean ± SEM) between PV+ spiking intensity and synchronization parameter in dual-patch recordings. Samples with rhythmic waves (n = 6 cells in 3 slices) and without (n = 12 cells in 6 slices) are shown; ^∗^p ∼ 0.038, Kruskal-Wallis non-parametric ANOVA (Z = 2.065; dependent datasets within 3 and 6 slices).

Elevating [K^+^]_out_ to 10 mM (in 0 Mg^2+^) elicited an outward shift in the holding current, which was sensitive to the non-competitive GABA_A_R channel blocker picrotoxin (PTX) (Figures 4A and 4B) and consisted of brief transients riding on periodic low-frequency waves (Figure 4C). The frequency of slow GABA_A_R-mediated waves (0.2–0.3 Hz) was similar to the periodic [GABA]_e_ transients observed during GABA imaging experiments (Figure 2B). Consistent with the network model (Figures 3A–3D, S3, and S4), fast GABA_A_R-mediated currents, which most likely reflect individual interneuron discharges, tended to increase in intensity prior to the peak of the slow *G*_tonic_ transients reflecting [GABA]_e_ waves (Figures 4C–4E).

### Synchronization of FS PV+ interneurons during GABA waves

To understand the dynamics of synchronized interneuronal activity during GABA waves, we carried out triple-cell recordings monitoring pairs of FS PV+ interneurons (cell-attached) and a CA1 pyramidal cell (whole cell), with ionotropic glutamate receptors blocked (Figure 4F). Clear rhythmicity of interneuron activity was documented in three out of nine experiments: wave-like activity (STAR Methods) occurred in both cell-attached FS PV+ interneurons (Figure 4G), suggesting that ∼35% of FS PV+ interneurons could steer epileptiform discharges. This appeared in good correspondence with network simulations predicting that peak synchronization involves up to 40% of interneurons (Figure 3C). Indeed, over 50% of interneurons in areas CA1/CA3 show no change in firing during spontaneous IIEs *in vivo*.^31^

Here, quasi-periodic bursts of interneuron spiking were observed, together with the corresponding fluctuations of GABAergic currents in the recorded pyramidal neuron (Figure 4G). Overall, experiments that featured rhythmic waves (Figure 4G) also showed a significant correlation between spiking frequency and synchronization of the two PV+ cells, whereas recordings with little rhythmicity (Figure 4H) corresponded to no such correlation, reflecting possibly intense yet non-synchronized firing (Figure 4I). In these experiments, the wave pattern was detected in one out of three cases, although our network simulations suggest its prevalence (Figure 3). This apparent discrepancy is likely to arise because modeled cells have equivalent intrinsic and connectivity properties, whereas real cells and circuits are highly diverse. The latter may explain the reported heterogeneity of interneuronal involvement in IIE generation.^32^^,^^33^^,^^34^ Indeed, we detected heterogeneous GABA landscapes during IIEs (Figure 2H), arguing that the detection of coordinated rhythms in 1/3 of cases involving randomly selected cells is consistent with our hypothesis.

### Spiking of principal neurons is synchronized shortly after the peak of interneuronal firing

**Figure 5:**
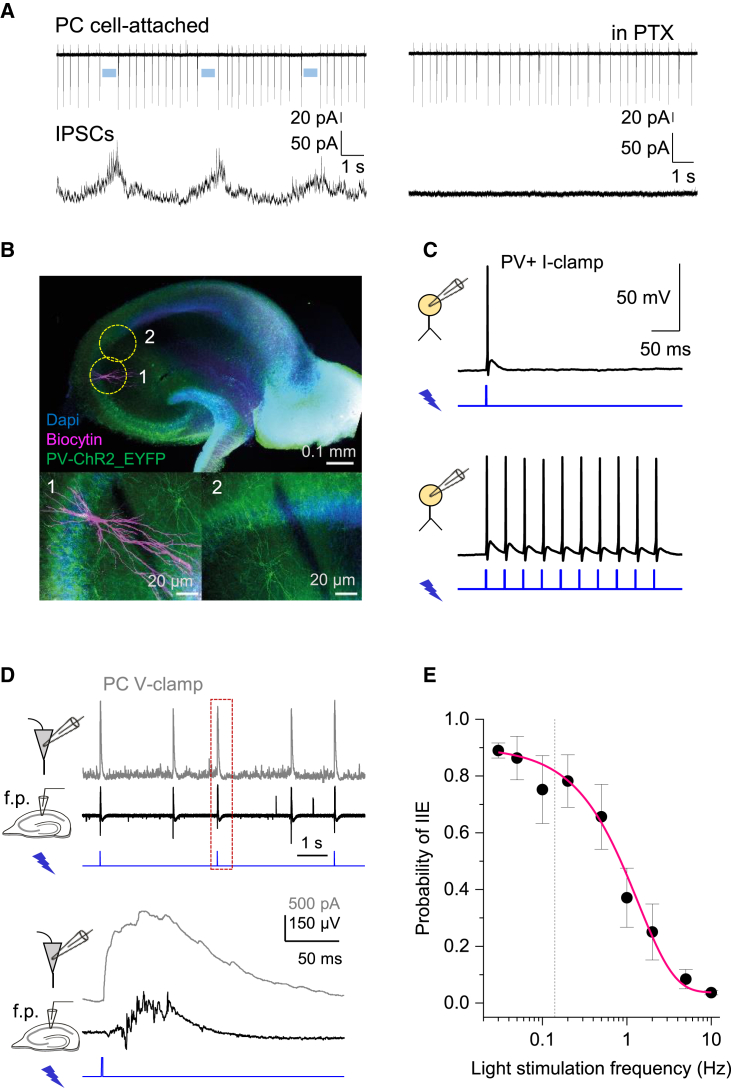
Short photoactivation of FS PV+ interneurons evokes epileptiform burst discharges (A) Simultaneous cell-attached and whole-cell recordings of action potentials (top trace) and GABA_A_R-mediated IPSCs (bottom) during [GABA]_e_ waves (left) and after application of picrotoxin (PTX). Blue segments indicate pauses in PC spiking before the peaks of IPSCs. See also Figure S5A. (B) Transverse hippocampal slice of PV::*Cre* × Ai32 mouse (top): green fluorescence shows ChR2 expression in PV+ cells against DAPI (blue) nuclear counterstain (STAR Methods). Images 1–2 (areas indicated by dotted circles above): post hoc identification of a CA3 pyramidal neuron (biocytin-filled, magenta; 1) after a whole-cell recording shown below in (D). (C) Current-clamp recordings from a FS PV+ interneuron activated by a single (left) or repetitive (right) blue light pulses (1 ms, 470 nm). (D) Example of pyramidal neuron IPSCs (gray trace, top; V_hold_ = +10 mV) and field potentials (black trace, middle): photo-stimulation of FS PV+ cells (blue trace, bottom; 1-ms pulse) triggers large GABA_A_R currents and epileptiform bursts similar to those occurring spontaneously (e.g., Figure 1). 5 mM K^+^, 0 Mg^2+^ solution. See Figures S5B–S5D for further detail. (E) Statistical summary: probability of evoking an interictal event (IIE, mean ± SEM, n = 3–7 slices for individual values) as a function of light stimulation frequency. The occurrence of failures increases with stimulation frequency. The average frequency of spontaneous IIE (0.14 ± 0.03 Hz, n = 7 slices, dotted line) falls within the range of optimal frequencies to entrain the network with light stimuli. Above 0.2 Hz stimulation, the network enters into a refractory state. See Figure S6A for an example.

Next, to relate interneuronal activity to PC firing, we recorded two neighboring CA1 or CA3 pyramidal cells, one in cell-attached and one in voltage-clamp mode (10 mM [K^+^]_out_ ionotropic glutamate receptors intact). Pyramidal cell spiking showed a characteristic lull during the rise of *G*_tonic_ transients (Figure 5A, left, blue segments), yet resumed spiking near their peak. This “network rebound” phenomenon has been explored previously,^12^^,^^15^^,^^56^ suggesting that the shunting (self-inhibition) of interneuronal networks prompts relatively rapid, network-driven disinhibition of PCs near the peak of interneuronal activity. Blocking GABA_A_Rs with PTX abolished this periodic fluctuation (Figure 5A right).

Thus, a rise in interneuron activity could paradoxically initiate IIEs by synchronizing PC firing, the phenomenon explored previously.^23^ To test this further, we expressed channelrhodopsin-2 (ChR2-H134R-eYFP) in the hippocampal FS PV+ cells (Figure 5B) to enable their synchronous activation using widefield optogenetic stimulation (Figure 5C). We asked whether such stimulation could prompt IIEs in epileptogenic aCSF (5 mM [K^+^]_out_, 0 [Mg^2+^]). Indeed, a single 1-ms light pulse evoked large GABA_A_R IPSCs in CA1 pyramidal neurons, generating coincident epileptiform field potentials that were indistinguishable from spontaneous IIEs (Figures 5D and S5A). Again, this observation was similar to that reported in cortical slices under a different epileptogenic protocol.^29^

Blocking GABA_A_R with PTX abolished light-evoked IIEs, but not other periodic bursts (Figures 5D and S5A). This revealed the two different types of short epileptiform discharges observed in slices, pre-ictal bursts that are purely glutamatergic, and IIEs that involve both glutamatergic and GABAergic activity.^57^^,^^58^ Optogenetic stimulation of FS PV+ cells evoked IIEs and spike bursts in pyramidal neurons, with comparable post-stimulus lags (31 ± 6 ms for IIEs and 46 ± 4 ms for pyramidal spikes; Figures S5B–S5D). Thus, an abrupt release of FS PV+ cell inhibition upon light turnoff triggered synchronous PC discharges. Overall, light stimuli applied at 0.03–0.2 Hz evoked IIEs with ∼0.8 probability, whereas stimulation at > 0.2 Hz (value close to natural periodicity, 0.14 ± 0.03 Hz) was less effective, with the probability decreasing to ∼0.1 at 5–10 Hz (Figures 5E and S6A).

### Evoked [GABA]_e_ waves can entrain network rhythms

**Figure 6:**
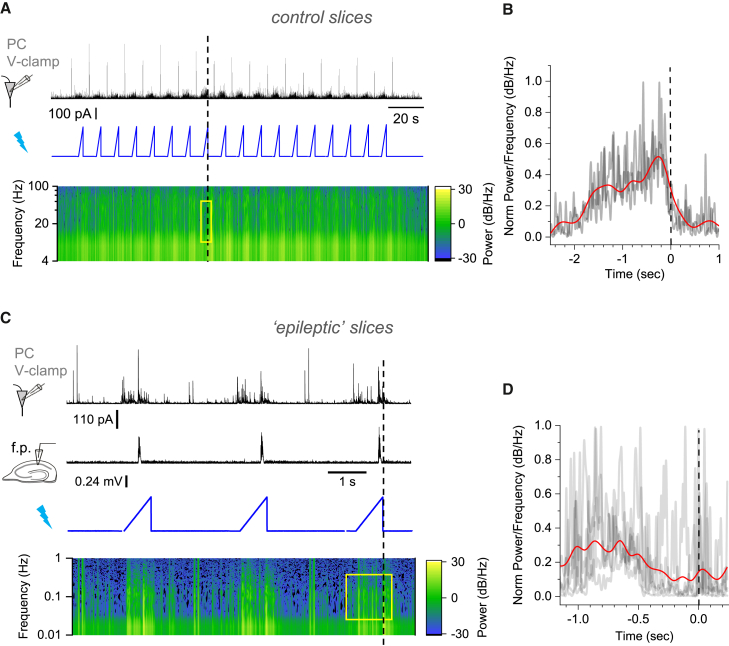
Optogenetically evoked GABA waves entrain epileptiform burst discharges (A) GABA_A_R current in pyramidal neurons (V_hold_ = +10 mV, normal aCSF solution; black trace, top) in response to progressive opto-activation of FS PV+ interneurons (blue, middle), with the IPSC power spectrum density (bottom). Dotted line, end of individual light ramps. (B) Average spectral power density (5–50 Hz interval, n = 18 ramp stimulations). Time window as exemplified by yellow rectangle in (A) near light intensity peaks (dotted line). (C) Tests in epileptogenic tissue (0 Mg^2+^ and 5 mM K^+^ aCSF): GABA_A_R current in pyramidal neurons (black trace, top) and local field potentials (middle) during opto-activation (blue trace), with power spectrum density (bottom). Note that interictal bursts occur toward the end of the stimulation ramp (vertical dotted line). See Figure S6B for further recording detail and Figures S7A and S7B for enforced rhythm entraining reproduced by the neural network model. (D) Average of spectral power density for time window exemplified by yellow rectangle in (C); notations as in (B).

To see whether a relatively slow wave of GABA release could entrain IIEs, we light-activated PV+ cells using longer-lasting ramp stimuli (1- to 2-s duration, comparable to spontaneous GABA waves), while recording IPSCs in CA1 pyramidal cells. In normal aCSF, this stimulation elevated GABAergic activity only to a certain point, after which the IPSC frequency rapidly dropped (Figures 6A and 6B), whereas in epileptogenic aCSF it led to IIEs (Figures 6C, 6D, and S6B). Indeed, in five out of eight experiments, we observed wave-like GABA_A_R-mediated currents induced by optogenetic ramp stimulation, inducing IIEs with a probability of 0.25 ± 0.09 and a delay of 831 ± 47 ms (detection window between 500 and 100 ms post-stimulation; Figure S6C). In contrast, in the three experiments without light-evoked GABA waves, the probability of IIE generation was only 0.06 ± 0.04.

To understand the biophysical basis of this phenomenon, we asked whether, in our simulated network, the *G*_tonic_ dynamics on their own could pace network synchronization and rhythmicity. We therefore forced *G*_tonic_ to follow a 1-Hz sine wave and found that cell firing indeed peaked at *G*_tonic_ troughs, which were in turn preceded by maximal firing synchronicity (Figures S7A and S7B), in line with the experimental data. Thus, increased interneuronal activity initially boosts network inhibition, after which disinhibition (probably shunting^12^^,^^15^) prevails, at which point synchronized network discharges are likely to occur, as generally predicted by our theoretical model. Throughout these recordings, we did not detect depolarization transients that might arise from significant [K^+^]_out_ elevations due to ChR2 activation,^59^ probably because the conditions of high potassium and brief optogenetic stimuli in our case were outside the range for such effects.

### GABA uptake controls the frequency of periodic epileptiform events

**Figure 7:**
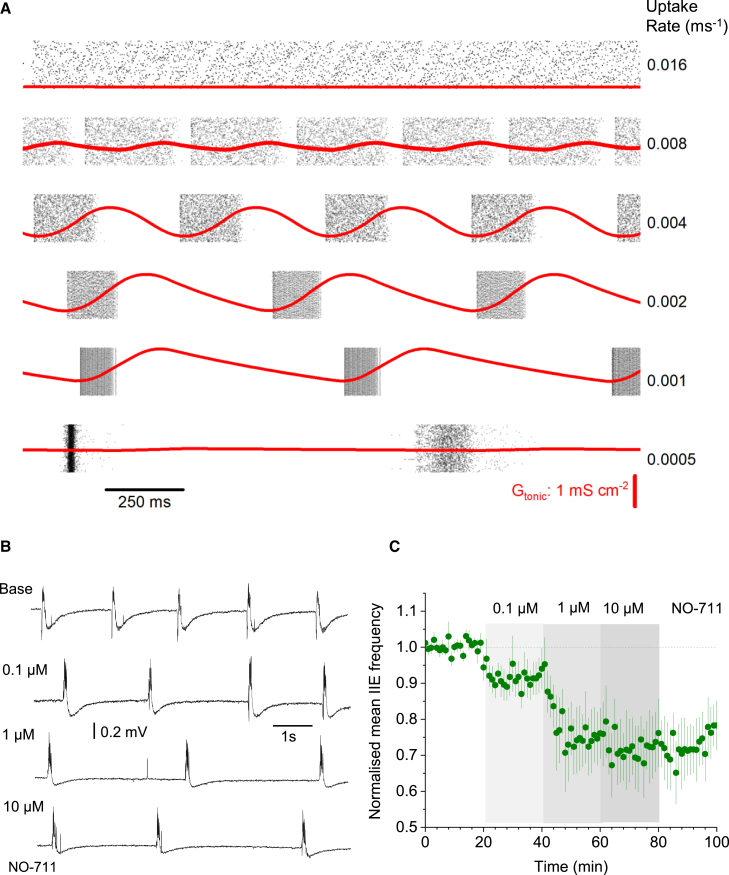
GABA uptake rate controls rhythmic activity of interneuronal networks (A) Raster plots of interneuron spiking (dots; for presentation clarity, 6,000 randomly selected spike events shown only) and time course of *G*_tonic_ at different GABA uptake rates (red lines), as indicated. Key model parameters: cell number N = 1,500, intra-network peak synaptic conductance *G*_*ii*_ = 0.096 mS cm^−2^; *E*-currents (Poisson series) with average synaptic conductance *g*_s_ = 0.05 nS, decay constant tau = 3 ms, and frequency *f*_*s*_ = 20 Hz; GABA_A_R reversal potential *V*_*GABA*_ = −53 mV, GABA release factor *Af* = 10^−8^ nS cm^−2^ ms^−1^, *G*_pump_ = 0.004 ms^−1^ (see STAR Methods for further detail). *G*_tonic_ scale (bottom panel) applies throughout. (B) Examples of interictal spiking recorded in slice, under basal conditions (0 Mg^2+^ and 5 mM [K^+^], base), and during subsequent bath applications of the GABA uptake blocker NO-711 at increasing concentrations, as indicated. See Figure S7C for an example containing CA1 pyramidal cell IPSC recording. (C) Average frequency of interictal events (mean ± SEM, n = 6 slices), normalized to the baseline value, in baseline conditions and during NO-711 application, as indicated.

The rate of GABA uptake depends on the numbers of available GABA transporters, dictating how rapidly [GABA]_e_ returns to its equilibrium level. The latter level, however, depends on the transporter kinetics, not on their number (unless this number is negligible), according to the first principles of reaction-kinetic theory and assuming no consistent change in extracellular GABA supply. This was consistent with our iGABASnFR2 imaging data, indicating no changes in [GABA]_e_ under partial blockade of GAT-1 (Figure 2F). Similarly, changes in the uptake rate should have little effect on the rapid activation of intra-synaptic receptors that are exposed to thousands of molecules released at once.^60^ Thus, reducing the number of GABA transporters should decelerate the [GABA]_e_ wave dynamics without changing synaptic GABAergic transmission. To test these principles theoretically, we simulated a 1,500-strong cell network (as in Figure S4) and found that increasing the GABA uptake rate up to a certain level (∼0.016 ms^−1^) could drastically reduce the [GABA]_e_ dynamic range, thus effectively suppressing activity rhythms (Figure 7A, top). Conversely, slowing down GABA uptake allowed [GABA]_e_ to fluctuate rhythmically, with lower decay rates producing slower interneuronal network activities (Figure 7A).

To test whether this causality is indeed present in real neuronal networks, we monitored IIEs in slices, while adding a progressively increasing concentration of NO-711 (as in Figure 2F) up to 10 μM, at which point its effect should saturate.^61^ Reducing the fraction of available GAT-1 should thus slow down [GABA]_e_ reequilibration without affecting phasic GABAergic transmission or the basal [GABA]_e_ level. Strikingly, partial GAT-1 blockade indeed reduced the frequency of IIEs, in exact correspondence with the modeling predictions (Figures 7B and 7C). In some experiments, we also monitored local field potential (LFP) and pyramidal neuron activity simultaneously, which again revealed transients of tonic GABA current prior to field spikes (Figure S7C). These results provide compelling evidence that [GABA]_e_ waves play a key role in the pacing of rhythmic activity in interneuronal networks.

## Discussion

Interneuronal networks, in particular FS PV+ cells, can exhibit rhythmic synchronization at IIE frequencies, with or without fast glutamatergic signaling. Among interneurons, excitation can arise from depolarizing currents mediated by GABA_A_Rs,^1^ depending on receptor reversal potential and the activity-driven dynamics of intracellular chloride and [K^+^]_out_. The underlying machinery involves the key chloride-potassium extruder, KCC2, and possibly other transporter mechanisms,^62^^,^^63^ but their exact nature is outside the scope of the present study.

Here, we sought to understand how fluctuations in *G*_tonic_, which are driven mainly by the activity-dependent dynamics of [GABA]_e_, can entrain slow periodic network activity such as IIEs. The waves of [GABA]_e_ provide a volume-transmitted feedback signal that modulates neuronal firing. Reducing *G*_tonic_ from its steady-state level boosts CA1 pyramidal cell firing, while having little effect on interneuronal activity,^64^ pointing to the tonic disinhibition of PCs. However, increasing *G*_tonic_ above a certain level could have a similar effect on PCs as it shunts interneuronal activity, as shown earlier.^12^^,^^15^ At that point, disinhibition (network rebound excitation) of PCs prevails, leading to synchronized network discharges. However, that earlier work^15^ explored a steady-state case for [GABA]_e_—and, therefore, *G*_tonic_—with no volume-transmitted GABA signals. In that case, (1) low *G*_tonic_ had little effect on synchronization, (2) its initial increase boosted excitation and synchronization, and (3) increasing *G*_tonic_ further reduced excitability due to shunting, thus reducing neuronal synchronization. In contrast, the present study deals with the nonstationary network dynamics engaging [GABA]_e_-dependent feedback, leading to a different parameter space for the firing synchronization phenomena. We find that the pattern of network activity and its predisposition to oscillations must depend on the *G*_tonic_ dynamics driven by GABA release, diffusion, and uptake. However, this does not imply that the GABA wave mechanism described here is the only factor entraining network rhythms. After all, the primary source of GABA waves is synaptic activity of GABAergic interneurons, whereas the target of the waves must involve both synaptic and extrasynaptic GABA_A_Rs.

### Potential limitations of the high potassium model

In the high-K^+^ experimental models, depolarization due to elevated [K^+^]_out_ varies among neurons and their dendrites and thus provides a stochastic excitatory drive to interneurons when excitatory transmission is blocked. By design, therefore, such interneuron-only models still involve some excitatory drive in triggering interictal activity. In addition, increasing [K^+^]_out_ up to 10 mM is likely to elevate internal [Cl^−^] up to 6 mM,^65^ which helps to set GABA_A_R reversal potential within the interneuron shunting range and thus prompt epileptiform activity under intact glutamatergic transmission. Whether similar conditions can occur *in vivo* remains to be ascertained.

While increasing [K^+^]_out_ by adding KCl has been a common practice to induce epileptiform activity, it may raise some basic questions regarding GABA homeostasis. However, the kinetics of GABA transporters per se is independent of [K^+^]_out_ and depends almost linearly on membrane potential.^54^ In addition, increasing [Cl^−^]_out_ by 5–10 mM from the baseline (normally 110–120 mM) only changes the GABA transport reversal potential *V*_*GAT*_ by ∼1%–2%, according to the basic expression for *V*_*GAT*_ (STAR Methods). Finally, evidence from our imaging experiments with iGABASnFR2 indicates that a 10-mM increase in external [KCl] elevates resting extracellular [GABA] up to 0.6–0.7 μM, which remains virtually unchanged under partial blockade of GAT-1 (Figure 2F). Altogether, these observations suggest that using high potassium is unlikely to engage some poorly controlled concomitant mechanisms of GABA transport that would affect mechanistic interpretations of our data.

### [GABA]_e_ dynamic range and activation of extrasynaptic receptors

We find that during IIEs in hippocampal slices [GABA]_e_ peaks at about 1.5–2 μM (Figures 1E and 2E). Calibration of the sniffer-patch sensor in acute slices indicates that [GABA]_e_ is maintained at 0.25–0.5 μM in nominally Mg^2+^-free aCSF (Figure 1E). This [GABA]_e_ range is 2–5 times greater than that in quiescent slices at near-physiological [Mg^2+^] (estimated previously using the same technique^38^), likely because of a higher spontaneous interneuron firing rate. iGABASnFR2 imaging indicates that applying 10 mM [K^+^]_out_/0 [Mg^2+^] elevates [GABA]_e_ from its basal level by 0.7–0.8 μM (Figure 1F). These estimates are generally in line with the [GABA]_e_ range reported earlier in hippocampal area CA1.^66^ Are these levels of [GABA]_e_ compatible with the peak values of *G*_tonic_ documented here using whole-cell recordings? One factor associated with having significant *G*_tonic_ is relatively low-affinity, slowly desensitized extrasynaptic GABA_A_Rs. The broadly expressed high-affinity δ-subunit-containing GABA_A_Rs were found to become desensitized and almost irresponsive to GABA when [GABA]_e_ reached ∼0.25 μM.^67^ Other receptor sub-types with EC_50_ in the range of 0.5–2 μM include native metabotropic GABA_B_ receptors in L2/3 pyramidal neurons^68^ and receptors with subunits α5β3γ2L (0.5–7 μM) and α1β2γ2L (0.6–9 μM) that can generate non-synaptic GABA currents in the hippocampus.^69^ Overall, it appears plausible that the [GABA]_e_ waves reported here find enough receptor targets to provide a significant dynamic range of *G*_tonic_.^13^ Indeed, we did not observe a consistent or progressive (post-pulse) reduction in IIE-synchronized GABAergic currents, for typical inter-spike intervals, throughout our electrophysiological recordings. This suggested no persistent GABA_A_R desensitization, which is also consistent with the desensitization kinetics of hippocampal GABA_A_R shown earlier,^70^ given the inter-IIE intervals and the [GABA]_e_ range observed here.

### Mechanisms controlling [GABA]_e_

GABA that escapes from synaptic clefts during interneuronal discharges is the most likely source of [GABA]_e_, although there have also been studies reporting GABA release from astroglia.^71^^,^^72^^,^^73^ One theoretically plausible mechanism that might regulate [GABA]_e_ is the reversal mode of GABA transporters.^74^ Four GABA transporter types (GAT1-3 and BGT1), all showing potassium- and voltage-dependent kinetics, are expressed in both neurons and glial cells.^75^^,^^76^^,^^77^ Intense excitatory activity—for instance, during epileptiform bursts—can increase extracellular [K^+^] to 12–15 mM^78^^,^^79^ and could, in theory, reverse GABA transport, thus generating GABA efflux.^80^ Intriguingly, while cultured neurons show depolarization-dependent GABA efflux via GAT-1 under negligibly low [GABA]_e_,^74^ any significant level of [GABA]_e_ should prevent the reverse GAT-1 mode.^54^ Because resting [GABA]_e_, in our experiments, is in the range of 0.1–0.3 μM (see above), any significant transporter-mediated GABA release is unlikely. Finally, regulation of [GABA]_e_ could arise from the Bestrophin 1 Ca^2+^-dependent Cl^−^ channel^81^ found predominantly in glial cells.^82^ However, this mechanism is unlikely to play a role here, as the operation of this channel does not appear correlated with neuronal activity.^81^

### Constraining parameters of simulated neural networks

Our study explores a well-established FS interneuronal network model^83^ that incorporates *G*_tonic_ driven by activity-dependent [GABA]_e_.^15^^,^^40^ While replicating the key aspects of interictal hippocampal activity, this model has certain limitations. First, we used a near-linear relationship between [GABA]_e_ and *G*_tonic_, whereas in reality it incorporates a more complex dependence.^12^^,^^15^ This might explain why the experimental time courses of [GABA]_e_ and *G*_tonic_ do not necessarily match. Second, to isolate the role of FS interneuronal networks in pacing hippocampal oscillations, the majority of our models mimicked PC activity by an artificial, interneuron-independent stochastic excitatory input. However, introducing a network that contained both interneurons and PCs (Figure 3) revealed spiking behaviors that were fully consistent with the key features of interneuron-only networks, as well as with experimental observations. Finally, astroglial GABA uptake must play an important role in pacing network rhythms as it shapes the [GABA]_e_ waveform. It was a revelation that our model could faithfully reproduce the relationship between the GABA uptake rate and the frequency of epileptiform events documented experimentally (Figure 7). This observation also implicated GAT-1, probably both astroglial and neuronal, as a major player in pacing rhythmic activities of interneuronal ensembles.

### Interneurons and excitatory action in rhythm initiation

In high-K^+^ experimental models, fluctuating cell depolarization provides a stochastic excitatory drive for interneurons. In the intact brain, however, this role belongs with PC firing, which must be an important contributor to epileptiform activity. Our experiments consistently point to an increase in tonic inhibition prior to an IIE onset. This invokes two possibilities: either interictal activity is due to the synchronized disinhibition of PCs or interneuron firing itself drives IIEs. We find that synchronized interneuronal network activity can determine the timing of coordinated pyramidal cell bursts, confirming the former suggestion.^84^^,^^85^ At the same time, interneuronal activity per se can make an important contribution to epileptiform discharges. Indeed, another potentially important mechanism regulating GABAergic excitation is HCO3^−^ transport^86^^,^^87^: its potential involvement in modulating IIE rhythms remains to be established.

It is important to highlight some well-documented mechanisms that drive interneuronal network oscillations and may not depend on [GABA]_e_ fluctuations. First, the so-called giant depolarizing potentials, the wave-like, second-long events occurring at 0.1–0.2 Hz during development.^88^^,^^89^^,^^90^^,^^91^^,^^92^ GDPs are thought to involve the synergistic activity of pyramidal cells and interneurons.^1^ The other mechanism involves high-frequency hippocampal sharp-wave ripples,^93^^,^^94^^,^^95^ representing highly synchronous activity bursts. In the ventral hippocampus, such ripples can occur in a periodic pattern, with a frequency of 7–14 Hz,^96^ similar to the theta rhythm. These oscillatory patterns could arise purely from the interaction of excitatory and GABAergic neurons, a mechanism qualitatively different from interictal activity resulting from periodic [GABA]_e_ waves. Inevitably, although FS PV+ interneurons have been considered major players in epileptiform activity, other interneurons, PCs, and non-neuronal cell types play their own important parts that could differ among different brain regions.

## STAR★Methods

### Key resources table

**Table:** 

REAGENT or RESOURCE	SOURCE	IDENTIFIER
**Bacterial and virus strains**		

AAV2/9-syn-iGABASnFR2-WPRE	GENIE Project Team & Looger Lab	This Paper
AAV2/9-GFAP-iGABASnFR2-WPRE	GENIE Project Team & Looger Lab	This Paper

**Biological samples**		

Sprague Dawley Rats Postnatal Day 5-8 (Organotypics)	Envigo	RRID: MGI:5651135
C57BL/6 Mice Postnatal Day 5-8 (Organotypics)	Envigo	RRID: MGI:2159769
PV::cre mice (B6;129P2-Pvalb^tm1(cre)Arbr^/J) (Acute slices)	Jackson laboratory	RRID: IMSR_JAX:008069
Mice floxed-stop tdTomato (B6.Cg-Gt(ROSA)26Sortm9(CAG-tdTomato)Hze/J) (acute slices)	Jackson laboratory	RRID: IMSR_JAX: 007909
Mice cre-dependent EYFP-tagged excitatory opsin channelrhodopsin-2-H134R (B6;129S-Gt(ROSA)26Sortm32(CAG-COP4^∗^H134R/EYFP)Hze/J) (acutes slices)	Jackson laboratory	RRID: IMSR_JAX: 012569

**Chemicals, peptides, and recombinant proteins**		

Minimal Essential Media	Thermofisher	Cat # 21090-022
HBSS	Thermofisher	Cat # 14185-045
B27	Thermo Fisher	Cat # 12587010
HEPES solution	Merck	Cat # H0887-20ML
Penicillin/Streptomycin	Thermofisher	Cat # 15070063
Ascorbic Acid	Merck	Cat # A5960-25G
Glucose	Merck	Cat # G8270-100G
Horse Serum	Merck	Cat # H1270-100ML
Molecular Probes Alexa Fluor 488 Hydrazide	Fischer Scientific	Cat# 10296832
NO-711	Biotechne/Tocris	Cat# 1779
NBQX disodium salt	Biotechne/Tocris	Cat# 1044/1
D-AP5	Biotechne/Tocris	Cat# 0106
Picrotoxin	Biotechne/Tocris	Cat# 1128
CGP 52432	Biotechne/Tocris	Cat# 1246
GABA	Merck	Cat # A5835-10G

**Experimental models: Organisms/strains**		

Sprague Dawley Rats	Envigo	RRID: MGI:5651135
C57BL/6 Mice	Envigo	RRID: MGI:2159769
PV::cre mice (B6;129P2-Pvalb^tm1(cre)Arbr^/J)	Jackson laboratory	RRID: IMSR_JAX:008069
Mice floxed-stop tdTomato (B6.Cg-Gt(ROSA)26Sortm9(CAG-tdTomato)Hze/J)	Jackson laboratory	RRID: IMSR_JAX: 007909
Mice cre-dependent EYFP-tagged excitatory opsin channelrhodopsin-2-H134R (B6;129S-Gt(ROSA)26Sortm32(CAG-COP4^∗^H134R/EYFP)Hze/J)	Jackson laboratory	RRID: IMSR_JAX: 012569

**Recombinant DNA**		

pAAV.hSynap.iGABASnFR	Loren Looger Lab	RRID: Addgene_112159
pAAV.hSynap.iGABASnFR2	GENIE Project team/Looger Lab	This Paper

**Software and algorithms**		

MES v4.x-v.6.3	Femontics	RRID: SCR_018309
MESc 3.57	Femontics	https://femtonics.eu/femtosmart-software/
Axon PClamp	Molecular Devices	RRID: SCR_011323
MATLAB	Mathworks	RRID: SCR_001622
Fluorescent Imaging Analysis Software (FIMAS)	https://github.com/zhengkaiyu/FIMAS	RRID: SCR_018311
Python Programming Language	Python, Anaconda	RRID: SCR_008394
WinEDR, Strathclyde Electrophysiology Software	Strathclyde University	N/A
OriginPro	OriginLab	RRID: SCR_014212
Network simulation software	In-house	https://github.com/LeonidSavtchenko/Arachne

**Other**		

Multiclamp 700B	Molecular Devices	RRID: SCR_018455
Digidata 1550	Molecular Devices	Digidata 1550
Femto3D-RC Multiphoton scanning microscope	Femontics	Femto3D-RC
Femto2D Multiphoton scanning microscope	Femontics	Femto2D
Femtosmart microscope	Femontics	Femtosmart
Leica VT1200S vibrating microtome	Leica BioSystems	RRID: SCR_020243
BioRad Helios Gene Delivery System	Bio Rad	RRID: SCR_019723
pE-2 LED illumination system	CoolLED	N/A
Slicescope Pro 2000	Scientifica	RRID: SCR_018405
Patchstar Manipulator	Scientifica	N/A

### Resource availability

#### Lead contact

Further information and requests for resources and reagents should be directed to and will be fulfilled by the lead contact, Dmitri Rusakov (d.rusakov@ucl.ac.uk).

#### Materials availability

This study did not generate new unique reagents.

### Experimental model and subject details

#### Animal experimentation

All experiments involving animals were carried out in accordance with the European Commission Directive (86/609/EEC) and the United Kingdom Home Office (Scientific Procedures) Act (1986), under the Home Office Project Licence PPL P2E0141 E1.

#### Experimental model designs across preparations

In our experimental protocols, we attempted to deviate from the 'normal' physiological conditions as little as possible, to induce similar types of epileptiform activity, rather than reproducing exactly the same protocol that could lead to distinct phenomena depending on the preparation. For instance, we used 0 mM [Mg^2+^] and 5 mM [K^+^] with no glutamatergic transmission blockade to induce interictal spikes, but had to increase [K^+^] to 10 mM to induce GABA waves with glutamatergic transmission blocked. In organotypic slices. 0 mM [Mg^2+^] was sufficient to trigger interictal events without the need to raise [K^+^], as further detailed below. To focus on a stable spiking regime, under equilibrated [K^+^], a brief period of several initial spikes was not considered for analyses.

#### Acute slice preparation

*In vitro* electrophysiological recordings were performed in acute hippocampal slices prepared from 3- to 4-week-old male Sprague-Dawley rats (Harlan Laboratories Inc, Bicester, UK). Animals were kept under standard housing conditions with 12h : 12h light-dark cycle and free access to food pellets and drinking water. After being sacrificed using an overdose of isoflurane, animals were decapitated, brains were rapidly removed, and hippocampi were dissected for slice preparation. Transverse hippocampal slices (350 μm-thick) were cut with a Leica VT1200S vibratome (Germany) in an ice-cold sucrose-based solution containing (in mM): sucrose (70), NaCl (80), KCl (2.5), MgCl_2_ (7), CaCl_2_ (0.5), NaHCO_3_ (25), NaH_2_PO_4_ (1.25), glucose (22), bubbled continuously with 95% O_2_ + 5% CO_2_ to yield a pH of 7.4. Slices were allowed to recover in a sucrose-free artificial cerebrospinal fluid (aCSF) solution (in mM): NaCl (119), KCl (2.5), MgSO_4_ (1.3), CaCl_2_ (2.5), NaHCO_3_ (26.2), NaH_2_PO_4_ (1), glucose (22), bubbled with 95% O_2_ and 5% CO_2_ in an interface chamber for at least 1 hour at room temperature before being transferred to a submerged recording chamber.

Modified aCSF was used to induce epileptiform activity: nominally 0 mM Mg^2+^ and 5 mM / 10 mM K^+^ under intact / blocked glutamatergic transmission, as specified. To facilitate the rapid generation of epileptiform discharges slices were perfused with the solution on both sides. All recordings were done at 32°C. GABA_B_ receptors were blocked in all experiments by 5 μM CGP52432. Field potential recordings from *stratum pyramidale* were performed with 1-2 MΩ glass electrodes filled with aCSF. Visualized whole-cell voltage-clamp recordings were performed from CA1 pyramidal neurons using an infrared differential interference contrast imaging system. Recording pipettes (3-5 MΩ) were filled with internal solution, containing (in mM): Cs-methanesulfonate (120), HEPES (10), EGTA (0.2), NaCl (8), MgCl_2_ (0.2), Mg-ATP (2), Na-GTP (0.3), QX-314 Br^-^ salt (5), pH 7.2, osmolarity 290 mOsm kg^-1^. GABA_A_R-mediated currents were recorded from neurons voltage clamped at 0 mV or +10 mV (close to the reversal potential of glutamatergic currents). To further prevent the contribution of NMDA receptors MK801 (1 μM) was included in the recording pipettes in acute slices experiments. CA3 area was cut off in the outside-out patch experiments, but not in other settings: this had no detectable effect on interneuronal network activity in area CA1.

Series resistance (R_s_) was monitored throughout the experiment using a –5 mV step command. Cells showing a >20% change in R_s_, or values >25 MΩ, or an unstable holding current, were rejected. Recordings were obtained using a MultiClamp 700B amplifier (Molecular Devices, CA, USA), filtered at 4 kHz, digitized and sampled through an AD converter Digidata 1550 (Molecular Devices) or NI PCI-6221M (National Instruments) at 10 kHz and stored on a PC. Data acquisition and off-line analysis were performed using WinEDR 3.0.1 (University of Strathclyde, Glasgow, UK) and Clampfit 10.0 (Molecular Devices Corporation, USA) software, or Femtonics MES (Femtonics, Budapest) and custom-made MATLAB (Mathworks) scripts running in MATLAB r2020b.

#### Organotypic slice culture preparation and biolistic transfection of iGABASnFR2

Organotypic hippocampal slice cultures were prepared and grown with modifications to the interface culture method from P6–8 Sprague-Dawley rats as previously described^48^ using the Stoppini interface method, which ensures no significant spontaneous activity in baseline conditions. In brief, 300 μm thick, isolated hippocampal brain slices were sectioned using a Leica VT1200S vibratome in ice-cold sterile slicing solution consisting of (in mM) Sucrose 105, NaCl 50, KCl 2.5, NaH_2_PO_4_ 1.25, MgCl_2_ 7, CaCl_2_ 0.5, Ascorbic acid 1.3, Sodium pyruvate 3, NaHCO_3_ 26 and Glucose 10. Following washes in culture media consisting of 50% Minimal Essential Media, 25% Horse Serum, 25% Hanks Balanced Salt solution, 0.5% L-Glutamine, 28 mM Glucose and the antibiotics penicillin (100U/ml) and streptomycin (100 μg/ml), three to four slices were transferred onto each 0.4 μm pore membrane insert (Millicell-CM, Millipore, UK). Cultures were then maintained at 37°C in 5% CO_2_ and fed by medium exchange every 2-3 days for a maximum of 21 days in vitro (DIV). At 5DIV cultures were treated overnight with 5 μM cytosine arabinoside (Ara-C, a poison inhibitor of DNA and RNA polymerases) to reduce glial reaction following biolistic transfection and returned to standard culture media at 6DIV. At 8DIV cultures were shot with 1.6 micron Gold micro-carriers coated with 30 μg of hSyn-iGABASnFR2 plasmid using the Helios gene-gun system (Bio-Rad). Following transfection cultures remained for 5-10 days before experiments were carried out. In this preparation, modified aCSF with 0 [Mg^2+^] (without raised [K^+^]) was sufficient to trigger interictal activity.

### Method details

#### Viral transduction of iGABASnFR2 for acute slice experiments

We used viral transduction of the optical GABA sensor in C57BL/6J mice (Charles River Laboratories), as detailed earlier, either in neonatal pups^47^ or in adult animals.^49^ Briefly, an AAV virus expressing the optical GABA sensor, iGABASnFR2 (Janelia Research Campus), under either neuronal (*hSyn*) or astroglial (*GFAP*) promoter, as indicated, was injected into the cerebral ventricles of neonates (P0-P3 of either sex) during aseptic surgery. The viral particles were injected at ∼1.5-2 μl per hemisphere (3-5 x 10^9^ genomic copies in total), at a rate not exceeding 0.2 μl/s, guided to a location approximately 0.25 mm lateral to the sagittal suture and 0.50–0.75 mm rostral to the neonatal coronary suture and 2 mm ventral from the surface of the cranium. After pups received AAV injections, they were returned to the mother in their home cage. The animals were systematically kept as a group of litters and monitored for several days thereafter to ensure that no detrimental effects appeared. Imaging experiments were performed 3 to 5 weeks after the injections, at which point acute hippocampal slices were prepared, as described above.

For the viral transduction of iGABASnFR2 in adults, young C57BL/6J mice (1–2-month-old), male and female, were prepared for aseptic surgery and anesthetized with isoflurane (3.5-4% v/v induction). The scalp was shaved and disinfected using topical chlorhexidine; topical lidocaine/prilocaine emulsion (2.5%) and ocular ointment (Lacri-lube, Allergan, UK) were applied. The animal was secured in a stereotaxic frame, and a stable anesthesia level (maintenance at 1.5-2.5% isoflurane) was confirmed before starting surgery. Body temperature was maintained at ∼37.0°C using a feedback rectal thermometer and heating blanket. Perioperative analgesics were administered (subcutaneous buprenorphine, 60 μg kg^-1^). A small, vertical incision was made to expose the skull. A craniotomy of approximately 1 mm diameter was performed over the right hemisphere using a high-speed drill, at a site overlying the medial hippocampus. Stereotactic coordinates were 2.5 mm of the anteroposterior distance from bregma to lambda and 2.5 mm lateral to the midline. Pressure injections of AAV9-hsyn-iGABASnFR2-WPRE or AAV9-GFAP-iGABASnFR2-WPRE (totaling 1 × 10^10^ genomic copies in a volume not exceeding 200 nL) were carried out using a Hamilton syringe needle stereotactically guided to a depth of 2.6 mm ventral from the cortical surface, at a rate of approximately 1 nL sec^-1^. The total injection volume was delivered in two steps, reducing the depth by 200 μm. Once delivery was completed, the needle was left in place for 5-7 minutes before being retracted. The surgical wound was closed with absorbable 6-0 sutures. Metacam (1 mg kg^-1^) and saline (0.5 ml) were subcutaneously administered, and the animal was left to recover in a heated chamber. Imaging experiments were performed 3 to 5 weeks after the injections. The procedure of cardiac perfusion of animals was performed under general anesthesia, using an ice-cold aCSF saturated with 95% O_2_ and 5% CO_2_, before dissecting the brain for the acute hippocampal slice preparations.

#### Outside-out patch recordings

Single channel GABA_A_R-mediated currents were recorded in the presence of 0.1 μM CGP-55845 as detailed earlier.^18^^,^^38^ Outside-out patches were pulled from dentate granule cells, raised above the slice surface and moved to area CA1; recordings were performed in voltage-clamp mode at 33–35°C. For membrane patches held at -70 mV, the recording electrode solution contained (in mM): 120.5 CsCl, 10 KOH-HEPES, 10 BAPTA, 8 NaCl, 5 QX-314-Br^-^ salt, 2 Mg-ATP, 0.3 Na-GTP; pH 7.2, 295 mOsm kg^-1^. For membrane patches held at 0 mV, the recording electrode solution contained (in mM): 120 Cs-methanesulfonate, 10 HEPES, 0.2 EGTA, 8 NaCl, 0.2 MgCl_2_, 2 Mg-ATP, 0.3 Na-GTP, 5 QX-314-Br^-^ salt; pH 7.2, 290 mOsm kg^-1^. Recordings were made using MultiClamp 700B amplifier (Molecular Devices, CA, USA); signals were digitized at 10 kHz. The patch pipette resistance was 5–7 MΩ. In experiments where GABA EC_50_ was determined, we used a θ-glass application pipette with ∼200 μm tip diameter attached to a micromanipulator. The pipette position was controlled by a piezoelectric element (each switch took 50–100 μs) to allow rapid solution exchange. One pipette channel was filled with the bath aCSF solution; another channel had aCSF with varying concentrations of GABA. The flow rate was driven by gravity.^44^

#### Analysis of single-channel recordings and iGABASnFR2 titration

The frequency of GABA_A_R channel openings was calculated as *N* /*Δt*, where *N* is the number of openings and Δt is the time of recording. *N* was counted using a detection threshold of 1.5 pA above the mean baseline value and a minimum opening time of >0.2 ms. Single channel conductance was calculated as *G = I / (V*_*rev*_ *- V*_*hold*_*)*, where *I* stands for receptor-mediated current, and *V*_*rev*_ – calculated chloride reversal potential. The average charge transfer through receptors was calculated as *Q* = *G*× *N* / *Δt*. The concentration-effect plot was fitted with the Hill equation $E=\frac{{\left[C\right]}^{n}}{{K_{d}}^{n}+{\left[C\right]}^{n}}$, where *E* stands for the percentage of maximal charge transfer, *C* concentration of GABA, *K*_*d*_ microscopic dissociation constant (equal to EC_50_), and *n* Hill's coefficient. A similar equation was applied to obtain the best-fit function for the iGABASnFR2 calibration (Figure 2D).

#### Two-photon excitation imaging

For imaging of iGABASnFR2 transients associated with interictal activity, organotypic slice cultures were cut from their membrane insert and transferred to the stage of a Femtonics Femto3D-RC, Femto-2D, or in some experiments, Femtosmart imaging system (Femtonics, Budapest) integrated with patch-clamp electrophysiology and linked on the same light path to two femtosecond pulse lasers MaiTai or Insight X3, respectively (SpectraPhysics-Newport) with independent shutter and intensity control as previously described.^48^ Imaging settings were adjusted to provide optimal recording conditions for the preparation. Both imaging and electrophysiology recordings were acquired from the microscope acquisition board at 40kHz; electrophysiology data were then decimated to 10kHz and data from the detectors processed by the scope to form frame scans at 10-50Hz. Normally, as the imaging frame rate is lower than the field potential acquisition rate, we sub-divide the imaging bins into equivalent-value bins to match the electrophysiology recording bins, and thus to align the two recordings accurately. Laser light intensity under the objective was adjusted to minimize photobleaching (<7 mW), at a wavelength of λ_x_^2p^ = 910 nm; control for photobleaching, pixel and optical resolution, were set and optimised as detailed previously.^48^^,^^97^ The iGABASnFR2 sensitivity was several-fold that of iGABASnFR^36^ providing a stable readout of resting (equilibrated) GABA-sensitive fluorescence between interictal spikes (Figure 2B): the *F*_0_ value was measured as the average baseline intensity over a ∼1 s time window.

Slices were continuously perfused with bicarbonate based artificial cerebrospinal fluid (aCSF) equilibrated with 95% O_2_ and 5% CO_2_ at 32–34 °C using a gravity driven perfusion system (flow rate 3-4ml/min). aCSF solution contained (in mM): 120 NaCl, 10 glucose, 2.5 KCl, 1.3 MgSO_4_, 1 NaH_2_PO_4_, 25 NaHCO_3_, 2 CaCl_2_ with osmolarity of 300 ± 5 mOsm kg^-1^. Biolistic transfection of iGABASnFR2 resulted in sparse labeling of neurons in the CA1 and CA3 regions making identification of iGABASnFR2 expressing cells using two-photon imaging (λ_x_^2p^ = 910 nm) unambiguous. Following identification, a glass field recording electrode filled with standard aCSF was placed within ∼30 μm of the cell of interest and interictal activity was then initiated by exchanging the extracellular solution for a 0 mM Mg^2+^ aCSF (otherwise the same composition).

The local field potential was then monitored until stable interictal bursting was observed, at which point curved frame-scan regions of interest following the somatic membrane were chosen for scanning (see Figure 2A for illustration). To avoid photodamage, scan duration was limited to 9 second epochs which were repeated with a one-minute intervals until a minimum of 10 events were captured. For comparison of interictal event timing and associated iGABASnFR2 transients; interictal event peak times were first identified by the *findpeaks* function in MATLAB and were accepted for further analysis where single peaks with a minimum width of 20ms and amplitude >5 x standard deviation (SD) of baseline noise could be detected. The associated iGABASnFR2 ΔF/F_0_ peak was then identified (again using *findpeaks*) within a manually adjusted window -750 ms to + 2s (extremes between cells/slices) over the peak times identified for the interictal event, with a minimum >2.5 x SD baseline and minimum width of 50ms. Events within that window were then analyzed where a clear and stable iGABASnFR ΔF/F_0_ 50ms length baseline, rise and visible decay associated could be observed. The rise to peak from baseline was then fitted by polynomial regression and the 20% iGABASnFR2 peak time extrapolated from the fit and used to calculate iGABASnFR transient-interictal event lag times.

#### Targeted cell-attached recordings and optogenetic experiments

*PV*-Cre mice (B6;129P2-Pvalb^tm1(cre)Arbr^/J Jackson laboratory stock number: 008069) were crossed with Ai9 or Ai32 mouse line, which has floxed-stop tdTomato (B6.Cg-Gt(ROSA)26Sortm9(CAG-tdTomato)Hze/J Jackson laboratory stock number: 007909) or EYFP-tagged excitatory opsin channelrhodopsin-2-H134R (B6;129S-Gt(ROSA)26Sor^tm32(CAG-COP4∗H134R/EYFP)Hze^/J Jackson laboratory stock number: 012569), to produce animals expressing either tdTomato or channelrhodopsin-2 (ChR2) in parvalbumin-positive (PV+) interneurons throughout the brain.^98^ Animals were kept under standard housing conditions with 12 h: 12 h light-dark cycle and free access to food pellets and drinking water. Hippocampal slices were prepared from mice of both sexes aged between postnatal day 25 and 50 for the electrophysiology section, but under low light intensity to minimize any adverse effect due to activation of ChR2. Field potential recordings from the *stratum pyramidale* and visualized whole-cell voltage-clamp recordings were performed from CA1 pyramidal neurons using infrared differential interference contrast imaging system as in the electrophysiology section. For the dual cell-attached recordings (Figure 5), borosilicate glass pipettes (3-5 MΩ) filled with aCSF and containing Alexa Fluor 488 (50 μM) were used and the whole-cell recording pipettes was containing the standard Cs-methanesulfonate cited above with the addition of Alexa Fluor 488. In addition, the slices were superfused with aCSF with 0 mM Mg^2+^ and 10 mM K^+^ as well as APV (50 μM), NBQX (10 μM) and CGP52432 (1 μM); the GABA_A_R blocker picrotoxin (100 μM) was added where indicated.

Cells showing a series resistance >25 MΩ, or an unstable holding current, were rejected. Recordings were obtained using a MultiClamp 700B amplifier (Molecular Devices, CA, USA), filtered at 2-4 kHz and digitized at 10 kHz. Data acquisition and off-line analysis were performed using WinEDR 3.0.1 (University of Strathclyde, Glasgow, UK) and Clampfit 10.0 (Molecular Devices Corporation, USA) software as well as MATLAB and Python custom-scripts.

Wide-field illumination of the CA1 region of the hippocampus was delivered through a 20x water immersion objective (Olympus). PV tdTomato-positive cells were visualized with 590 nm wavelength and ChR2 was activated by blue light (wavelength 470 nm) generated by pE-2 LED illumination system (CoolLED); light intensity under the objective was in the range of 5 - 10 mW. For the ramp stimulation protocol, a train of 1 s ramps was delivered at on average ∼0.3 Hz ranging from 0.12 to 0.33 Hz.

In some experiments (Figure 4), neurons showing the 'wave-like' activity were defined as follows. The voltage clamp trace was lowpass filtered with an IIR Butterworth filter at a cut-off frequency of 2Hz. The FFT was then computed on the filtered trace and if a significant (above 95CI) peak was present on the power spectrum between 0.01-2Hz, the activity was considered as wave-like.

#### Interictal discharge analysis

The detection of interictal spikes was done using custom-made python script. Local field electrophysiological trace was first downsampled to 1 kHz and filtered using a Butterworth bandpass filter from 1 to 50 Hz. The onset of interictal discharges was determined by using a detection threshold on the second derivative of the local field potential recording. The threshold corresponded to a minimum of 4 SDs above baseline noise (taking from the beginning of each trace). For the probability of inducing an interictal events with 1 ms light pulse stimulation, a window of 100 ms post-stimulation was considered for the detection of an event. Concerning the ramp stimulation, the window was between 500 ms after the start of the ramp and 100 ms post-stimulation.

#### Modeling: Networks of interneurons and pyramidal neurons

The interneurons and pyramidal neuron (principal cell, PC) networks were simulated on a digital platform ARACHNE with remotely-controlled parallel computing.^41^ Similar to the previously explored non-hierarchical networks,^15^ the present one featured circular connectivity (Figure 3A), which helped (a) exclude edge effects, (b) equalize cell contribution, and (c) represent the size by a single parameter, network radius *R*. In individual simulated cells, the channel kinetics were typical of hippocampal fast-spiking basket interneurons.^83^ Other incorporated biophysical mechanisms, such as ion channel flux, GABA transporter, synaptic and GABA tonic current were in accord with experimental findings,^99^^,^^100^^,^^101^ as detailed below. The time course of GABAergic synaptic conductance followed the function $y\left(t\right)=G_{ii}\left(\exp\left(-t/\tau_{1}\right)-\exp\left(-t/\tau_{2}\right)\right)$ where *y*(*t*) is the synaptic conductance at time *t*, *τ*_1_ is the rise time constant, *τ*_2_ (termed tau elsewhere) is the decay time constant, *G*_*ii*_ is the peak conductivity (mS cm^-2^) of synapses.

A network of interneurons and pyramidal cells was simulated using previously published models,^99^^,^^100^ which was further developed as a full-scale cloud-computing platform ARACHNE,^41^ with minor modifications of the internal parameters. The critical addition was the dynamic *G*_tonic_, as explained below, with the *G*_tonic_ range in interneurons twice that of pyramidal cells, in accord with experimental data^15^; the basic cell-circuit model used in the present study was obtained from ModelDB (accession no. 138421). Briefly, the modelled circular network consisted of *N* fast-spiking interneurons (I-cells) and *M* pyramidal cells (E-cells). Each cell was modelled as a single compartment using standard Hodgkin–Huxley formalism. The GABA_A_R reversal potential *V*_*GABA*_ was set between –55 mV and –72 mV, as specified. Unless specified otherwise, the network featured three connection types: *I–I*, *I–E*, and *E–I*, with release probabilities values *P*_*E–E*_ = 0.5, *P*_*E–I*_ = 0.5, and *P*_*I–I*_ = 1.0, respectively. The spatial synaptic connection density was defined as the matrix *W*_*XY*_ (*i,j*) with the Gaussian distribution centered at a given neuron, so that

$$W_{XY}\left(i,j\right)={W_{XY}}_{\max}\;\exp\left(-\frac{1}{2}{\left(\frac{\max\left(i,j\right)-1}{\sigma_{xy}}\right)}^{2}{\left(\frac{j-1}{\max\left(j\right)}-\frac{i-1}{\max\left(i\right)}\right)}^{2}\right)$$

The model consists in bell-shaped strength and Gaussian density of connections. Synaptic conductance matrices of this model are with following strength coefficient *W*_*ee,*max_ = 2, *W*_*ii,*max_ = 0.8, *W*_*ei*,max_ = 0.9; *W*_*ie*,max_ = 0.3, and spatial SD: σ_ee_ =10 μm, σ_ei_ =12.5 μm, σ_ie_ =8 μm and σ_ii_ =5 μm. The size (radius) of interneuron and principal cell network was set at 250 and 200 μm, respectively, with the signal propagation speed at 0.1 μm ms^-1^.

Simulations were performed using ARACHNE on the Amazon AWS cloud computing (cluster c5.large, tolerance 10^−5^, time step 0.02 ms). Random generator *use32BitRng* (MATLAB) was set to generate a delta correlated white noise for any stochastic processes. The initial voltage of interneurons in the network was set uniformly randomly, between -73 and -67 mV. Mechanisms of synaptic plasticity were excluded from the basic model of the neural network.

#### Modeling: Network synchronization

The network synchronization parameter *k*(*τ*), which was also used for the experiment in PV+ cell pairs (Figure 4), was calculated as an average of all coefficients *k*_*i,j*_(*τ*) for each pair of neurons (*i, j*). The time window of synchronization was divided into bins so that *τ* = 0.1/*f*_*m*_ where *f*_*m*_ is the network-average spiking frequency (see below). In each bin, an action potential was represented in a binary format (yes-no series). Next, *k*_*i,j*_(*τ*) was calculated for each pair of neurons for the time bin *τ* using the formula:

$$k_{ij}\left(\tau \right)=\frac{\sum_{l=1}^{K}X\left(l\right)Y\left(l\right)}{\sqrt{\sum_{l=1}^{K}X\left(l\right)\sum_{l=1}^{K}Y\left(l\right)}}$$

where *X* and *Y* are the binary series of the *i*th and *j*th cells, respectively, *l* is the bin number; thus, *X(l)* and *Y(l)* are either 0 or 1 depending on having a spike (1) or no spike (0) event for *i*th or *j*th during the *l*th bin, and *K* is the total number of bins.

#### Modeling: Tonic GABA conductance

[GABA]_e_ activates extrasynaptic GABA_A_Rs, which generate *G*_tonic_. Thus, modelled cells generate non-specific tonic current: $I_{tonic}=G_{tonic}\left(V_{GABA}-V_{m}\right)$, where *V*_GABA_ is GABA_A_R reversal potential, and *V*_*m*_ is the membrane potential. *G*_*tonic*_ depends on the interneuron network firing frequency, which in turn generates [GABA]_e_ increments, in accord with:

(Equation 1) $$\frac{dG_{tonic}}{dt}=N_{i}\;A_{f}\left(f_{m}\left[t-dt\right]+f_{b}\right)-G_{pump}\left(G_{tonic}-G_{basal}\right)$$

with initial $G_{tonic}\left(0\right)=G_{basal}$ and *f*_*m*_ is calculated as the total of cell-generated spikes over time *T*:

(Equation 2) $$f_{m}=\frac{1}{T\;N_{i}}\sum_{i=1}^{N_{i}}AP_{i}\left(T,t\right)$$

Other parameters were: *N* (or *N*_*i*_), total number of neurons in the network; *A*_*f*_ (0.01 nS cm^-2^ ms^-1^, unless specified otherwise), tonic GABA conductance resulting from a single AP over 100 ms; *G*_pump_ (values as indicated), the GABA uptake rate, *G*_basal_ = 0.1 mS cm^-2^, [GABA]_e_ value at which GABA transporters reverse, *f*_*b*_ = 0.1 Hz, basal average network spiking frequency at rest; and *dt*, a delay between neurotransmitter release and activation of extrasynaptic receptors (*dt* was <1 ms throughout).

In our simulations, we assumed that *G*_*tonic*_ linearly depends on [GABA]_e_: *G*_tonic_ = α[GABA]_e_. This assumption provides a good approximation [GABA]_e_ < 1-2 μM, as in this case it falls into the near-linear approximation of the charge transfer versus [GABA]_e_ relationship (Figure 1E). The *G*_tonic_ dynamics was therefore determined by the time course of [GABA]_e_, which is represented by quasi-instantaneous release followed by uptake (i.e., quasi-exponential decay of [GABA]_e_ to its basal level). In the basic established model, the GABA transport reversal potential *V*_*GAT*_ is given by

$$V_{GAT}=\frac{RT}{F}\left(2\;\mathrm{Ln}\frac{{\left[Na\right]}_{out}}{{\left[Na\right]}_{in}}+\mathrm{Ln}\frac{{\left[GABA\right]}_{out}}{{\left[GABA\right]}_{in}}-\mathrm{Ln}\frac{{\left[Cl\right]}_{out}}{{\left[Cl\right]}_{in}}\right)\text{,}$$

which was explored previously, together with the estimates of the GAT-1 kinetics, in some detail,^54^ so that the solution of equation (1) for constant *f*_*m*_ was

$$G_{tonic}\left(t\right)=e^{-t_{p}\;t}G_{basal}+\frac{A_{f}f_{m}N_{i}}{t_{p}}e^{-t_{p}\;t}\left(e^{t_{p}\;t}-1\right)\text{,}$$

where *t*_*p*_ is 0.02–0.1 ms^-1^ and *A*_*f*_ is the *G*_basal_ / *G*_tonic_ ratio at steady-state for a given frequency *f*_*m*_: $A_{f}=\frac{KG_{init}t_{p}}{f_{m}N_{i}}$, where 2 < *K* < 4. Thus, *A*_*f*_ was a scaling factor for [GABA]_e_ action.

#### Modeling: [GABA]_e_ dynamics

The dynamics of [GABA]_e_ was estimated from the cell-spiking raster plot, with individual action potentials releasing GABA at a rate of 0.5 μM ms^-1^ of GABA (∼3000 molecules) in an extracellular volume of 20 μm radius, the average distance between neighbouring neurons of the network. [GABA]_e_ decays due to GABA uptake and diffusion, a typical constant of 0.004 μM ms^-1^ (Savtchenko et al.^54^), unless specified otherwise.

To explore the parameter space with respect to rhythm generation, and for the sake of clarity, we introduced a single dimensionless parameter *R* as a combined GABA release-uptake factor incorporating *A*_*f*_ and *G*_*pump*_, in accord with

$$\frac{d{\left[GABA\right]}_{e}}{dt}=\frac{G_{tonic}}{dt}=N_{i}\cdot R\cdot A_{f}\left(f_{m}\left[t-dt\right]+f_{b}\right)-R\cdot G_{pump}\left(G_{tonic}-G_{basal}\right)$$

were term notation is as described above.

#### Modeling: Code detail

ARACHNE is available with explanatory documentation at https://www.neuroalgebra.com/. The program is made available under MIT license. ARACHNE is written in a way that allows users to run it on any remote platforms. Files with a set of initial parameters for reproducing the results compatible with ARACHNE can be provided upon request. For any computations we provided the exe file of ARACHNE, but also the initial code on https://github.com/LeonidSavtchenko/Arachne.

### Quantification and statistical analysis

Shapiro-Wilk tests for normality were routinely run for small samples (this test for the means could be misleading for n > 15-19 due to Central Limit Theorem). Correspondingly, two-tailed paired and unpaired Student’s t-test, or otherwise non-parametric Mann-Whitney tests were used for statistical analyses. Mean difference was considered significant at the null-hypothesis rejection level of *p* < 0.05. Statistical summary data are shown as mean ± SEM unless specified otherwise. To account for the factors of slices versus recorded cell pairs, a Kruskal-Wallis non-parametric ANOVA was performed.

## Data Availability

• Data reported in this paper will be shared by the lead contact upon request. • The original code for network simulation is deposited at GitHub and is publicly available as of the date of publication. Doi is specified in the key resources table. • Any additional information required to reanalyze the data reported in this paper is available from the lead upon request.

## Supplemental information

Document S1. Figures S1–S7

Document S2. Article plus supplemental information
