# Supplementary material for: Volume-transmitted GABA waves pace epileptiform rhythms in the hippocampal network
Source: Curr Biol. 2023 Apr 10;33(7):1249–1264.e7. doi: 10.1016/j.cub.2023.02.051 (PMC10615848; doi:10.1016/j.cub.2023.02.051)
Supplement: Document S1. Figures S1–S7 [file mmc1.pdf]

Current Biology, Volume *33*

Supplemental Information

Volume-transmitted GABA waves pace

epileptiform rhythms in the hippocampal network

Vincent Magloire, Leonid P. Savtchenko, Thomas P. Jensen, Sergyi Sylantyev, Olga Kopach, Nicholas Cole, Olga Tyurikova, Dimitri M. Kullmann, Matthew C. Walker, Jonathan S. Marvin, Loren L. Looger, Jeremy P. Hasseman, Ilya Kolb, Ivan Pavlov, and Dmitri A. Rusakov

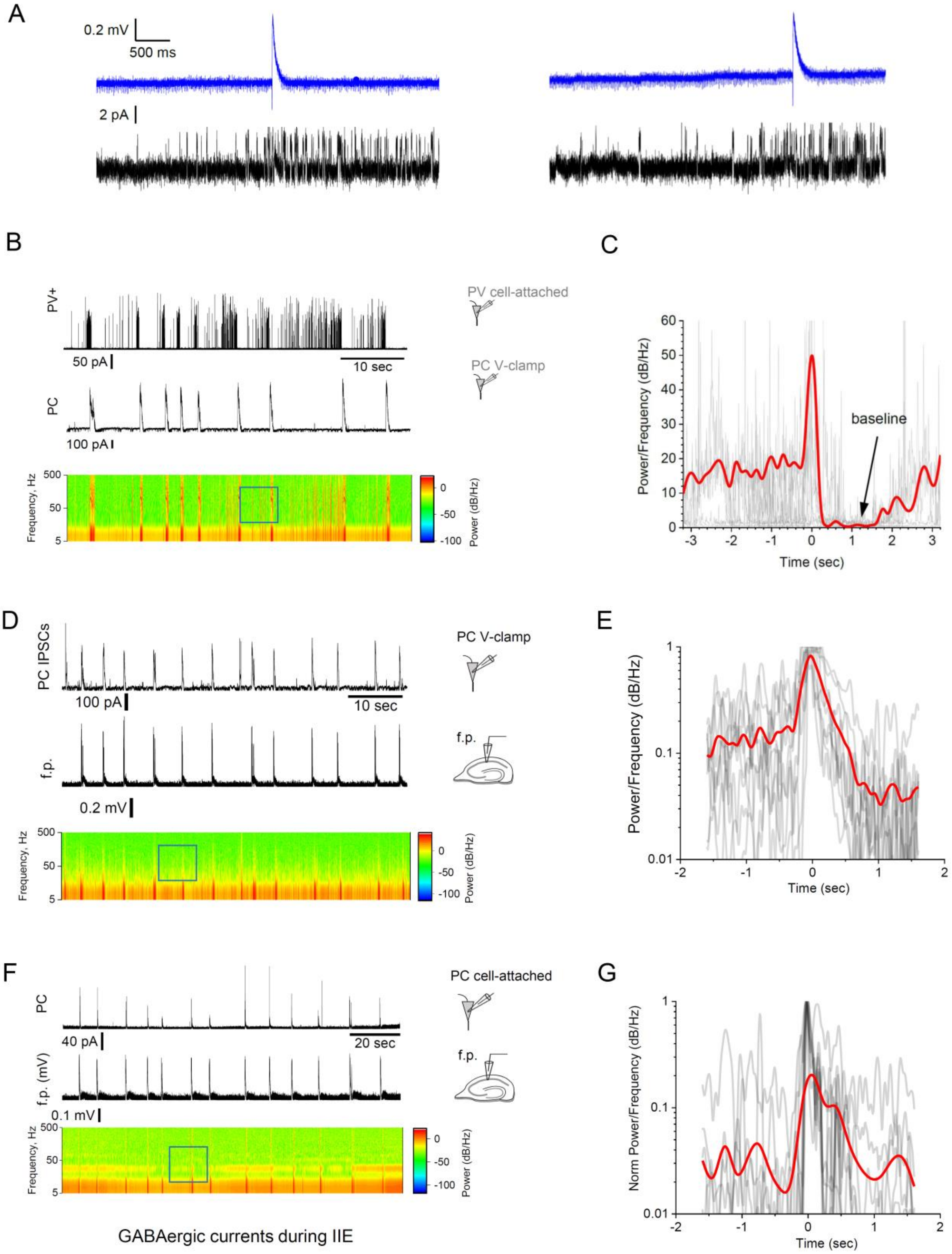

Figure S1. FS PV+ interneurons increase firing rate before inter-ictal events, Related to Figure 1.

(A) Examples of local field potentials (f.p., top trace) and sniffer-patch single-channel activity (bottom) before and after individual interictal discharges in hippocampal slices.

(B) Simultaneous cell-attached spiking recording in a FS PV+ interneuron (top) and whole-cell recording of $GABA_AR$ IPSCs in a pyramidal neuron (middle), with the power spectrum density of interneuronal spiking (bottom). Note that our analysis did not isolate IPSCs, it computed the Fourier transform of the continuous IPSC signal for a wide range of frequencies (the Wavelet Transform, 5-500 Hz) and then averaged the fast-spiking parts of the Wavelet pattern (10-200 Hz) centred at the pyramidal cell current peak onset; blue box, the area of averaging.

(C) Average power density across the spectrum, over the typical cycle of interictal spiking, centred at burst peak (blue square in B); red line, sample average; grey, individual interictal spike traces (n = 10, alignment at field potential spike onset, $t = 0$); arrow, post-spike returns to baseline, before the rise of spiking activity preceding an IIE.

(D) Simultaneous recordings of $GABA_AR$ IPSCs in pyramidal cells (top) and local f.p. (middle), the power spectrum density of IPSCs recorded in pyramidal cells (bottom).

(E) Graph as as in C, but for pyramidal cell spiking shown in D (n = 9).

(F) Simultaneous cell-attached recordings of a pyramidal neuron (top), and local field potential (f.p.) during interictal events, with the power spectrum density of pyramidal cell spiking (bottom).

(G) Graph as as in C, but for pyramidal cell spiking shown in F (n = 10).

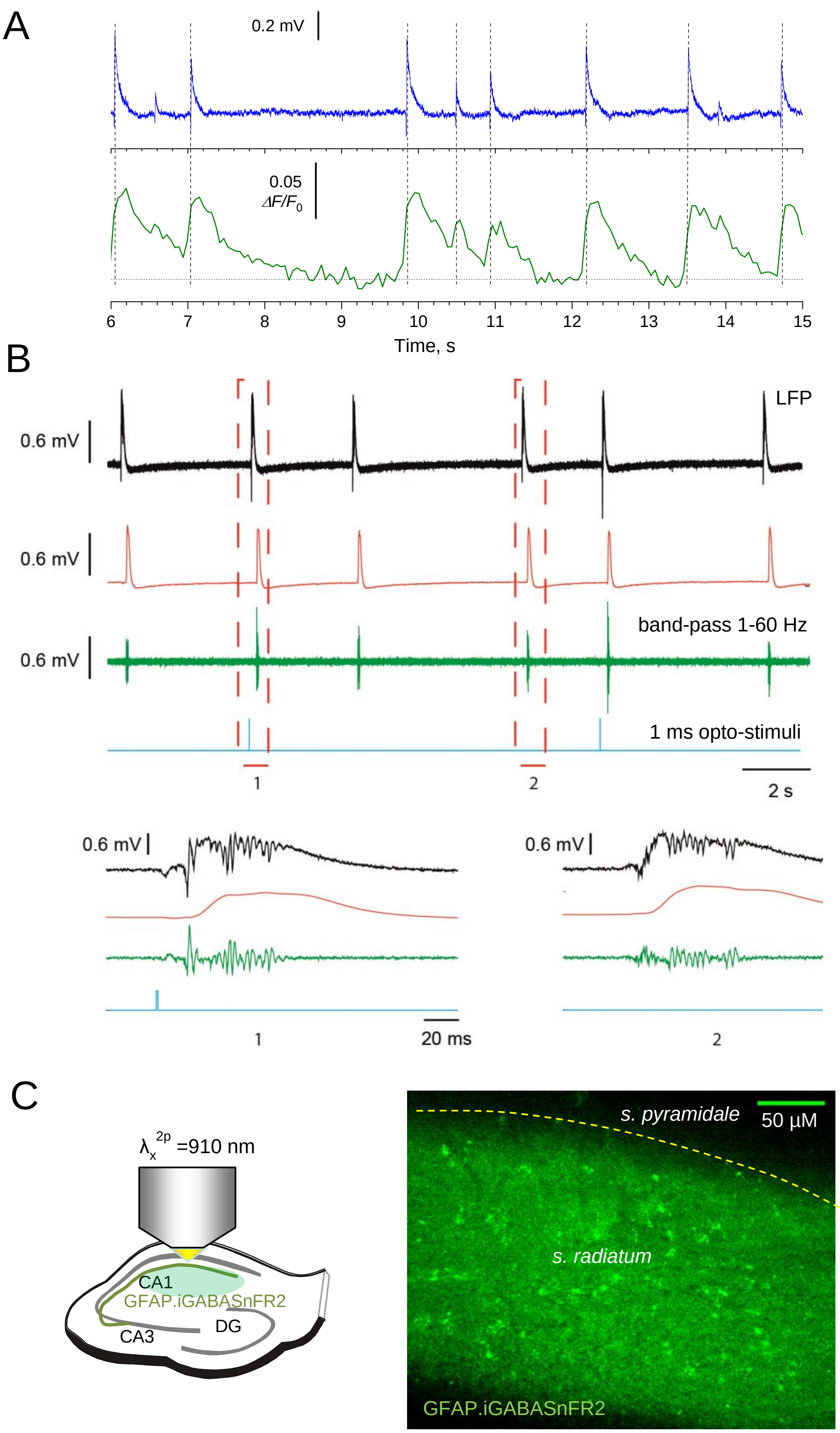

Figure S2. Monitoring interictal discharges and $[GABA]_e$, Related to Figure 2.

(A) Example of fEPSPs (top, blue) and iGABASnFR2 signal (bottom, green) recorded during interictal spikes; vertical dotted lines, onsets of interictal spikes (note that these lag behind the onset of [GABA] rises).

(B) Example to illustrate electrophysiological recordings of interictal activity, with lowpass filtered signals, showing no shift in field potential before the IIE onsets during either spontaneous or optogenetically evoked IIEs; this suggests no significant $[K^+]_{out}$ fluctuations in this context.

(C) Diagram, experimental design illustrating two-photon excitation imaging of astrocyte-expressed iGABASnFR2 in area CA1 of the acute hippocampal slice. Image, low-magnification view of the area CA1 *s. radiatum* (green fluorescence channel, ~100 µm z-stack average) showing relatively homogenous expression of iGABASnFR2; multiple astrocyte somata can be seen.

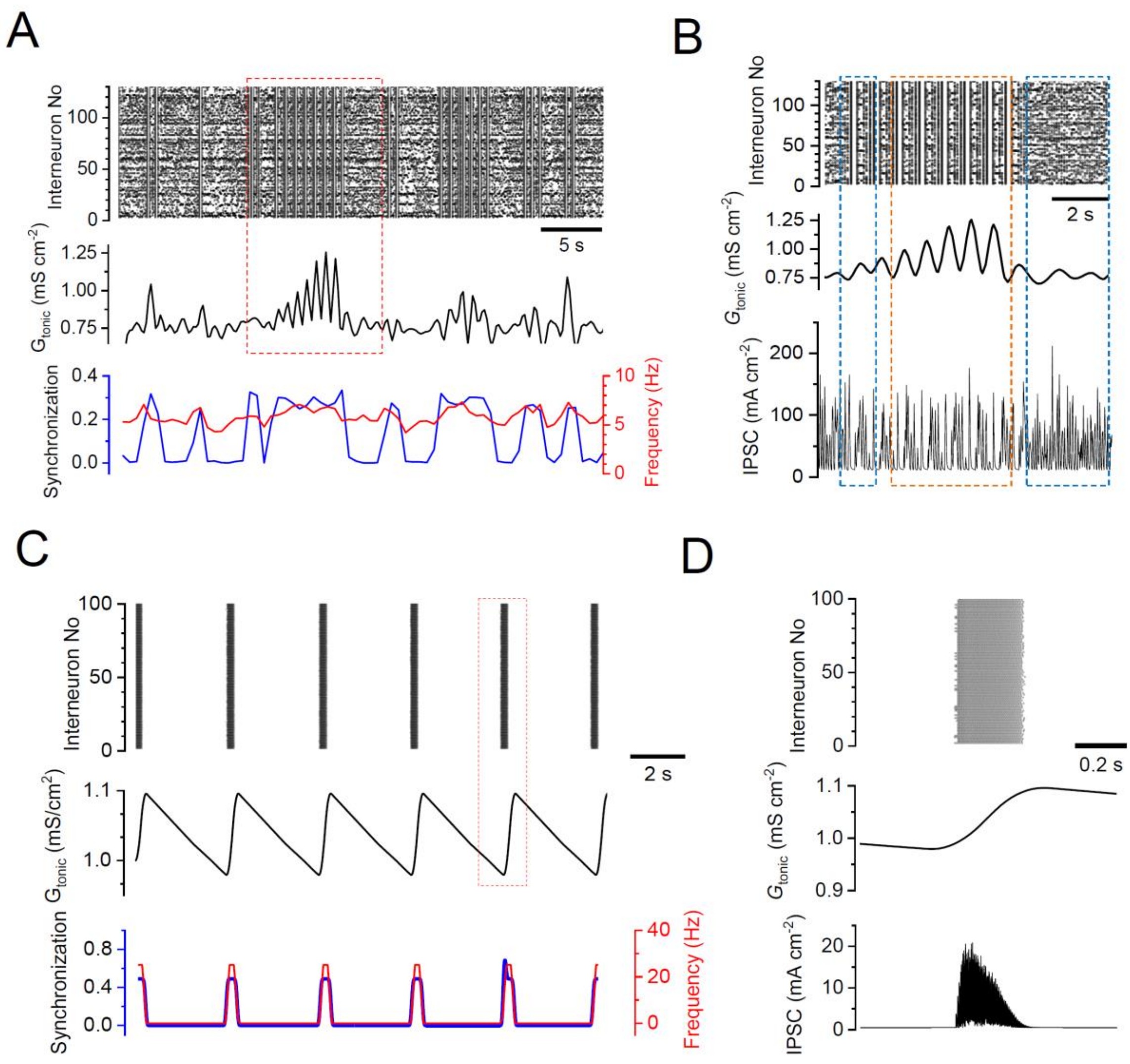

Figure S3. [GABA]$_e$-dependent G_{tonic} drives rhythmic activity of a modelled interneuron network, Related to Figure 3.

(A) Raster plot of interneuronal network spiking activity (top), G_{tonic} driven by [GABA]$_e$ (middle) calculated from integrated interneuronal discharges; and network synchrony and mean network frequency showing quasi-periodicity features (bottom). Key model parameters: cell number N = 130, intra-network peak synaptic conductance G_{ii} = 15 mS cm^{-2}; E-currents (Poisson series) with average synaptic conductance g_s=0.05 mS cm^{-2}, decay constant tau = 3 ms, and frequency f_s = 50 Hz; G_{tonic} reverse potential V_{GABA} = –55 mV, GABA release factor Af = 60 $\times 10^{-7}$ nS cm^{-2} ms^{-1}, G_{pump} =0.02 s^{-1} (see Methods for further detail).

(B) Fragment from A (red dotted rectangle) enlarged, with the IPSC series sampled from an arbitrarily selected simulated interneuron (bottom). Blue and orange rectangles, high-frequency non-synchronised, and oscillating and synchronised IPSC periods, respectively.

(C-D) Graphs as in A-B but with N = 100, g_s = 0.2 mS cm^{-2} , f_s = 50 Hz, G_{ii} = 0.02 mS cm^{-2}, V_{GABA} =–60 mV, A_f = 0.18$\times 10^{-7}$ nS cm^{-2} ms^{-1}, G_{pump} = 0.0004 s^{-1}.

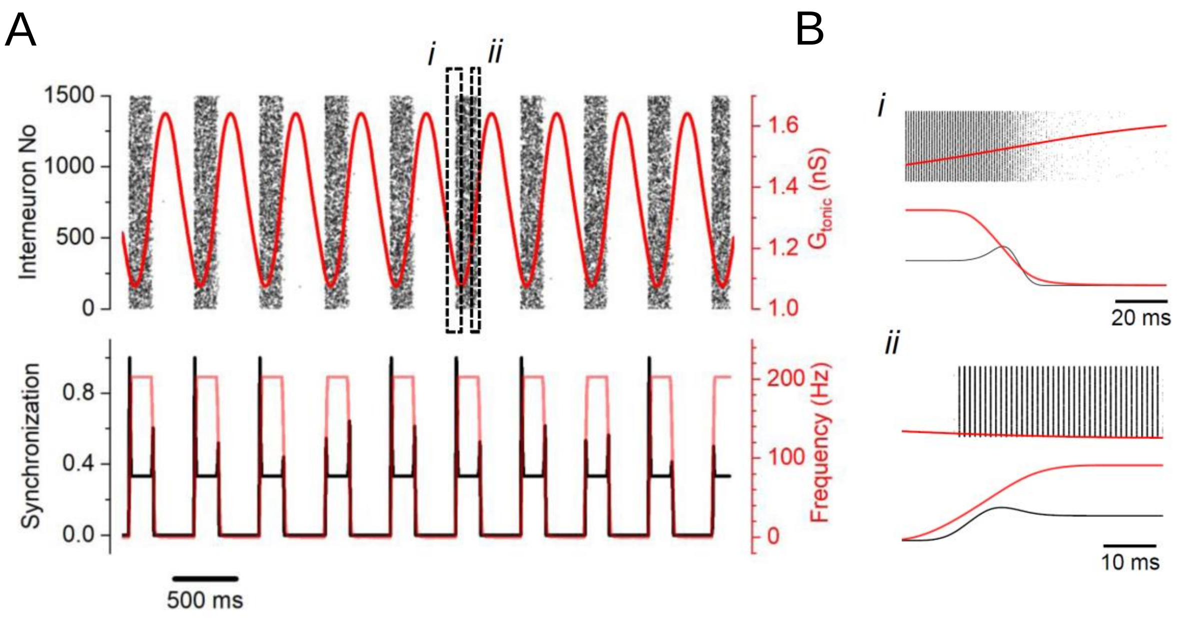

Figure S4. Increasing network size makes burst rhythms more robust, related to Figure 3.

(A) Raster plot of spiking network activity (top), network synchronisation coefficient (bottom, black), and time course of average spiking frequency (bottom, red) for the network as in Fig. 3c; key model parameters: cell number N = 1500, intra-network peak synaptic conductance G_{ii} = 15 nS cm^{-2}; E-currents (Poisson series) with average synaptic conductance g_s=0.05 nS, decay constant tau = 3 ms, and frequency f_s = 50 Hz; G_{tonic} reverse potential V_{GABA} = –55 mV, GABA release factor Af = 60 $\times10^{-7}$ nS cm^{-2} ms^{-1}, GABA uptake rate G_{pump} =0.02 s^{-1} (see Methods for further detail).

(B) Fragments *i* and *ii* from A (dotted rectangles), as indicated, on an expanded scale; note that synchronization (black line, bottom traces) roughly follows the first time derivative of frequency (red trace).

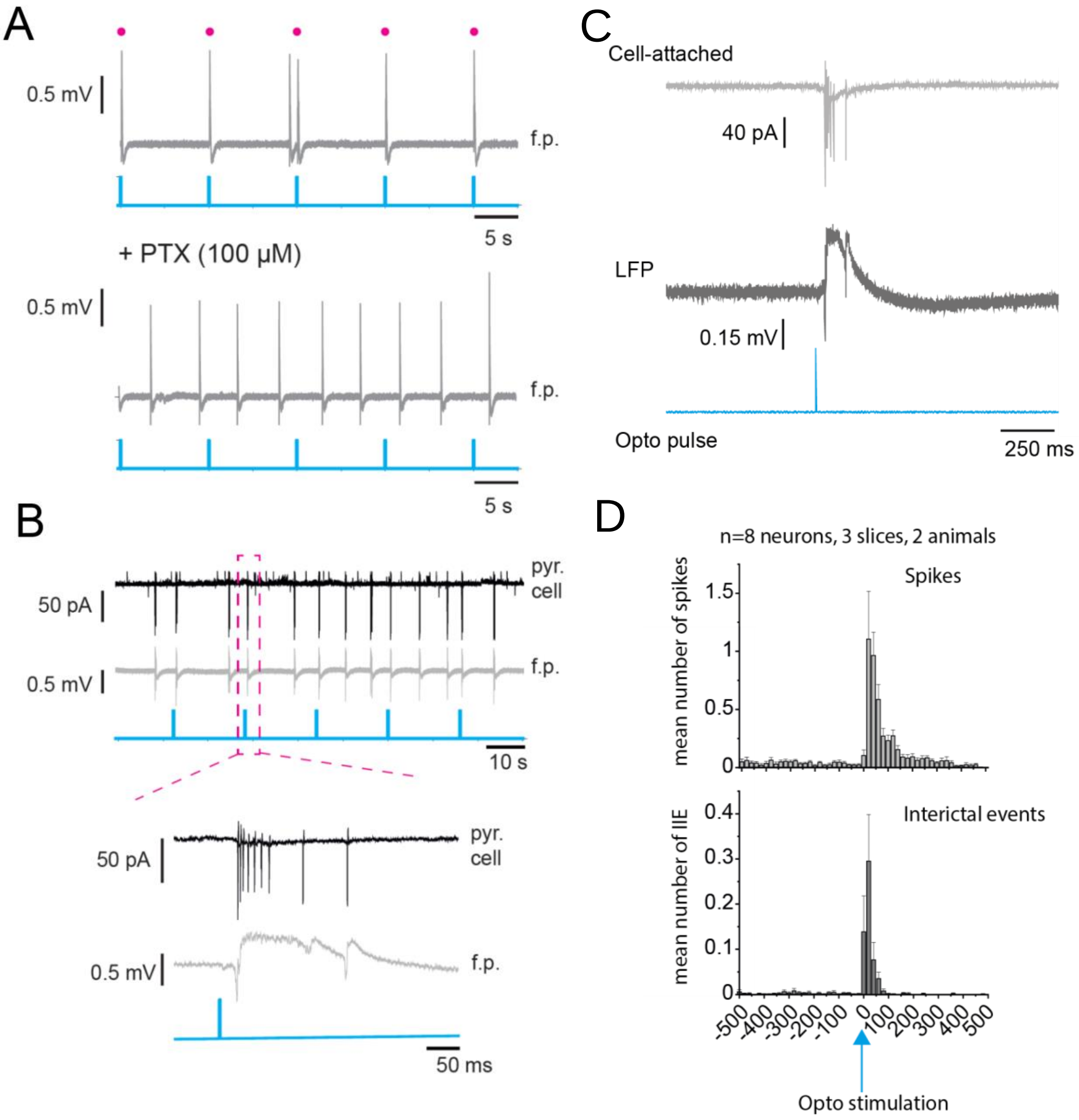

Figure S5. Light-evoked interictal discharges are GABA$_A$R-mediated engaging principal cells, Related to Figure 5.

(A) Example of local field potential (grey trace) recorded in area CA1 (5 mM K^+, 0 mM Mg^{2+}) during optogenetic activation of FS PV+ interneurons (blue). Optogenetic stimulation (1ms pulses) reliably evokes interictal events with intact GABA$_A$Rs (top panel, magenta dots), but not in the presence of picrotoxin (PTX; bottom panel).

(B) Concurrent cell-attached recording of a pyramidal neuron with the local field potential in CA1 region during interictal events.

(C) A characteristic example showing expanded traces of cell-attached current and LFP traces, as indicated.

(D) Spike number histograms; zero, onset of the optogenetic stimulus (n = 8 cells; bin, 20ms); the corrected lag time between optogenetic stimulation and IIE or spikes is 31 ± 6 ms for IIE and 46 ± 4 ms (mean ± SEM) for spikes.

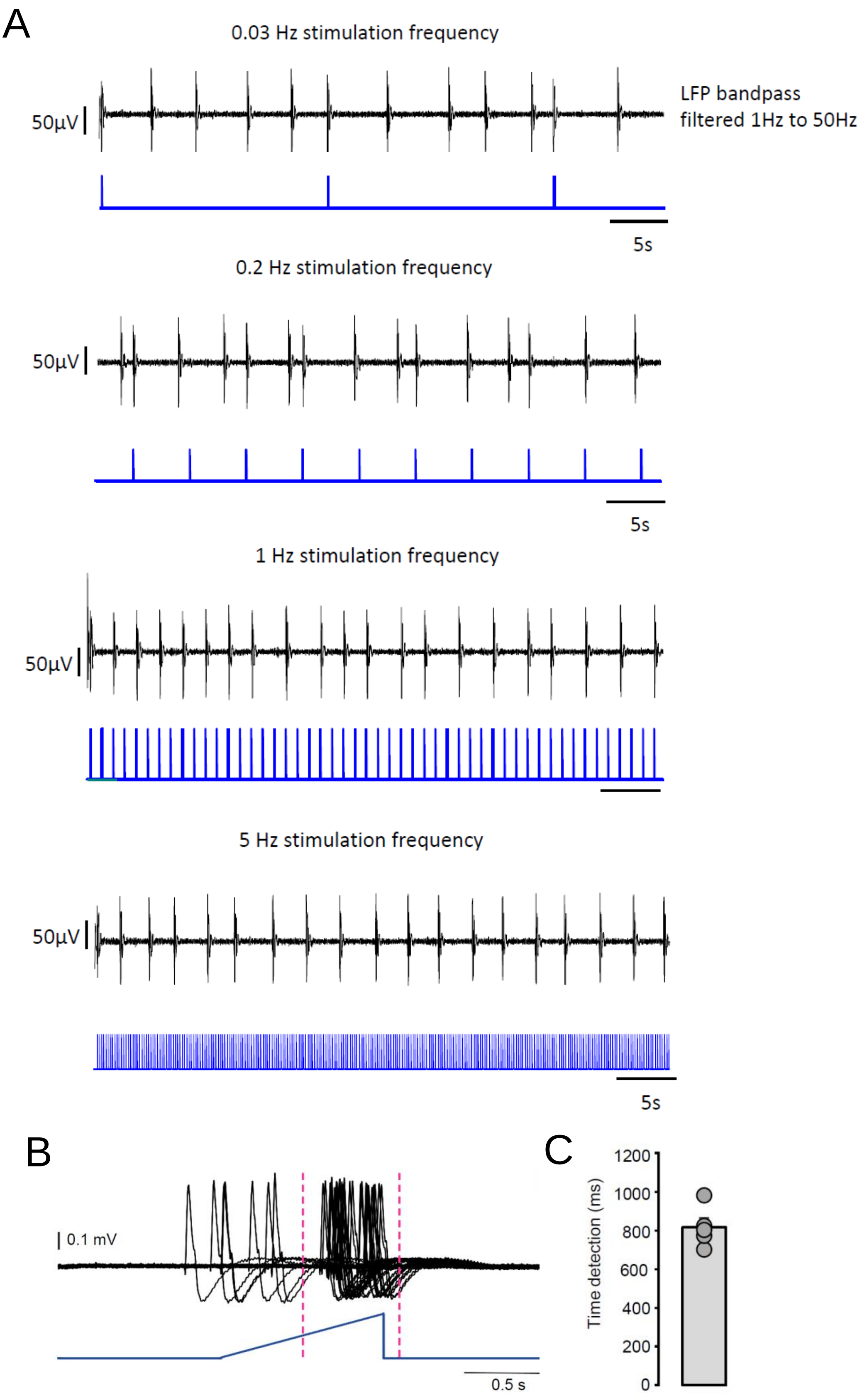

Figure S6. Recording IIEs at varied optogenetic stimulation regimes, Related to Figure 5 and Figure 6.

(A) IIEs (top traces, black; bandpass filter 1-50 Hz) recorded at varied stimulation frequencies (bottom traces, blue; individual 1 ms pulses at 470 nm wavelength).

(B) Example, superimposition of the ramped light stimulus and interictal discharges; dotted lines, 500 ms after the ramp onset, and 100 ms after the ramp endpoint.

(C) Average timepoint of the interictal spike after the ramp onset (bar, mean ± SEM; dots, individual experiments; n = 5 slices in 4 animals).

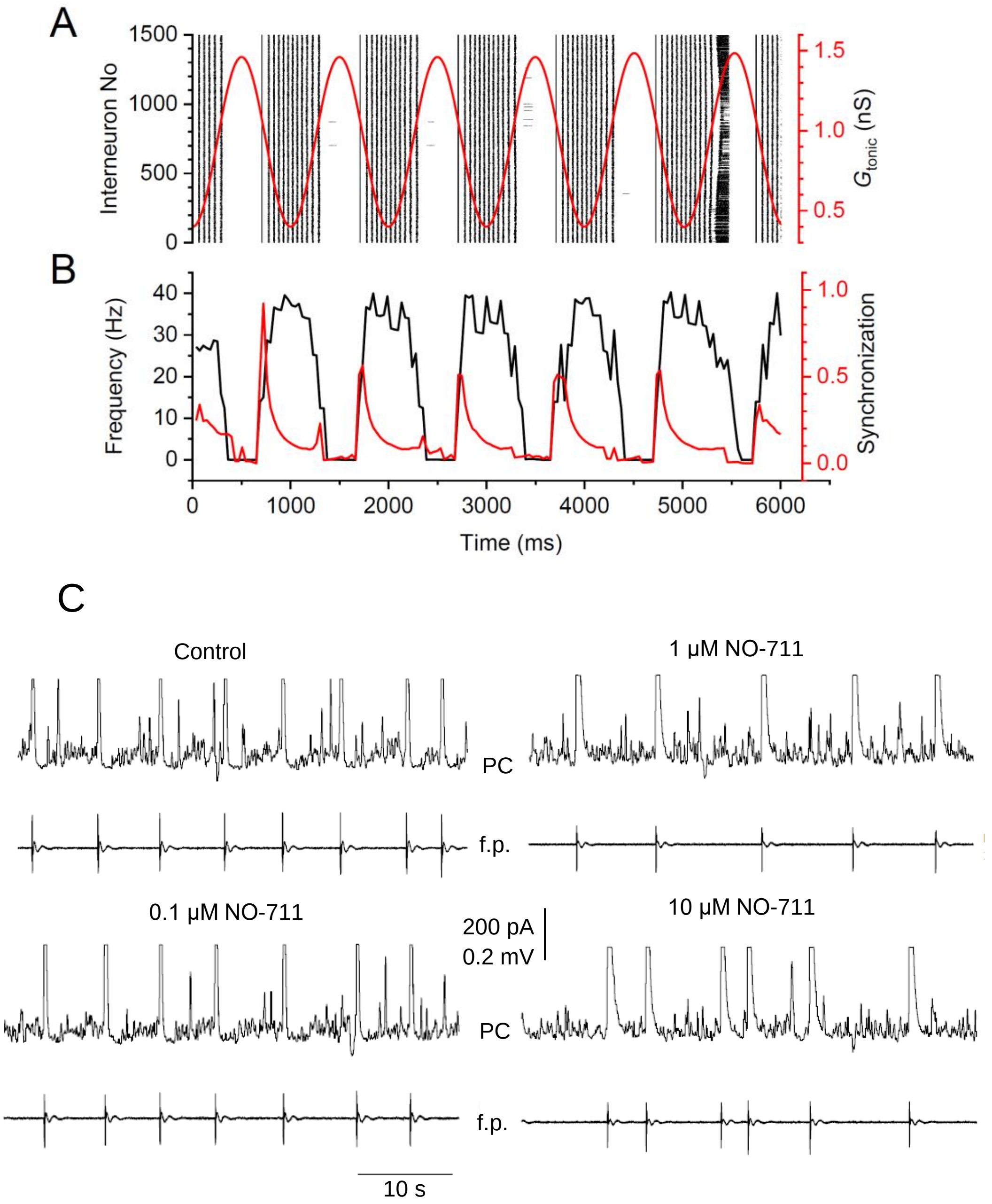

Figure S7. Imposing cycling changes in G_{tonic} or partial blockade of GABA uptake paces rhythmic network activity, Related to Figure 6 and Figure 7.

(A) Raster plot of cell spiking (black) under 1 Hz sin wave of G_{tonic} (red), as indicated.

(B) Average firing frequency and synchronization parameter for network activity shown in A. Key model parameters: cell number N = 1500, intra-network peak synaptic conductance G_{ii} = 0.096 nS cm^{-2}; E-currents (Poisson series) with average synaptic conductance g_s = 0.05 nS, decay constant tau = 3 ms, and frequency f_s = 20 Hz; G_{tonic} reverse potential V_{GABA} = –53 mV, GABA release factor A_f = 10^{-8} nS cm^{-2} ms^{-1}, G_{pump} =0.004 s^{-1} (see Methods for further detail).

(C) Example, simultaneous recording of CA1 pyramidal cell GABAergic currents (top traces; voltage clamp at +10 mV; 30 Hz lowpass filter) and local field potentials (bottom traces, 1-30 Hz bandpass filter), at varied concentrations of NO-711, as indicated; scales as indicated apply throughout the panels.
