# Supplementary material for: Volume-transmitted GABA waves pace epileptiform rhythms in the hippocampal network
Source: Curr Biol. 2023 Apr 10;33(7):1249–1264.e7. doi: 10.1016/j.cub.2023.02.051 (PMC10615848; doi:10.1016/j.cub.2023.02.051)
Supplement: Document S2. Article plus supplemental information [file mmc2.pdf]

Article

Current Biology

Volume-transmitted GABA waves pace epileptiform rhythms in the hippocampal network

Highlights

- GABA sniffer and optical sensor report $[GABA]_e$ rises before interictal events
- Interneuronal network models relate rhythmicity to volume-transmitted GABA actions
- Multi-cell recordings support the concept of periodical GABA-driven disinhibition
- GABA transporters tune the rhythms of interictal events

Authors

Vincent Magloire, Leonid P. Savtchenko, Thomas P. Jensen, ..., Ilya Kolb, Ivan Pavlov, Dmitri A. Rusakov

Correspondence

v.magloire@ucl.ac.uk (V.M.), leonid.savtchenko@ucl.ac.uk (L.P.S.), d.rusakov@ucl.ac.uk (D.A.R.)

In brief

Magloire et al. show that periodic epileptiform discharges are preceded by transient waves of extracellular GABA, which appear critical for the timing of such brain network rhythms.

Magloire et al., 2023, Current Biology *33*, 1249–1264
April 10, 2023 © 2023 The Author(s). Published by Elsevier Inc.
https://doi.org/10.1016/j.cub.2023.02.051

CellPress

Current Biology

CellPress
OPEN ACCESS

Article

Volume-transmitted GABA waves pace epileptiform rhythms in the hippocampal network

Vincent Magloire,[1,6,*] Leonid P. Savtchenko,[1,6,*] Thomas P. Jensen,[1,6] Sergyi Sylantyev,[1,2] Olga Kopach,[1] Nicholas Cole,[1] Olga Tyurikova,[1] Dimitri M. Kullmann,[1] Matthew C. Walker,[1] Jonathan S. Marvin,[3] Loren L. Looger,[3,4,5] Jeremy P. Hasseman,[3,5] Ilya Kolb,[3,5] Ivan Pavlov,[1] and Dmitri A. Rusakov[1,7,*]
[1]UCL Queen Square Institute of Neurology, University College London, Queen Square, London WC1N 3BG, UK
[2]Rowett Institute, University of Aberdeen, Ashgrove Road West, Aberdeen AB25 2ZD, UK
[3]Janelia Research Campus, Howard Hughes Medical Institute, Ashburn, VA, USA
[4]Howard Hughes Medical Institute, University of California, San Diego, La Jolla, CA 92093, USA
[5]GENIE Project Team, Janelia Research Campus, Howard Hughes Medical Institute, Ashburn, VA, USA
[6]These authors contributed equally
[7]Lead contact
*Correspondence: v.magloire@ucl.ac.uk (V.M.), leonid.savtchenko@ucl.ac.uk (L.P.S.), d.rusakov@ucl.ac.uk (D.A.R.)
https://doi.org/10.1016/j.cub.2023.02.051

SUMMARY

Mechanisms that entrain and pace rhythmic epileptiform discharges remain debated. Traditionally, the quest to understand them has focused on interneuronal networks driven by synaptic GABAergic connections. However, synchronized interneuronal discharges could also trigger the transient elevations of extracellular GABA across the tissue volume, thus raising tonic conductance (G_{tonic}) of synaptic and extrasynaptic GABA receptors in multiple cells. Here, we monitor extracellular GABA in hippocampal slices using patch-clamp GABA "sniffer" and a novel optical GABA sensor, showing that periodic epileptiform discharges are preceded by transient, region-wide waves of extracellular GABA. Neural network simulations that incorporate volume-transmitted GABA signals point to a cycle of GABA-driven network inhibition and disinhibition underpinning this relationship. We test and validate this hypothesis using simultaneous patch-clamp recordings from multiple neurons and selective optogenetic stimulation of fast-spiking interneurons. Critically, reducing GABA uptake in order to decelerate extracellular GABA fluctuations—without affecting synaptic GABAergic transmission or resting GABA levels—slows down rhythmic activity. Our findings thus unveil a key role of extrasynaptic, volume-transmitted GABA in pacing regenerative rhythmic activity in brain networks.

INTRODUCTION

GABA is the principal inhibitory neurotransmitter in the brain. It mediates conventional fast inhibitory synaptic transmission through ionotropic Cl^--permeable $GABA_A$ receptors ($GABA_A$Rs), as well as slower transmission mediated by $GABA_B$ receptors. However, in certain conditions, such as postsynaptic cell depolarization or high intracellular chloride concentration ([Cl^-]), $GABA_A$R current can become excitatory.[1] In addition, there is a slow $GABA_A$R-mediated conductance (G_{tonic}), often referred to as "tonic inhibition," which reflects recurrent receptor activation by GABA present throughout the extracellular space. Historically, microdialysis studies *in vivo* have suggested that the ambient extracellular GABA concentration ($[GABA]_e$) is relatively stable, although it may change with physiological state.[2] Indeed, long-lasting changes in average $[GABA]_e$ should be constrained by GABA transporters.[3,4] However, the dialysis technique usually reports $[GABA]_e$ averaged over many seconds, whereas the affinity of the main GABA transporter GAT-1 (K_m = 10–20 μM)[5–7] is too low to prevent faster, low-micromolar-range fluctuations in $[GABA]_e$.

The dynamics of G_{tonic} have been associated with the firing of interneurons[8] and are implicated in epileptiform discharge initiation.[4,9] Changes in G_{tonic} can also alter the excitability of principal cells (PCs),[10–13] influencing their network behavior,[14,15] including basic rules of coincidence detection.[16–18] While a collapse in inhibitory activity has been associated with epileptiform events,[19] interneurons appear to maintain or increase their firing rate before the onset of ictal activity,[20,21] thus implying a role in the generation of ictal events.[22,23] In addition, seizure-like events in pyramidal neurons can be preceded by fast-spiking (FS) neuronal firing,[24] leading to an outward $GABA_A$R current and a rise in extracellular K^+.[23] Optogenetic activation of GABAergic cells can paradoxically engage both ictal and interictal discharges in both *ex vivo* and *in vivo* acute models of epilepsy[25–29] (reviewed in Magloire et al.[30]). However, when monitored in bulk, the majority of interneurons do not increase their activity during spontaneous seizures *in vivo*,[31] reflecting the heterogeneous nature of their involvement.[32–34] While these observations implicate complex dynamic interactions between network inhibition and excitation in the generation of interictal and ictal discharges, how these interactions regulate epileptiform rhythms remains poorly understood.

Intriguingly, we have previously found a bell-shaped relationship between G_{tonic} (controlled through either dynamic clamp or by changing ambient $[GABA]_e$), and the firing and synchronization of interneuronal networks.[12,15] Because G_{tonic} itself depends on interneuronal firing, this type of relationship suggests,

This is an open access article under the CC BY license (http://creativecommons.org/licenses/by/4.0/).

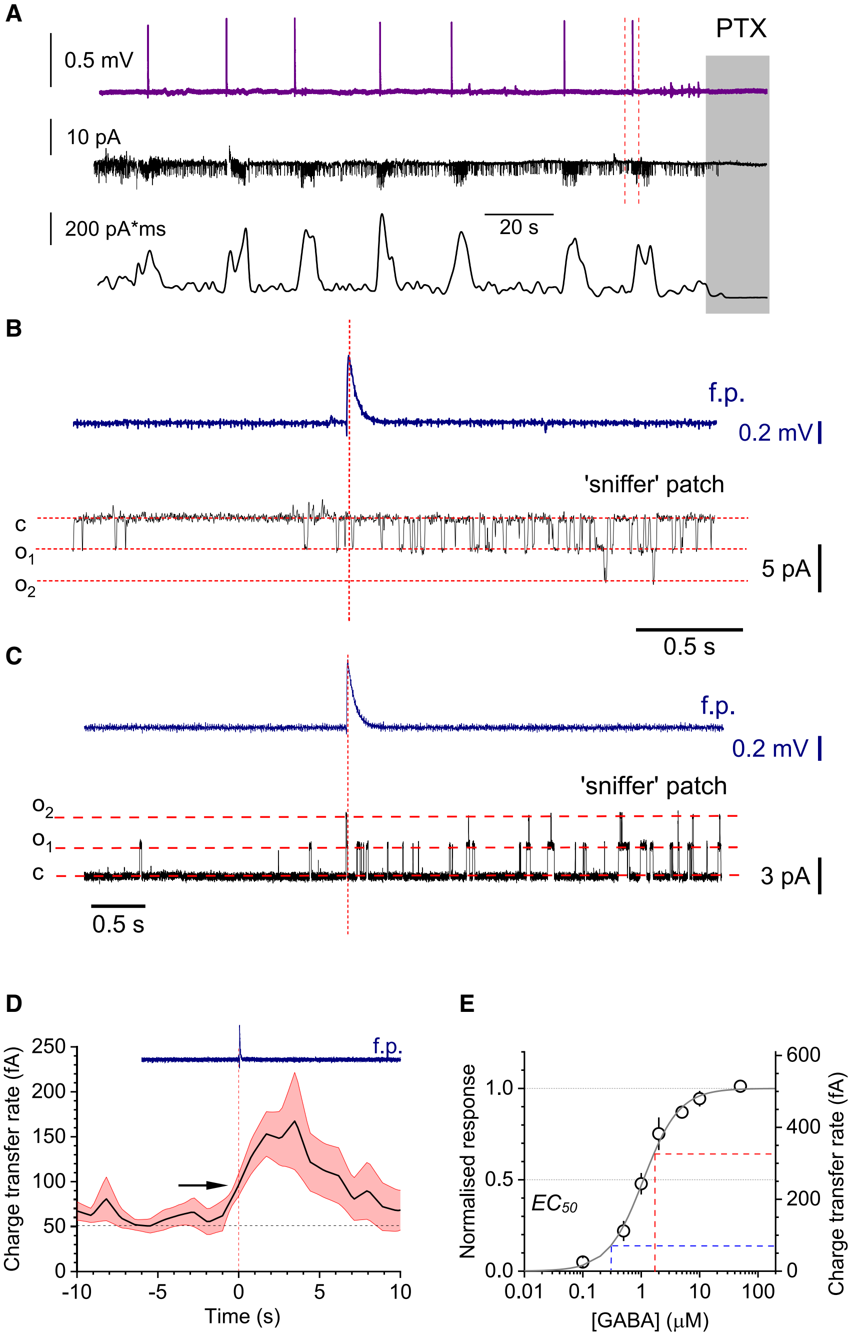

Figure 1. Rhythmic fluctuations of extracellular GABA during interictal activity in the hippocampal area CA1

(A) Local field potentials (LFPs, top trace), sniffer-patch single-channel activity (middle), and charge dynamics (bottom) during interictal discharges in hippocampal slices. Gray shade, picrotoxin (PTX) application at the end of trials. See also Figure S1A.

(B) Fragment from (A) (between dotted lines), expanded to show the LFP (top, blue) and patch recording (bottom, black) details. High-chloride pipette solution was used to record inward $GABA_AR$-mediated channel openings (V_m = −70 mV).

(C) Similar setting to (B), but with low chloride solution to document outward currents (V_m = 0 mV).

(D) Averaged time course of charge transfer rate (equal to current; mean ± SEM for slice-average traces, n = 6 slices) at the onset of individual field potential (f.p.) IIE (vertical dashed line, blue trace). Arrow, interictal discharge onset coincides with $GABA_AR$ activity reaching ~35% of its peak.

(E) Normalized and absolute charge transfer values recorded in sniffer patches at different $[GABA]_e$ (dots, mean ± SEM; n = 4–6 for individual values; gray line, sigmoid best fit). Dashed lines, $[GABA]_e$ during resting level (blue) and peak (red) of channel activity recorded. See Figures S1B–S1G for field-potential and single-cell recordings.

at least in theory, an inherent capacity for self-organizing rhythms or periodic waves of activity.[35] Genetically encoded optical GABA sensors have reported prominent $[GABA]_e$ transients (10- to 100-ms timescale) during interictal spikes *in vivo*,[36] arguing that fluctuations in G_{tonic} may have a role in periodic network activity in the intact brain.

We therefore hypothesized that the synchronized firing of hippocampal interneurons should drive transient, volume-transmitted changes in $[GABA]_e$, which could in turn control G_{tonic} and thus regulate rhythmic network activity such as interictal events (IIEs). We tested this hypothesis by combining outside-out membrane patch (GABA sniffer) recordings,[37,38] the novel optical GABA sensor iGABASnFR2,[39] an improved version of iGABASnFR,[36] and optogenetic stimulation of FS, parvalbumin-positive (PV+) interneurons, with computer simulations of spiking neural networks.[40,41] We also asked whether reducing GABA uptake, which should curb extracellular GABA waves but not synaptic GABAergic transmission or ambient $[GABA]_e$, would alter the rhythm of IIEs.

RESULTS

Transient $[GABA]_e$ rises tend to precede IIEs

First, we elicited epileptiform activity in acute hippocampal slices perfused with a 0 $[Mg^{2+}]$/5 mM $[K^+]$ solution; in the CA1 region, this activity generated brief (<200 ms) periodic field-potential spikes reflecting IIEs[42,43] (Figure 1A, top trace). To monitor the $[GABA]_e$ dynamics in these settings, we used an outside-out "sniffer" patch, which reports $GABA_AR$ channel activity,[37] as previously described.[18,38] Channel opening frequency appeared synchronized with IIEs (Figure 1A, middle), yet a detailed analysis revealed that it always started to increase 0.5–1 s before IIEs (Figures 1B, 1C, and S1A; summary in Figure 1D). We took special care to prepare uniformly shaped sniffer patch pipettes for these experiments, and used a multi-channel rapid solution exchange to calibrate $[GABA]_e$

CellPress
OPEN ACCESS

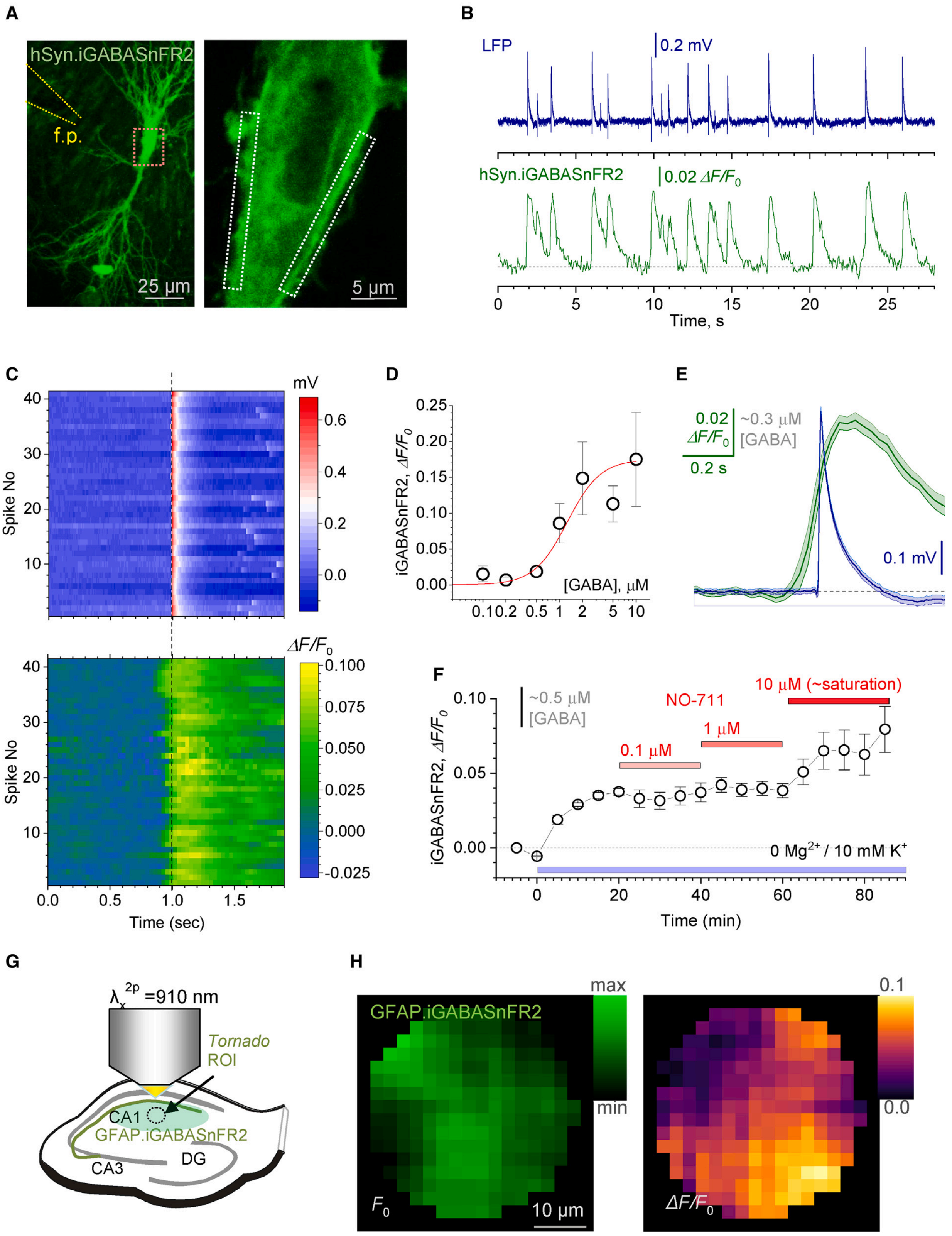

Figure 2. Optical registration of $[GABA]_e$ dynamics using iGABASnFR2 expressed in hippocampal slices

(A) CA1 pyramidal cell expressing iGABASnFR2 with a field pipette electrode (f.p., left) and two imaging ROIs near the cell soma periphery (right, zoomed-in fragment shown by red rectangle on the left; λ_x^{2p} = 910 nm).

(legend continued on next page)

against $GABA_AR$ channel activity.[44] We thus estimated an EC_{50} value of ~1.1 μM, which corresponds to a charge transfer rate (or current) of 256 ± 57 $fC \cdot s^{-1}$ (fA; mean ± SEM here and below unless shown otherwise; n = 6; Figure 1E; note that the average of individual peak values differs from the peak value of the average charge transfer trace centered at the IIE onset). Thus, we estimated that $[GABA]_e$ rises from its resting level of ~0.3 μM to a peak of 1.5–2 μM during IIEs (Figure 1E).

We next explored the cellular basis of these observations. The spiking activity of individual FS PV+ interneurons increased substantially from their basal level several seconds before the peak of CA1 pyramidal cell bursts that represent IIEs (Figures S1B and S1C). This was faithfully reflected by the elevated GABAergic input to CA1 pyramidal cells before the IIEs (Figures S1D and S1E). In contrast, pyramidal cell spiking appeared time-locked to the epileptiform discharges (Figures S1F and S1G). These observations reflect earlier findings that interictal bursting is preceded by an increase in firing of FS PV+ interneurons,[19,23,29,45] which we suggest represents, at least partly, a "global" wave of $[GABA]_e$ detected with the sniffer patch (Figure 1).

Interictal spikes are preceded by a $[GABA]_e$ elevation landscape across tissue

Although the sniffer patch can detect $[GABA]_e$ changes with high sensitivity, it reports average $[GABA]_e$ near the slice surface, potentially far away from the GABA release sites—and at any rate, averaged over many cells and environments. To understand whether $[GABA]_e$ displays similar dynamics within the pyramidal cell layer (a key target area of FS interneurons), deep within the tissue, we used the recently developed optical GABA sensor iGABASnFR2,[39] a novel version of iGABASnFR.[36] First, we expressed the sensor in a proportion of CA1 and CA3 pyramidal cells under the *hSynapsin-1* promoter, in organotypic hippocampal slices (Figure 2A): sparse expression of iGABASnFR2 should reduce the overall extracellular presence of GABA-binding sites and therefore minimize any potential buffering effects on the $[GABA]_e$ dynamics, as demonstrated earlier for glutamate and its sensors.[46,47]

We imaged iGABASnFR2 fluorescence within microscopic regions of interest (ROIs) near the periphery of individual pyramidal cell somata using two-photon excitation microscopy, as described earlier,[48,49] while recording field potentials nearby (Figure 2A). Periodic IIEs were consistently accompanied by transient elevations of iGABASnFR2 fluorescence that, again, started to rise before individual IIEs (Figures 2B, 2C, and S2A): the $[GABA]_e$ rise onset was consistently 80–90 ms prior to IIE onset. Although the inter-spike interval in this preparation (2–4 s) was shorter than that in acute slices (15–20 s; Figure 1A), the recovery of $[GABA]_e$ to its resting (equilibrated) level took a similar fraction of the periodic cycle in both preparations. In the context of network dynamics (see below), this correspondence is consistent with the role of the GABA wave time course in pacing the network rhythm.

To translate our imaging data into (approximate) $[GABA]_e$, we recorded iGABASnFR2 fluorescence against increasing [GABA] applied externally, in isolated acute hippocampal slice preparations. The resulting dependence (Figure 2D) suggested that, during IIEs, the peak iGABASnFR2 signal of 0.074 ± 0.006 $\Delta F/F_0$ (mean ± 95% CI) corresponds to an ~1.1-μM increase in $[GABA]_e$ (Figure 2E). This estimate is in excellent agreement with the sniffer patch data (Figure 1E).

In such experiments, high $[K^+]_{out}$/low $[Mg^{2+}]$ ("epileptogenic artificial cerebrospinal fluid [aCSF]") boosts cell excitability, prompting generation of IIEs. To understand whether this manipulation also increases ambient $[GABA]_e$, we monitored iGABASnFR2 fluorescence in normal aCSF, after introducing epileptogenic aCSF, and under the partial blockade of the main GABA transporter GAT-1, using increasing concentrations of its blocker NO-711 (Figure 2F). The results suggest that introducing an epileptogenic solution raises $[GABA]_e$ by 0.7–0.8 μM, and that the partial blockade of GAT-1 does not change this new equilibrium until the blockade reaches a saturation level (Figure 2F). We also asked whether fluctuations in $[K^+]_{out}$ could be involved in pacing IIEs, but our electrophysiological recordings argued against it, in present conditions, by indicating no K^+-driven depolarizing shifts before IIEs (Figure S2B).

Finally, we asked whether $[GABA]_e$ elevations were homogeneous across the neuropil. To address this, we used acute slices with iGABASnFR2 expressed in astroglia (Figure 2G), which provided nearly contiguous territories of expression (Figure S2C), so that we could image GABA release landscapes during IIEs. These experiments also require high temporal resolution, such as millisecond-range *Tornado*-scanning, demonstrated before,[48] to avoid rapid spatial equilibration of the diffusing GABA signal. A characteristic snapshot of the iGABASnFR2 signal during an IIE (30 ms $\Delta F/F_0$ window) illustrates the

(B) One-slice example, fEPSPs (top) and iGABASnFR2 signal (bottom) recorded during interictal spikes. Dotted line, apparent baseline reflecting the resting/equilibrated $[GABA]_e$. See Figure S2A for further detail.

(C) Diagram showing 41 interictal spikes aligned with respect to their peak times (top), and the corresponding iGABASnFR2 signals (bottom; false color scale). See Figure S2B for details of field potential recordings.

(D) iGABASnFR2 fluorescence increment against increasing $[GABA]_e$ in acute hippocampal slice preparation (mean ± SEM; n = 7 slices). Line, best-fit sigmoidal Hill function at $E = 0.175$; $K_d = 1.26$ μM, n = 2 (STAR Methods).

(E) Summary of LFP and iGABASnFR2 recordings shown in (B) and (C), aligned in time, as indicated: traces and shaded area, mean ± 95% confidence interval (n = 41).

(F) Average iGABASnFR2 signal (mean ± SEM, n = 3 hippocampal slices, 5–10 ROIs each) monitored in slices in control conditions, under epileptiform conditions (10 mM $[K^+]_{out}$), and under partial GAT-1 transporter blockade achieved by using sub-saturation concentrations of the blocker NO-711, as indicated. Approximate scale bar for [GABA] in (E) and (F) is derived from data in (D).

(G) Experimental diagram: imaging IIE-driven GABA release landscape in CA1 neuropil with high spatiotemporal resolution using astroglia-expressed iGABASnFR2. See Figure S2C for further illustration.

(H) Fluorescence landscape of iGABASnFR2 (15 spiral-scans/30 ms average) in baseline conditions (before an IIE; left) and the $\Delta F/F_0$ GABA signal landscape immediately after an IIE (right). Average for four consecutive IIEs.

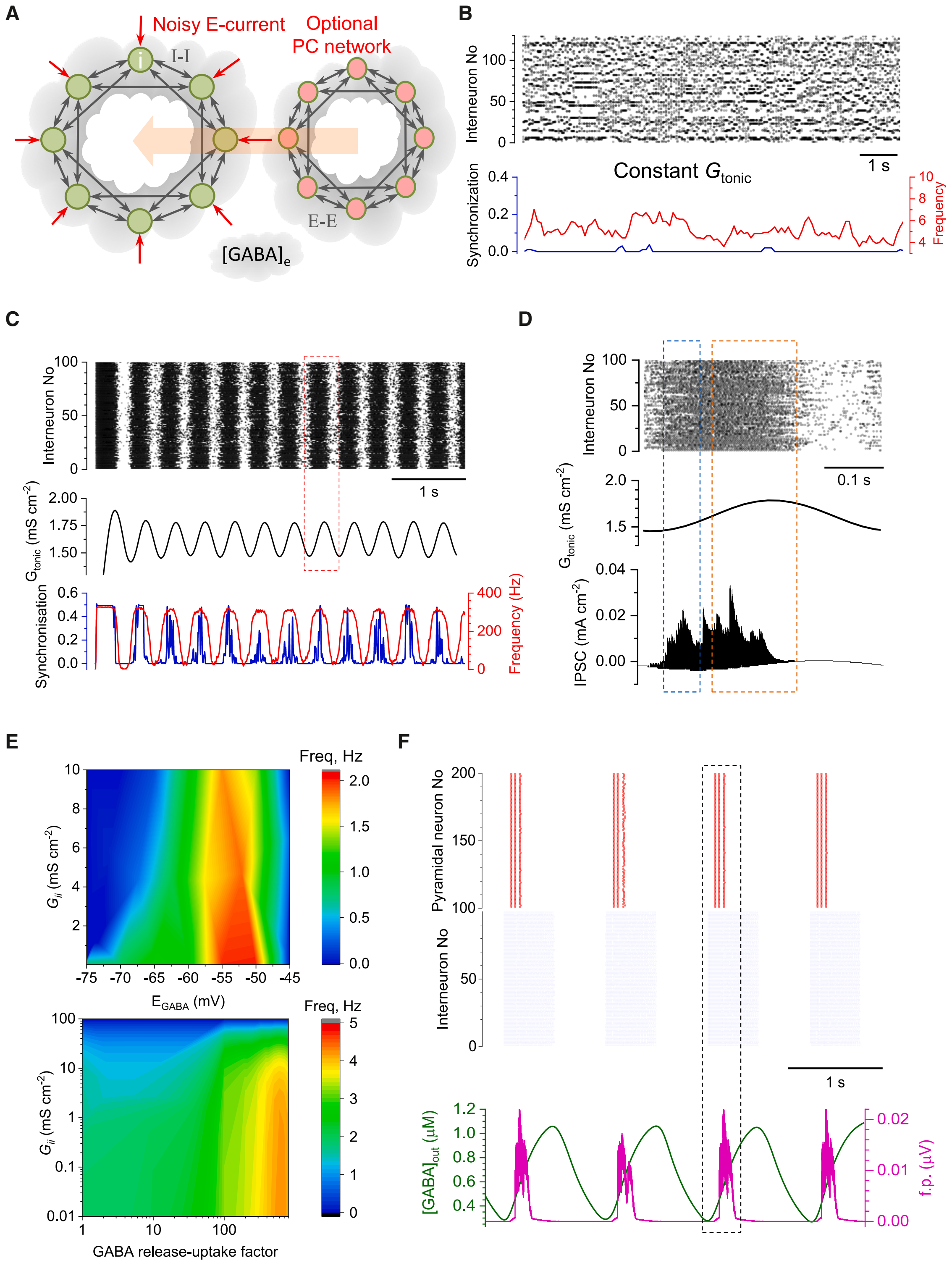

Figure 3. $[GABA]_e$-dependent G_{tonic} steers rhythmic activity of a modeled interneuron network across a range of parameters

(A) Schematic. FS interneuron network model (*I-I*, GABAergic synaptic connections), with external excitatory inputs (*E*-current); volume-transmitted $[GABA]_e$ signal generating G_{tonic}; and an optional network of pyramidal cells incorporated (*E-E*, excitatory connections).

(B) Raster plot of interneuronal network spiking (top), network synchronization coefficient (bottom, blue), and time course of average spiking frequency (bottom, red) for simulated network with constant G_{tonic} value (0.44 mS cm^{-2}).

(C) Raster plot as in (B), but with G_{tonic} driven by $[GABA]_e$ (middle) calculated from integrated interneuronal discharges; network synchrony and mean network frequency show clear periodicity (bottom). Key model parameters: cell number N = 100, intra-network peak synaptic conductance G_{ii} = 0.1 mS cm^{-2}; *E*-currents

(legend continued on next page)

heterogeneous landscape of GABA release (Figure 2H), which should reflect uneven distribution of active GABAergic axons.

Biophysical basis of $[GABA]_e$-dependent network rhythms

We next explored the mechanistic relationship between $[GABA]_e$ fluctuations and IIE rhythms, by expanding our earlier models[15,40,41] to simulate a network of FS hippocampal interneurons equipped with stochastic excitatory inputs (Figure 3A). While previous modeling studies have proposed a plausible network machinery of high-frequency rhythms, including sharp-wave ripples,[50–52] they did not consider mechanisms relevant to IIE generation: 0.2- to 1.0-Hz frequency range or the potential role of GABA waves. One theoretical study neatly suggested extracellular K^+ waves as the volume-transmitted feedback driving 2- to 5-Hz network oscillations,[53] but such $[K^+]_{out}$ fluctuations could also regulate glutamate and GABA uptake. Here, we postulated that interneuronal discharges generated both IPSCs and volume-transmitted $[GABA]_e$ rises (Figure 3A) that contributed to G_{tonic} in multiple cells.[40,41] G_{tonic} was thus set as a monotonic function of $[GABA]_e$, which in turn was determined by (1) a scalable $[GABA]_e$ increment upon each individual synaptic discharge and (2) GABA uptake by GAT-1 providing an exponential $[GABA]_e$ decay[6,54] (STAR Methods).

We simulated networks of varied sizes, over a plausible range of synaptic GABA release intensities and GABA uptake rates. It was clear that under constant $[GABA]_e$ (hence G_{tonic}), cells would fire stochastically at a near-constant average frequency with little synchronization (Figure 3B). In striking contrast, when $[GABA]_e$ is driven by interneuronal spiking, the network slips into periodic activity (Figures 3C and 3D) over a wide range of frequencies, depending on the excitatory drive (*E*-current), synaptic connectivity strength (G_{ii}), and the GABA-release-uptake scaling factor *R* (Figure 3E; STAR Methods). There was a robust relationship between interneuronal spiking, synchronization parameter *k* (STAR Methods), $[GABA]_e$, and G_{tonic} during periodic bursts of activity (Figures 3C–3E and S3). Interestingly, larger networks (containing 400–1,500 cells) tended to have a more regular activity cycle (Figure S4), consistent with classical theory predicting less variability near a system's Hopf bifurcation point,[55] which occurs toward the peak of G_{tonic}.

Finally, we explored the relationship between cell spiking activity, G_{tonic}, and $[GABA]_e$ in a twinned network configuration incorporating both interneurons and PCs equipped with $GABA_A$Rs (Figure 3A). First, such networks turned out to show stable behavior under external stimuli when connectivity between PCs and interneurons was ~100 times weaker than PC-PC or interneuron-interneuron connectivity. We next found that the PC network activity was highly sensitive to G_{tonic} generated by the interneuronal network, over a wide range of stochastic excitatory input. PCs showed brief, highly synchronized bursts of activity that followed periodic rises of interneuronal spiking (Figure 3F). Strikingly, the PC spiking bursts occurred on the ascending phase of $[GABA]_e$ waves (Figure 3F), thus displaying some distinct features of the epileptiform events that we observed experimentally (Figures 1D, 2B, 2C, and 2E).

Self-maintained waves of tonic $GABA_A$ conductance in hippocampal slices

To test whether the relationships suggested by the network models were valid, we carried out several experiments. First, we asked whether the dynamics of G_{tonic} predicted by simulations (Figures 3A–3D) occur in hippocampal circuits in the absence of glutamatergic transmission. We therefore recorded $GABA_A$R-mediated whole-cell currents from CA1 pyramidal neurons in the presence of AMPA, NMDA, and $GABA_B$ receptor blockers.

Elevating $[K^+]_{out}$ to 10 mM (in 0 Mg^{2+}) elicited an outward shift in the holding current, which was sensitive to the non-competitive $GABA_A$R channel blocker picrotoxin (PTX) (Figures 4A and 4B) and consisted of brief transients riding on periodic low-frequency waves (Figure 4C). The frequency of slow $GABA_A$ R-mediated waves (0.2–0.3 Hz) was similar to the periodic $[GABA]_e$ transients observed during GABA imaging experiments (Figure 2B). Consistent with the network model (Figures 3A–3D, S3, and S4), fast $GABA_A$R-mediated currents, which most likely reflect individual interneuron discharges, tended to increase in intensity prior to the peak of the slow G_{tonic} transients reflecting $[GABA]_e$ waves (Figures 4C–4E).

Synchronization of FS PV+ interneurons during GABA waves

To understand the dynamics of synchronized interneuronal activity during GABA waves, we carried out triple-cell recordings monitoring pairs of FS PV+ interneurons (cell-attached) and a CA1 pyramidal cell (whole cell), with ionotropic glutamate receptors blocked (Figure 4F). Clear rhythmicity of interneuron activity was documented in three out of nine experiments: wave-like activity (STAR Methods) occurred in both cell-attached FS PV+ interneurons (Figure 4G), suggesting that ~35% of FS PV+ interneurons could steer epileptiform discharges. This appeared in good correspondence with network simulations predicting that

(Poisson series) with average synaptic conductance $g_s = 0.02$ mS cm^{-2}, decay constant tau = 3 ms, and frequency $f_s = 100$ Hz; $GABA_A$R reversal potential $V_{GABA} = -56$ mV, GABA release factor $A_f = 1.35 \times 10^{-7}$ nS cm^{-2} ms^{-1}, $G_{pump} = 0.003$ ms^{-1} (STAR Methods).

(D) Fragment from (C) (red dotted rectangle) enlarged, with the integrated IPSC time course (bottom). Blue and orange dotted rectangles indicate high-frequency, non-synchronized, and oscillating and synchronized IPSC periods, respectively.

(E) Parameter-space heatmaps: rhythm generation over a range of G_{ii} and E_{GABA} ($R = 1$, top), and G_{ii} and release-uptake factor *R* (STAR Methods; $E_{GABA} = -65$ mV, bottom). Deep blue area indicates no detectable rhythmic activity.

(F) Top: raster plot for a twinned network (red, pyramidal neurons; blue, interneurons), with weak internetwork (*I-E* and *E-I*) and strong intra-network (*I-I* and *E-E*) connections. Bottom: time course of field potential (f.p.; simulated for pyramidal neurons at 250 μm from the network, tissue conductance of 100 mS cm^{-1}; STAR Methods) and $[GABA]_e$, as indicated. Key model parameters: N = 200 (100 pyramidal cells and 100 interneurons), $G_{ii} = 0.216$ mS cm^{-2}, $G_{ee} = 0.003$ mS cm^{-2}; internetwork peak synaptic conductance $G_{ei} = 0.00012$ mS cm^{-2} and $G_{ie} = 0.00064$ mS cm^{-2}, *E*-currents (Poisson series) $g_{si} = 0.3$ mS cm^{-2}, tau = 3 ms, $f_s = 20$ Hz; *I*-current (Poisson series) $g_{se} = 0.003$ mS cm^{-2}, decay constant tau = 3 ms, $f_s = 20$ Hz, $V_{GABA} = -58$ mV, $A_f = 2 \times 10^{-7}$ nS cm^{-2} ms^{-1}, $G_{pump} = 0.003$ ms^{-1}. See Figures S3 and S4 for further exploration of the model parameter space.

CellPress
OPEN ACCESS

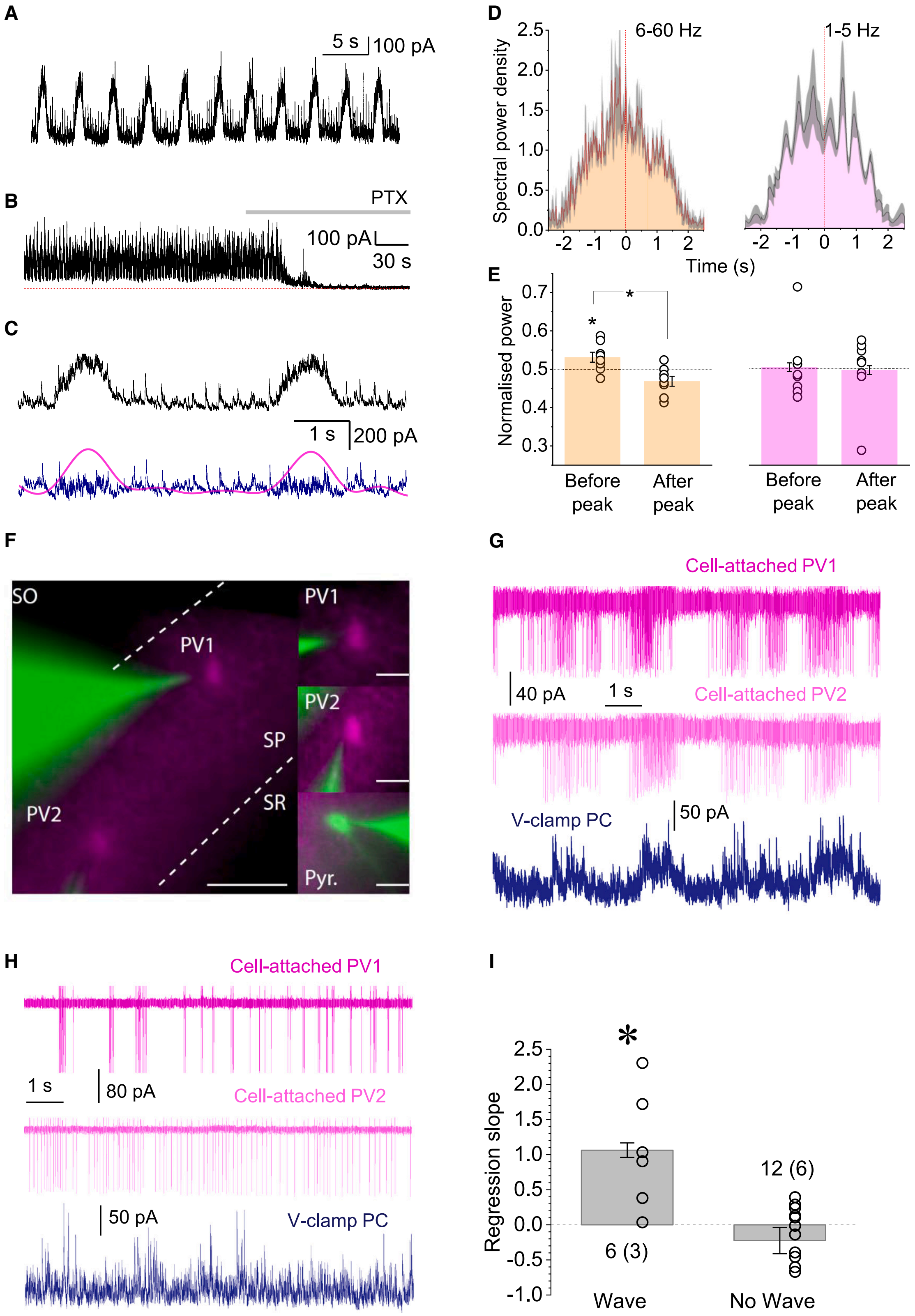

Figure 4. Slow wave-like oscillations and synchronization of FS PV+ interneuron spiking activity during GABA waves

(A) Trace. Example of $GABA_AR$-mediated currents received by a CA1 pyramidal neuron (voltage-clamp with low intracellular Cl^-; V_h = 0 mV).

(B) Slow, wave-like oscillations as in (A) are readily blocked by the $GABA_AR$ antagonist picrotoxin (PTX).

(legend continued on next page)

peak synchronization involves up to 40% of interneurons (Figure 3C). Indeed, over 50% of interneurons in areas CA1/CA3 show no change in firing during spontaneous IIEs *in vivo*.[31]

Here, quasi-periodic bursts of interneuron spiking were observed, together with the corresponding fluctuations of GABAergic currents in the recorded pyramidal neuron (Figure 4G). Overall, experiments that featured rhythmic waves (Figure 4G) also showed a significant correlation between spiking frequency and synchronization of the two PV+ cells, whereas recordings with little rhythmicity (Figure 4H) corresponded to no such correlation, reflecting possibly intense yet non-synchronized firing (Figure 4I). In these experiments, the wave pattern was detected in one out of three cases, although our network simulations suggest its prevalence (Figure 3). This apparent discrepancy is likely to arise because modeled cells have equivalent intrinsic and connectivity properties, whereas real cells and circuits are highly diverse. The latter may explain the reported heterogeneity of interneuronal involvement in IIE generation.[32–34] Indeed, we detected heterogeneous GABA landscapes during IIEs (Figure 2H), arguing that the detection of coordinated rhythms in 1/3 of cases involving randomly selected cells is consistent with our hypothesis.

Spiking of principal neurons is synchronized shortly after the peak of interneuronal firing

Next, to relate interneuronal activity to PC firing, we recorded two neighboring CA1 or CA3 pyramidal cells, one in cell-attached and one in voltage-clamp mode (10 mM $[K^+]_{out}$ ionotropic glutamate receptors intact). Pyramidal cell spiking showed a characteristic lull during the rise of G_{tonic} transients (Figure 5A, left, blue segments), yet resumed spiking near their peak. This "network rebound" phenomenon has been explored previously,[12,15,56] suggesting that the shunting (self-inhibition) of interneuronal networks prompts relatively rapid, network-driven disinhibition of PCs near the peak of interneuronal activity. Blocking $GABA_A$Rs with PTX abolished this periodic fluctuation (Figure 5A right).

Thus, a rise in interneuron activity could paradoxically initiate IIEs by synchronizing PC firing, the phenomenon explored previously.[23] To test this further, we expressed channelrhodopsin-2 (ChR2-H134R-eYFP) in the hippocampal FS PV+ cells (Figure 5B) to enable their synchronous activation using widefield optogenetic stimulation (Figure 5C). We asked whether such stimulation could prompt IIEs in epileptogenic aCSF (5 mM $[K^+]_{out}$, 0 $[Mg^{2+}]$). Indeed, a single 1-ms light pulse evoked large $GABA_A$R IPSCs in CA1 pyramidal neurons, generating coincident epileptiform field potentials that were indistinguishable from spontaneous IIEs (Figures 5D and S5A). Again, this observation was similar to that reported in cortical slices under a different epileptogenic protocol.[29]

Blocking $GABA_A$R with PTX abolished light-evoked IIEs, but not other periodic bursts (Figures 5D and S5A). This revealed the two different types of short epileptiform discharges observed in slices, pre-ictal bursts that are purely glutamatergic, and IIEs that involve both glutamatergic and GABAergic activity.[57,58] Optogenetic stimulation of FS PV+ cells evoked IIEs and spike bursts in pyramidal neurons, with comparable post-stimulus lags (31 ± 6 ms for IIEs and 46 ± 4 ms for pyramidal spikes; Figures S5B–S5D). Thus, an abrupt release of FS PV+ cell inhibition upon light turnoff triggered synchronous PC discharges. Overall, light stimuli applied at 0.03–0.2 Hz evoked IIEs with ~0.8 probability, whereas stimulation at > 0.2 Hz (value close to natural periodicity, 0.14 ± 0.03 Hz) was less effective, with the probability decreasing to ~0.1 at 5–10 Hz (Figures 5E and S6A).

Evoked $[GABA]_e$ waves can entrain network rhythms

To see whether a relatively slow wave of GABA release could entrain IIEs, we light-activated PV+ cells using longer-lasting ramp stimuli (1- to 2-s duration, comparable to spontaneous GABA waves), while recording IPSCs in CA1 pyramidal cells. In normal aCSF, this stimulation elevated GABAergic activity only to a certain point, after which the IPSC frequency rapidly dropped (Figures 6A and 6B), whereas in epileptogenic aCSF it led to IIEs (Figures 6C, 6D, and S6B). Indeed, in five out of eight experiments, we observed wave-like $GABA_A$R-mediated currents induced by optogenetic ramp stimulation, inducing IIEs with a probability of 0.25 ± 0.09 and a delay of 831 ± 47 ms (detection window between 500 and 100 ms post-stimulation; Figure S6C). In contrast, in the three experiments without light-evoked GABA waves, the probability of IIE generation was only 0.06 ± 0.04.

To understand the biophysical basis of this phenomenon, we asked whether, in our simulated network, the G_{tonic} dynamics on their own could pace network synchronization and rhythmicity. We therefore forced G_{tonic} to follow a 1-Hz sine wave and found that cell firing indeed peaked at G_{tonic} troughs, which were in turn preceded by maximal firing synchronicity (Figures S7A and S7B), in line with the experimental data. Thus, increased interneuronal activity initially boosts network inhibition, after which disinhibition (probably shunting[12,15]) prevails, at which point synchronized network discharges are likely to occur, as generally predicted by our theoretical model. Throughout these recordings, we did not detect depolarization transients that might arise from significant $[K^+]_{out}$ elevations due to ChR2

(C) Traces as in (A) expanded: raw data (black), and its 2 Hz low-pass (G_{tonic}; magenta) and high-pass (blue) filtered components.
(D) Example, average spectrogram plots (mean ± 95 CI; high and low frequency bands, as indicated); data over multiple IIEs in one slice.
(E) Summary of the analysis shown in (D), for n = 7 slices: bar graphs, relative spectral power (bars, mean ± SEM; dots, individual experiments), before and after the G_{tonic} peak, as indicated. *p < 0.05, one-sample (left) and paired-sample (middle) Student's t test.
(F) Illustration of dual cell-attached recordings of FS PV+ interneurons (green, 50 μM Alexa Fluor 488; magenta, tdTomato) and voltage-clamp recording of a CA1 pyramidal cell (50 μM AF488). Scale bars, 50 μm (left) and 20 μm (right).
(G) Example time course of spiking activity of two simultaneously recorded FS PV+ cells (top and middle traces), and IPSCs in a pyramidal neuron (bottom, V_{hold} = +10 mV) in high-K^+ solution (10 mM) and 0 Mg^{2+}. Rhythmic network waves are present.
(H) Experiment as in (G) but with no detectable rhythmic waves; notations as in (G).
(I) Summary. Regression slope (mean ± SEM) between PV+ spiking intensity and synchronization parameter in dual-patch recordings. Samples with rhythmic waves (n = 6 cells in 3 slices) and without (n = 12 cells in 6 slices) are shown; *p ~ 0.038, Kruskal-Wallis non-parametric ANOVA (Z = 2.065; dependent datasets within 3 and 6 slices).

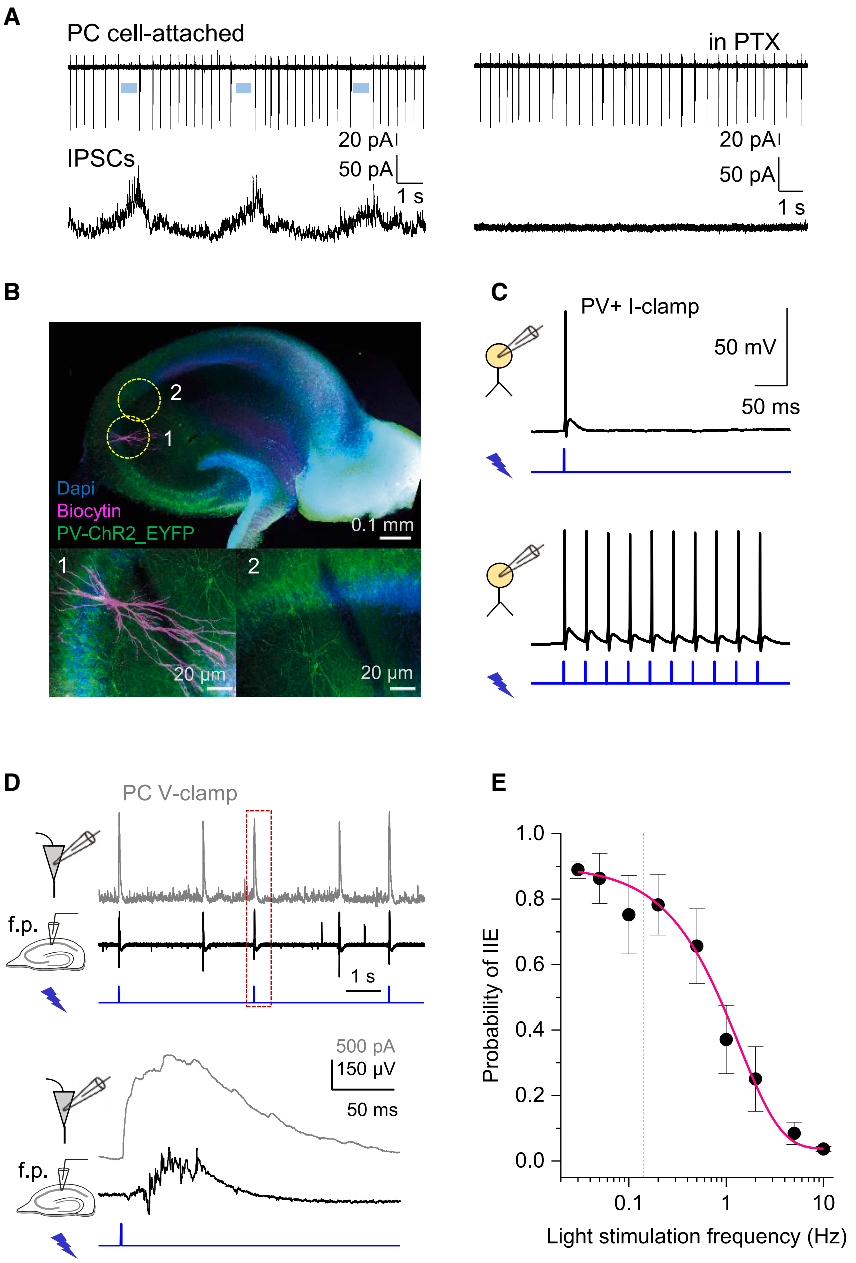

Figure 5. Short photoactivation of FS PV+ interneurons evokes epileptiform burst discharges

(A) Simultaneous cell-attached and whole-cell recordings of action potentials (top trace) and $GABA_AR$-mediated IPSCs (bottom) during $[GABA]_e$ waves (left) and after application of picrotoxin (PTX). Blue segments indicate pauses in PC spiking before the peaks of IPSCs. See also Figure S5A.

(B) Transverse hippocampal slice of PV::*Cre* × Ai32 mouse (top): green fluorescence shows ChR2 expression in PV+ cells against DAPI (blue) nuclear counterstain (STAR Methods). Images 1–2 (areas indicated by dotted circles above): post hoc identification of a CA3 pyramidal neuron (biocytin-filled, magenta; 1) after a whole-cell recording shown below in (D).

(C) Current-clamp recordings from a FS PV+ interneuron activated by a single (left) or repetitive (right) blue light pulses (1 ms, 470 nm).

(D) Example of pyramidal neuron IPSCs (gray trace, top; V_{hold} = +10 mV) and field potentials (black trace, middle): photo-stimulation of FS PV+ cells (blue trace, bottom; 1-ms pulse) triggers large $GABA_AR$ currents and epileptiform bursts similar to those occurring spontaneously (e.g., Figure 1). 5 mM K^+, 0 Mg^{2+} solution. See Figures S5B–S5D for further detail.

(E) Statistical summary: probability of evoking an interictal event (IIE, mean ± SEM, n = 3–7 slices for individual values) as a function of light stimulation frequency. The occurrence of failures increases with stimulation frequency. The average frequency of spontaneous IIE (0.14 ± 0.03 Hz, n = 7 slices, dotted line) falls within the range of optimal frequencies to entrain the network with light stimuli. Above 0.2 Hz stimulation, the network enters into a refractory state. See Figure S6A for an example.

activation,[59] probably because the conditions of high potassium and brief optogenetic stimuli in our case were outside the range for such effects.

GABA uptake controls the frequency of periodic epileptiform events

The rate of GABA uptake depends on the numbers of available GABA transporters, dictating how rapidly $[GABA]_e$ returns to its equilibrium level. The latter level, however, depends on the transporter kinetics, not on their number (unless this number is negligible), according to the first principles of reaction-kinetic theory and assuming no consistent change in extracellular GABA supply. This was consistent with our iGABASnFR2 imaging data, indicating no changes in $[GABA]_e$ under partial blockade of GAT-1 (Figure 2F). Similarly, changes in the uptake rate should have little effect on the rapid activation of intra-synaptic receptors that are exposed to thousands of molecules released at once.[60] Thus, reducing the number of GABA transporters should decelerate the $[GABA]_e$ wave dynamics without changing synaptic GABAergic transmission. To test these principles theoretically, we simulated a 1,500-strong cell network (as in Figure S4) and found that increasing the GABA uptake rate up to a certain level (~0.016 ms^{-1}) could drastically reduce the $[GABA]_e$ dynamic range, thus effectively suppressing activity rhythms (Figure 7A, top). Conversely, slowing down GABA uptake allowed $[GABA]_e$ to fluctuate rhythmically, with lower decay rates producing slower interneuronal network activities (Figure 7A).

To test whether this causality is indeed present in real neuronal networks, we monitored IIEs in slices, while adding a progressively increasing concentration of NO-711 (as in Figure 2F) up to 10 µM, at which point its effect should saturate.[61] Reducing the fraction of available GAT-1 should thus slow down $[GABA]_e$ reequilibration without affecting phasic GABAergic transmission or the basal $[GABA]_e$ level. Strikingly, partial GAT-1 blockade

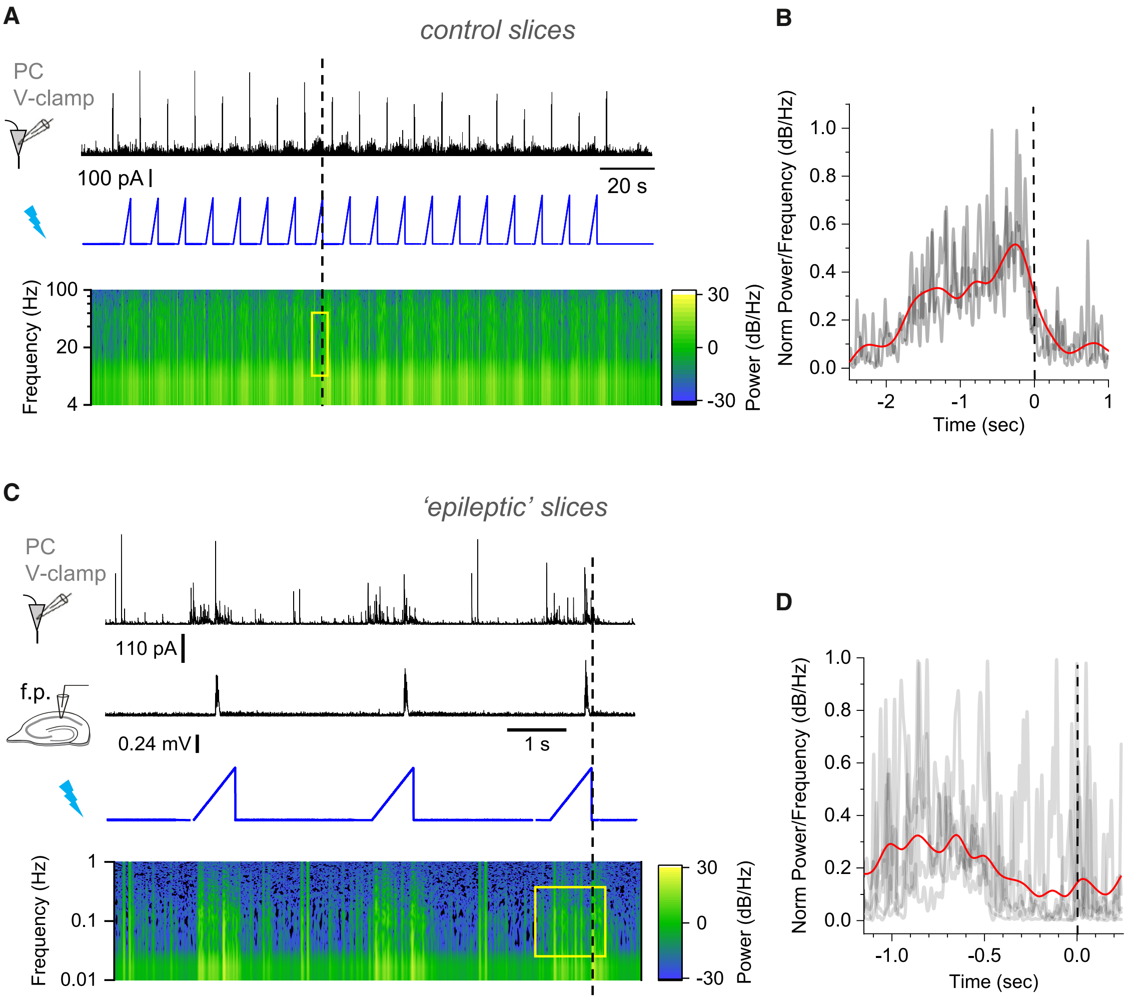

Figure 6. Optogenetically evoked GABA waves entrain epileptiform burst discharges

(A) $GABA_AR$ current in pyramidal neurons (V_{hold} = +10 mV, normal aCSF solution; black trace, top) in response to progressive opto-activation of FS PV+ interneurons (blue, middle), with the IPSC power spectrum density (bottom). Dotted line, end of individual light ramps.

(B) Average spectral power density (5–50 Hz interval, n = 18 ramp stimulations). Time window as exemplified by yellow rectangle in (A) near light intensity peaks (dotted line).

(C) Tests in epileptogenic tissue (0 Mg^{2+} and 5 mM K^+ aCSF): $GABA_AR$ current in pyramidal neurons (black trace, top) and local field potentials (middle) during opto-activation (blue trace), with power spectrum density (bottom). Note that interictal bursts occur toward the end of the stimulation ramp (vertical dotted line). See Figure S6B for further recording detail and Figures S7A and S7B for enforced rhythm entraining reproduced by the neural network model.

(D) Average of spectral power density for time window exemplified by yellow rectangle in (C); notations as in (B).

indeed reduced the frequency of IIEs, in exact correspondence with the modeling predictions (Figures 7B and 7C). In some experiments, we also monitored local field potential (LFP) and pyramidal neuron activity simultaneously, which again revealed transients of tonic GABA current prior to field spikes (Figure S7C). These results provide compelling evidence that $[GABA]_e$ waves play a key role in the pacing of rhythmic activity in interneuronal networks.

DISCUSSION

Interneuronal networks, in particular FS PV+ cells, can exhibit rhythmic synchronization at IIE frequencies, with or without fast glutamatergic signaling. Among interneurons, excitation can arise from depolarizing currents mediated by $GABA_ARs$,[1] depending on receptor reversal potential and the activity-driven dynamics of intracellular chloride and $[K^+]_{out}$. The underlying machinery involves the key chloride-potassium extruder, KCC2, and possibly other transporter mechanisms,[62,63] but their exact nature is outside the scope of the present study.

Here, we sought to understand how fluctuations in G_{tonic}, which are driven mainly by the activity-dependent dynamics of $[GABA]_e$, can entrain slow periodic network activity such as IIEs. The waves of $[GABA]_e$ provide a volume-transmitted feedback signal that modulates neuronal firing. Reducing G_{tonic} from its steady-state level boosts CA1 pyramidal cell firing, while having little effect on interneuronal activity,[64] pointing to the tonic disinhibition of PCs. However, increasing G_{tonic} above a certain

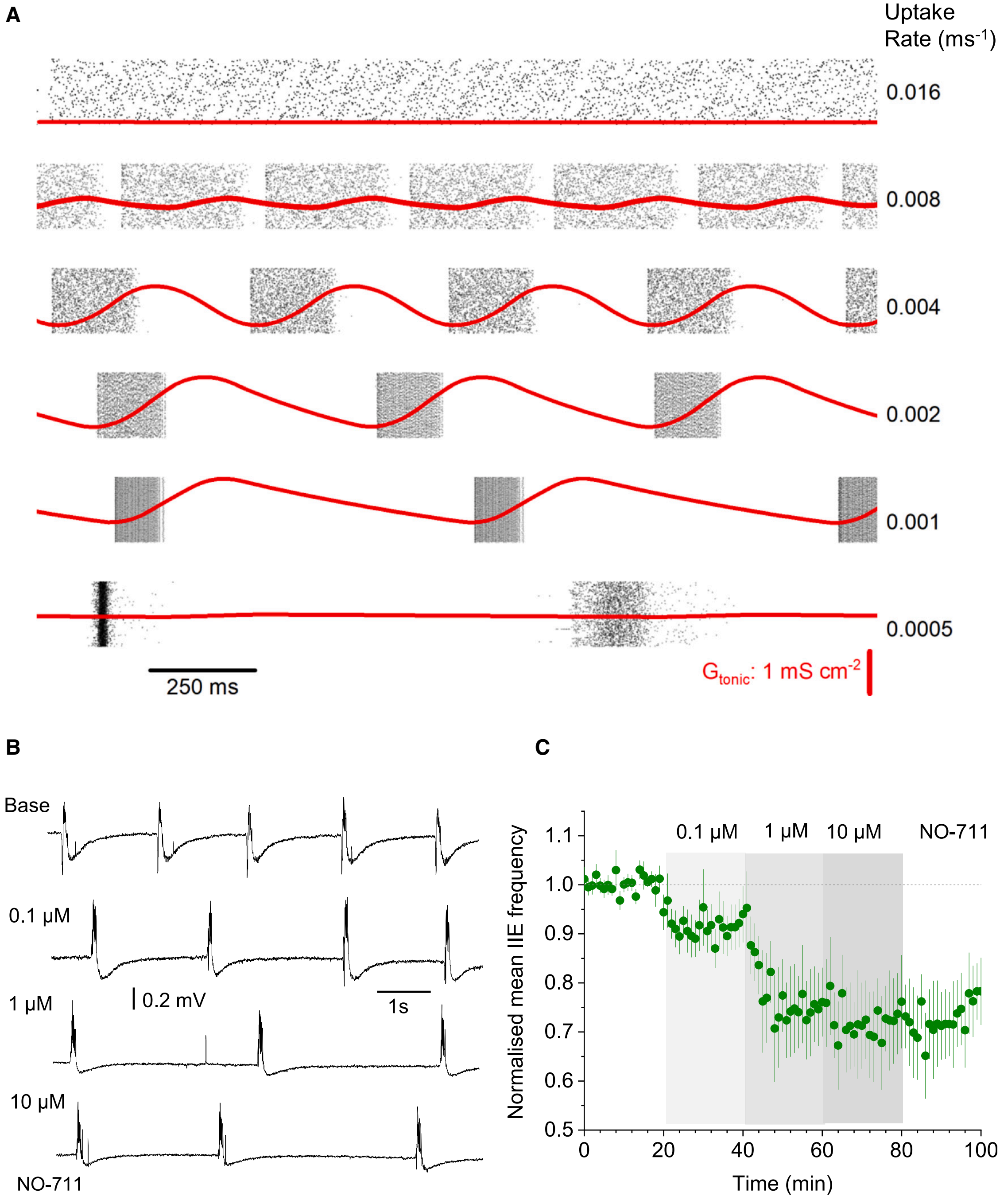

Figure 7. GABA uptake rate controls rhythmic activity of interneuronal networks

(A) Raster plots of interneuron spiking (dots; for presentation clarity, 6,000 randomly selected spike events shown only) and time course of G_{tonic} at different GABA uptake rates (red lines), as indicated. Key model parameters: cell number N = 1,500, intra-network peak synaptic conductance G_{ii} = 0.096 mS cm^{-2}; *E*-currents (Poisson series) with average synaptic conductance g_s = 0.05 nS, decay constant tau = 3 ms, and frequency f_s = 20 Hz; GABA$_A$R reversal potential V_{GABA} = −53 mV, GABA release factor $Af = 10^{-8}$ nS cm^{-2} ms^{-1}, G_{pump} = 0.004 ms^{-1} (see STAR Methods for further detail). G_{tonic} scale (bottom panel) applies throughout.

(B) Examples of interictal spiking recorded in slice, under basal conditions (0 Mg^{2+} and 5 mM [K^+], base), and during subsequent bath applications of the GABA uptake blocker NO-711 at increasing concentrations, as indicated. See Figure S7C for an example containing CA1 pyramidal cell IPSC recording.

(C) Average frequency of interictal events (mean ± SEM, n = 6 slices), normalized to the baseline value, in baseline conditions and during NO-711 application, as indicated.

level could have a similar effect on PCs as it shunts interneuronal activity, as shown earlier.[12,15] At that point, disinhibition (network rebound excitation) of PCs prevails, leading to synchronized network discharges. However, that earlier work[15] explored a steady-state case for [GABA]$_e$—and, therefore, G_{tonic}—with no volume-transmitted GABA signals. In that case, (1) low G_{tonic}

had little effect on synchronization, (2) its initial increase boosted excitation and synchronization, and (3) increasing G_{tonic} further reduced excitability due to shunting, thus reducing neuronal synchronization. In contrast, the present study deals with the nonstationary network dynamics engaging $[GABA]_e$-dependent feedback, leading to a different parameter space for the firing synchronization phenomena. We find that the pattern of network activity and its predisposition to oscillations must depend on the G_{tonic} dynamics driven by GABA release, diffusion, and uptake. However, this does not imply that the GABA wave mechanism described here is the only factor entraining network rhythms. After all, the primary source of GABA waves is synaptic activity of GABAergic interneurons, whereas the target of the waves must involve both synaptic and extrasynaptic $GABA_A$Rs.

Potential limitations of the high potassium model

In the high-K^+ experimental models, depolarization due to elevated $[K^+]_{out}$ varies among neurons and their dendrites and thus provides a stochastic excitatory drive to interneurons when excitatory transmission is blocked. By design, therefore, such interneuron-only models still involve some excitatory drive in triggering interictal activity. In addition, increasing $[K^+]_{out}$ up to 10 mM is likely to elevate internal $[Cl^-]$ up to 6 mM,[65] which helps to set $GABA_A$R reversal potential within the interneuron shunting range and thus prompt epileptiform activity under intact glutamatergic transmission. Whether similar conditions can occur *in vivo* remains to be ascertained.

While increasing $[K^+]_{out}$ by adding KCl has been a common practice to induce epileptiform activity, it may raise some basic questions regarding GABA homeostasis. However, the kinetics of GABA transporters per se is independent of $[K^+]_{out}$ and depends almost linearly on membrane potential.[54] In addition, increasing $[Cl^-]_{out}$ by 5–10 mM from the baseline (normally 110–120 mM) only changes the GABA transport reversal potential V_{GAT} by ~1%–2%, according to the basic expression for V_{GAT} (STAR Methods). Finally, evidence from our imaging experiments with iGABASnFR2 indicates that a 10-mM increase in external [KCl] elevates resting extracellular [GABA] up to 0.6–0.7 μM, which remains virtually unchanged under partial blockade of GAT-1 (Figure 2F). Altogether, these observations suggest that using high potassium is unlikely to engage some poorly controlled concomitant mechanisms of GABA transport that would affect mechanistic interpretations of our data.

$[GABA]_e$ dynamic range and activation of extrasynaptic receptors

We find that during IIEs in hippocampal slices $[GABA]_e$ peaks at about 1.5–2 μM (Figures 1E and 2E). Calibration of the sniffer-patch sensor in acute slices indicates that $[GABA]_e$ is maintained at 0.25–0.5 μM in nominally Mg^{2+}-free aCSF (Figure 1E). This $[GABA]_e$ range is 2–5 times greater than that in quiescent slices at near-physiological $[Mg^{2+}]$ (estimated previously using the same technique[38]), likely because of a higher spontaneous interneuron firing rate. iGABASnFR2 imaging indicates that applying 10 mM $[K^+]_{out}$/0 $[Mg^{2+}]$ elevates $[GABA]_e$ from its basal level by 0.7–0.8 μM (Figure 1F). These estimates are generally in line with the $[GABA]_e$ range reported earlier in hippocampal area CA1.[66] Are these levels of $[GABA]_e$ compatible with the peak values of G_{tonic} documented here using whole-cell recordings?

One factor associated with having significant G_{tonic} is relatively low-affinity, slowly desensitized extrasynaptic $GABA_A$Rs. The broadly expressed high-affinity δ-subunit-containing $GABA_A$Rs were found to become desensitized and almost irresponsive to GABA when $[GABA]_e$ reached ~0.25 μM.[67] Other receptor sub-types with EC_{50} in the range of 0.5–2 μM include native metabotropic $GABA_B$ receptors in L2/3 pyramidal neurons[68] and receptors with subunits α5β3γ2L (0.5–7 μM) and α1β2γ2L (0.6–9 μM) that can generate non-synaptic GABA currents in the hippocampus.[69] Overall, it appears plausible that the $[GABA]_e$ waves reported here find enough receptor targets to provide a significant dynamic range of G_{tonic}.[13] Indeed, we did not observe a consistent or progressive (post-pulse) reduction in IIE-synchronized GABAergic currents, for typical inter-spike intervals, throughout our electrophysiological recordings. This suggested no persistent $GABA_A$R desensitization, which is also consistent with the desensitization kinetics of hippocampal $GABA_A$R shown earlier,[70] given the inter-IIE intervals and the $[GABA]_e$ range observed here.

Mechanisms controlling $[GABA]_e$

GABA that escapes from synaptic clefts during interneuronal discharges is the most likely source of $[GABA]_e$, although there have also been studies reporting GABA release from astroglia.[71–73] One theoretically plausible mechanism that might regulate $[GABA]_e$ is the reversal mode of GABA transporters.[74] Four GABA transporter types (GAT1-3 and BGT1), all showing potassium- and voltage-dependent kinetics, are expressed in both neurons and glial cells.[75–77] Intense excitatory activity—for instance, during epileptiform bursts—can increase extracellular $[K^+]$ to 12–15 mM[78,79] and could, in theory, reverse GABA transport, thus generating GABA efflux.[80] Intriguingly, while cultured neurons show depolarization-dependent GABA efflux via GAT-1 under negligibly low $[GABA]_e$,[74] any significant level of $[GABA]_e$ should prevent the reverse GAT-1 mode.[54] Because resting $[GABA]_e$, in our experiments, is in the range of 0.1–0.3 μM (see above), any significant transporter-mediated GABA release is unlikely. Finally, regulation of $[GABA]_e$ could arise from the Bestrophin 1 Ca^{2+}-dependent Cl^- channel[81] found predominantly in glial cells.[82] However, this mechanism is unlikely to play a role here, as the operation of this channel does not appear correlated with neuronal activity.[81]

Constraining parameters of simulated neural networks

Our study explores a well-established FS interneuronal network model[83] that incorporates G_{tonic} driven by activity-dependent $[GABA]_e$.[15,40] While replicating the key aspects of interictal hippocampal activity, this model has certain limitations. First, we used a near-linear relationship between $[GABA]_e$ and G_{tonic}, whereas in reality it incorporates a more complex dependence.[12,15] This might explain why the experimental time courses of $[GABA]_e$ and G_{tonic} do not necessarily match. Second, to isolate the role of FS interneuronal networks in pacing hippocampal oscillations, the majority of our models mimicked PC activity by an artificial, interneuron-independent stochastic excitatory input. However, introducing a network that contained both interneurons and PCs (Figure 3) revealed spiking behaviors that were fully consistent with the key features of interneuron-only networks, as well as with experimental observations. Finally,

CellPress
OPEN ACCESS

astroglial GABA uptake must play an important role in pacing network rhythms as it shapes the $[GABA]_e$ waveform. It was a revelation that our model could faithfully reproduce the relationship between the GABA uptake rate and the frequency of epileptiform events documented experimentally (Figure 7). This observation also implicated GAT-1, probably both astroglial and neuronal, as a major player in pacing rhythmic activities of interneuronal ensembles.

Interneurons and excitatory action in rhythm initiation

In high-K^+ experimental models, fluctuating cell depolarization provides a stochastic excitatory drive for interneurons. In the intact brain, however, this role belongs with PC firing, which must be an important contributor to epileptiform activity. Our experiments consistently point to an increase in tonic inhibition prior to an IIE onset. This invokes two possibilities: either interictal activity is due to the synchronized disinhibition of PCs or interneuron firing itself drives IIEs. We find that synchronized interneuronal network activity can determine the timing of coordinated pyramidal cell bursts, confirming the former suggestion.[84,85] At the same time, interneuronal activity per se can make an important contribution to epileptiform discharges. Indeed, another potentially important mechanism regulating GABAergic excitation is $HCO3^-$ transport[86,87]: its potential involvement in modulating IIE rhythms remains to be established.

It is important to highlight some well-documented mechanisms that drive interneuronal network oscillations and may not depend on $[GABA]_e$ fluctuations. First, the so-called giant depolarizing potentials, the wave-like, second-long events occurring at 0.1–0.2 Hz during development.[88–92] GDPs are thought to involve the synergistic activity of pyramidal cells and interneurons.[1] The other mechanism involves high-frequency hippocampal sharp-wave ripples,[93–95] representing highly synchronous activity bursts. In the ventral hippocampus, such ripples can occur in a periodic pattern, with a frequency of 7–14 Hz,[96] similar to the theta rhythm. These oscillatory patterns could arise purely from the interaction of excitatory and GABAergic neurons, a mechanism qualitatively different from interictal activity resulting from periodic $[GABA]_e$ waves. Inevitably, although FS PV+ interneurons have been considered major players in epileptiform activity, other interneurons, PCs, and non-neuronal cell types play their own important parts that could differ among different brain regions.

STAR★METHODS

Detailed methods are provided in the online version of this paper and include the following:

- KEY RESOURCES TABLE
- RESOURCE AVAILABILITY
  - Lead contact
  - Materials availability
  - Data and code availability
- EXPERIMENTAL MODEL AND SUBJECT DETAILS
  - Animal experimentation
  - Experimental model designs across preparations
  - Acute slice preparation
  - Organotypic slice culture preparation and biolistic transfection of iGABASnFR2
- METHOD DETAILS
  - Viral transduction of iGABASnFR2 for acute slice experiments
  - Outside-out patch recordings
  - Analysis of single-channel recordings and iGABASnFR2 titration
  - Two-photon excitation imaging
  - Targeted cell-attached recordings and optogenetic experiments
  - Interictal discharge analysis
  - Modeling: Networks of interneurons and pyramidal neurons
  - Modeling: Network synchronization
  - Modeling: Tonic GABA conductance
  - Modeling: $[GABA]_e$ dynamics
  - Modeling: Code detail
- QUANTIFICATION AND STATISTICAL ANALYSIS

SUPPLEMENTAL INFORMATION

Supplemental information can be found online at https://doi.org/10.1016/j.cub.2023.02.051.

ACKNOWLEDGMENTS

This work was supported by Wellcome Principal Fellowship (212251/Z/18/Z and 212285/Z/18/Z), Wellcome Collaborative Award (UNS120639 - 223131/Z/21/Z), Epilepsy Research UK (F1901), MRC (MR/W019752/1, MR/V013556/1, and MR/V034758/1), NC3Rs (NC/X001067/1), and ERC Advanced Grant (323113). Optimization and parallelization of ARACHNE algorithms for extended neural network simulations was provided by AMC Bridge (Waltham, MA) and web security by Cyber Curio (Berkhamsted, UK).

AUTHOR CONTRIBUTIONS

L.P.S. and D.A.R. narrated the study. I.P. and V.M. designed and carried out electrophysiological and optogenetic studies. N.C., O.K., and T.P.J. designed, carried out, and analyzed iGABASnFR2 imaging experiments. S.S. carried out sniffer-patch experiments. O.T. carried out control-probing K^+ imaging tests. L.P.S. designed and carried out network modeling studies and data analyses. J.S.M., L.L.L., J.P.H., and I.K. supplied optical GABA sensors and related protocols. The development of iGABASnFR2 was conducted under the aegis of the HHMI Janelia GENIE Project at Janelia Research Campus. D.M.K. and M.C.W. designed optogenetic experiments. D.A.R. designed selected experiments and simulations, carried out selected analyses, and wrote the manuscript draft, with contributions from V.M., L.P.S., and all other authors.

DECLARATION OF INTERESTS

The authors declare no competing interests.

INCLUSION AND DIVERSITY

We support inclusive, diverse, and equitable conduct of research.

Received: May 16, 2022
Revised: January 5, 2023
Accepted: February 15, 2023
Published: March 14, 2023

REFERENCES

1. Ben-Ari, Y., Gaiarsa, J.L., Tyzio, R., and Khazipov, R. (2007). GABA: a pioneer transmitter that excites immature neurons and generates primitive oscillations. Physiol. Rev. *87*, 1215–1284.

2. van der Zeyden, M., Oldenziel, W.H., Rea, K., Cremers, T.I., and Westerink, B.H. (2008). Microdialysis of GABA and glutamate: analysis, interpretation and comparison with microsensors. Pharmacol. Biochem. Behav. *90*, 135–147.
3. Overstreet, L.S., and Westbrook, G.L. (2003). Synapse density regulates independence at unitary inhibitory synapses. J. Neurosci. *23*, 2618–2626.
4. Scanziani, M. (2000). GABA spillover activates postsynaptic GABA(B) receptors to control rhythmic hippocampal activity. Neuron *25*, 673–681.
5. Loo, D.D.F., Eskandari, S., Boorer, K.J., Sarkar, H.K., and Wright, E.M. (2000). Role of Cl- in electrogenic Na+-coupled cotransporters GAT1 and SGLT1. J. Biol. Chem. *275*, 37414–37422.
6. Bicho, A., and Grewer, C. (2005). Rapid substrate-induced charge movements of the GABA transporter GAT1. Biophys. J. *89*, 211–231.
7. MacAulay, N., Meinild, A.K., Zeuthen, T., and Gether, U. (2003). Residues in the extracellular loop 4 are critical for maintaining the conformational equilibrium of the gamma-aminobutyric acid transporter-1. J. Biol. Chem. *278*, 28771–28777.
8. Glykys, J., and Mody, I. (2007). The main source of ambient GABA responsible for tonic inhibition in the mouse hippocampus. J. Physiol. *582*, 1163–1178.
9. Marchionni, I., and Maccaferri, G. (2009). Quantitative dynamics and spatial profile of perisomatic GABAergic input during epileptiform synchronization in the CA1 hippocampus. J. Physiol. *587*, 5691–5708.
10. Brickley, S.G., Revilla, V., Cull-Candy, S.G., Wisden, W., and Farrant, M. (2001). Adaptive regulation of neuronal excitability by a voltage-independent potassium conductance. Nature *409*, 88–92.
11. Pavlov, I., Savtchenko, L.P., Kullmann, D.M., Semyanov, A., and Walker, M.C. (2009). Outwardly rectifying tonically active GABAA receptors in pyramidal cells modulate neuronal offset, not gain. J. Neurosci. *29*, 15341–15350.
12. Song, I., Savtchenko, L., and Semyanov, A. (2011). Tonic excitation or inhibition is set by GABA(A) conductance in hippocampal interneurons. Nat. Commun. *2*, 376.
13. Scimemi, A., Semyanov, A., Sperk, G., Kullmann, D.M., and Walker, M.C. (2005). Multiple and plastic receptors mediate tonic $GABA_A$ receptor currents in the hippocampus. J. Neurosci. *25*, 10016–10024.
14. Mann, E.O., and Mody, I. (2010). Control of hippocampal gamma oscillation frequency by tonic inhibition and excitation of interneurons. Nat. Neurosci. *13*, 205–212.
15. Pavlov, I., Savtchenko, L.P., Song, I., Koo, J., Pimashkin, A., Rusakov, D.A., and Semyanov, A. (2014). Tonic GABAA conductance bidirectionally controls interneuron firing pattern and synchronization in the CA3 hippocampal network. Proc. Natl. Acad. Sci. USA *111*, 504–509.
16. Pouille, F., and Scanziani, M. (2001). Enforcement of temporal fidelity in pyramidal cells by somatic feed-forward inhibition. Science *293*, 1159–1163.
17. Pavlov, I., Scimemi, A., Savtchenko, L., Kullmann, D.M., and Walker, M.C. (2011). I(h)-mediated depolarization enhances the temporal precision of neuronal integration. Nat. Commun. *2*, 199.
18. Sylantyev, S., Savtchenko, L.P., O'Neill, N., and Rusakov, D.A. (2020). Extracellular GABA waves regulate coincidence detection in excitatory circuits. J. Physiol. *598*, 4047–4062.
19. Karlócai, M.R., Kohus, Z., Káli, S., Ulbert, I., Szabó, G., Máté, Z., Freund, T.F., and Gulyás, A.I. (2014). Physiological sharp wave-ripples and interictal events in vitro: what's the difference? Brain *137*, 463–485.
20. Miri, M.L., Vinck, M., Pant, R., and Cardin, J.A. (2018). Altered hippocampal interneuron activity precedes ictal onset. eLife *7*, e40750.
21. Gnatkovsky, V., Librizzi, L., Trombin, F., and de Curtis, M. (2008). Fast activity at seizure onset is mediated by inhibitory circuits in the entorhinal cortex in vitro. Ann. Neurol. *64*, 674–686.
22. Neumann, A.R., Raedt, R., Steenland, H.W., Sprengers, M., Bzymek, K., Navratilova, Z., Mesina, L., Xie, J., Lapointe, V., Kloosterman, F., et al. (2017). Involvement of fast-spiking cells in ictal sequences during spontaneous seizures in rats with chronic temporal lobe epilepsy. Brain *140*, 2355–2369.
23. Librizzi, L., Losi, G., Marcon, I., Sessolo, M., Scalmani, P., Carmignoto, G., and de Curtis, M. (2017). Interneuronal network activity at the onset of seizure-like events in entorhinal cortex slices. J. Neurosci. *37*, 10398–10407.
24. Timofeev, I., Grenier, F., and Steriade, M. (2002). The role of chloride-dependent inhibition and the activity of fast-spiking neurons during cortical spike-wave electrographic seizures. Neuroscience *114*, 1115–1132.
25. Ledri, M., Madsen, M.G., Nikitidou, L., Kirik, D., and Kokaia, M. (2014). Global optogenetic activation of inhibitory interneurons during epileptiform activity. J. Neurosci. *34*, 3364–3377.
26. Sessolo, M., Marcon, I., Bovetti, S., Losi, G., Cammarota, M., Ratto, G.M., Fellin, T., and Carmignoto, G. (2015). Parvalbumin-positive inhibitory interneurons oppose propagation but favor generation of focal epileptiform activity. J. Neurosci. *35*, 9544–9557.
27. Yekhlef, L., Breschi, G.L., Lagostena, L., Russo, G., and Taverna, S. (2015). Selective activation of parvalbumin- or somatostatin-expressing interneurons triggers epileptic seizurelike activity in mouse medial entorhinal cortex. J. Neurophysiol. *113*, 1616–1630.
28. Assaf, F., and Schiller, Y. (2016). The antiepileptic and ictogenic effects of optogenetic neurostimulation of PV-expressing interneurons. J. Neurophysiol. *116*, 1694–1704.
29. Chang, M., Dian, J.A., Dufour, S., Wang, L., Moradi Chameh, H., Ramani, M., Zhang, L., Carlen, P.L., Womelsdorf, T., and Valiante, T.A. (2018). Brief activation of GABAergic interneurons initiates the transition to ictal events through post-inhibitory rebound excitation. Neurobiol. Dis. *109*, 102–116.
30. Magloire, V., Mercier, M.S., Kullmann, D.M., and Pavlov, I. (2019). GABAergic interneurons in seizures: investigating causality with optogenetics. Neuroscientist *25*, 344–358.
31. Toyoda, I., Fujita, S., Thamattoor, A.K., and Buckmaster, P.S. (2015). Unit activity of hippocampal interneurons before spontaneous seizures in an animal model of temporal lobe epilepsy. J. Neurosci. *35*, 6600–6618.
32. Truccolo, W., Donoghue, J.A., Hochberg, L.R., Eskandar, E.N., Madsen, J.R., Anderson, W.S., Brown, E.N., Halgren, E., and Cash, S.S. (2011). Single-neuron dynamics in human focal epilepsy. Nat. Neurosci. *14*, 635–641.
33. Parrish, R.R., Codadu, N.K., Mackenzie-Gray Scott, C., and Trevelyan, A.J. (2019). Feedforward inhibition ahead of ictal wavefronts is provided by both parvalbumin- and somatostatin-expressing interneurons. J. Physiol. *597*, 2297–2314.
34. Grasse, D.W., Karunakaran, S., and Moxon, K.A. (2013). Neuronal synchrony and the transition to spontaneous seizures. Exp. Neurol. *248*, 72–84.
35. Huguenard, J.R., and McCormick, D.A. (1992). Simulation of the currents involved in rhythmic oscillations in thalamic relay neurons. J. Neurophysiol. *68*, 1373–1383.
36. Marvin, J.S., Shimoda, Y., Magloire, V., Leite, M., Kawashima, T., Jensen, T.P., Kolb, I., Knott, E.L., Novak, O., Podgorski, K., et al. (2019). A genetically encoded fluorescent sensor for in vivo imaging of GABA. Nat. Methods *16*, 763–770.
37. Isaacson, J.S., Solís, J.M., and Nicoll, R.A. (1993). Local and diffuse synaptic actions of Gaba in the hippocampus. Neuron *10*, 165–175.
38. Wlodarczyk, A.I., Sylantyev, S., Herd, M.B., Kersanté, F., Lambert, J.J., Rusakov, D.A., Linthorst, A.C., Semyanov, A., Belelli, D., Pavlov, I., and Walker, M.C. (2013). GABA-independent GABAA receptor openings maintain tonic currents. J. Neurosci. *33*, 3905–3914.
39. Kolb, I., Hasseman, J., Tsegaye, G., Tsang, A., Reep, D., Zheng, J., Arthur, B., Marvin, J.S., Svoboda, K., Looger, L.L., and Korff, W. (2022). Optimization of Genetically Encoded GABA Indicator. Janelia Research Campus. Dataset. https://doi.org/10.25378/janelia.19709311.v3.

CellPress
OPEN ACCESS

40. Savtchenko, L.P., and Rusakov, D.A. (2014). Regulation of rhythm genesis by volume-limited, astroglia-like signals in neural networks. Philos. Trans. R. Soc. Lond. B Biol. Sci. *369*, 20130614.
41. Aleksin, S.G., Zheng, K., Rusakov, D.A., and Savtchenko, L.P. (2017). ARACHNE: a neural-neuroglial network builder with remotely controlled parallel computing. PLoS Comput. Biol. *13*, e1005467.
42. Mody, I., Lambert, J.D.C., and Heinemann, U. (1987). Low extracellular magnesium induces epileptiform activity and spreading depression in rat hippocampal slices. J. Neurophysiol. *57*, 869–888.
43. Yaari, Y., Konnerth, A., and Heinemann, U. (1986). Nonsynaptic epileptogenesis in the mammalian hippocampus in vitro. II. Role of extracellular potassium. J. Neurophysiol. *56*, 424–438.
44. Sylantyev, S., and Rusakov, D.A. (2013). Sub-millisecond ligand probing of cell receptors with multiple solution exchange. Nat. Protoc. *8*, 1299–1306.
45. de Curtis, M., and Avoli, M. (2016). GABAergic networks jump-start focal seizures. Epilepsia *57*, 679–687.
46. Armbruster, M., Dulla, C.G., and Diamond, J.S. (2020). Effects of fluorescent glutamate indicators on neurotransmitter diffusion and uptake. eLife *9*, e54441.
47. Kopach, O., Zheng, K.Y., and Rusakov, D.A. (2020). Optical monitoring of glutamate release at multiple synapses in situ detects changes following LTP induction. Mol. Brain *13*, 39.
48. Jensen, T.P., Zheng, K.Y., Cole, N., Marvin, J.S., Looger, L.L., and Rusakov, D.A. (2019). Multiplex imaging relates quantal glutamate release to presynaptic Ca2+ homeostasis at multiple synapses in situ. Nat. Commun. *10*, 1414.
49. Henneberger, C., Bard, L., Panatier, A., Reynolds, J.P., Kopach, O., Medvedev, N.I., Minge, D., Herde, M.K., Anders, S., Kraev, I., et al. (2020). LTP induction boosts glutamate spillover by driving withdrawal of perisynaptic astroglia. Neuron *108*, 919–936.e11.
50. Schlingloff, D., Káli, S., Freund, T.F., Hájos, N., and Gulyás, A.I. (2014). Mechanisms of sharp wave initiation and ripple generation. J. Neurosci. *34*, 11385–11398.
51. Ferguson, K.A., Huh, C.Y., Amilhon, B., Williams, S., and Skinner, F.K. (2013). Experimentally constrained CA1 fast-firing parvalbumin-positive interneuron network models exhibit sharp transitions into coherent high frequency rhythms. Front. Comput. Neurosci. *7*, 144.
52. Rich, S., Chameh, H.M., Rafiee, M., Ferguson, K., Skinner, F.K., and Valiante, T.A. (2019). Inhibitory network bistability explains increased interneuronal activity prior to seizure onset. Front. Neural Circuits *13*, 81.
53. Y Ho, E.C.Y., and Truccolo, W. (2016). Interaction between synaptic inhibition and glial-potassium dynamics leads to diverse seizure transition modes in biophysical models of human focal seizures. J. Comput. Neurosci. *41*, 225–244.
54. Savtchenko, L., Megalogeni, M., Rusakov, D.A., Walker, M.C., and Pavlov, I. (2015). Synaptic GABA release prevents GABA transporter type-1 reversal during excessive network activity. Nat. Commun. *6*, 6597.
55. Fasoli, D., Cattani, A., and Panzeri, S. (2018). Transitions between asynchronous and synchronous states: a theory of correlations in small neural circuits. J. Comput. Neurosci. *44*, 25–43.
56. Sanzeni, A., Akitake, B., Goldbach, H.C., Leedy, C.E., Brunel, N., and Histed, M.H. (2020). Inhibition stabilization is a widespread property of cortical networks. eLife *9*, e54875.
57. Avoli, M., and de Curtis, M. (2011). GABAergic synchronization in the limbic system and its role in the generation of epileptiform activity. Prog. Neurobiol. *95*, 104–132.
58. Huberfeld, G., Menendez de la Prida, L., Pallud, J., Cohen, I., Le Van Quyen, M., Adam, C., Clemenceau, S., Baulac, M., and Miles, R. (2011). Glutamatergic pre-ictal discharges emerge at the transition to seizure in human epilepsy. Nat. Neurosci. *14*, 627–634.
59. Octeau, J.C., Gangwani, M.R., Allam, S.L., Tran, D., Huang, S., Hoang-Trong, T.M., Golshani, P., Rumbell, T.H., Kozloski, J.R., and Khakh, B.S. (2019). Transient, consequential increases in extracellular potassium ions accompany Channelrhodopsin2 excitation. Cell Rep. *27*, 2249–2261.e7.
60. Zheng, K., Scimemi, A., and Rusakov, D.A. (2008). Receptor actions of synaptically released glutamate: the role of transporters on the scale from nanometers to microns. Biophys. J. *95*, 4584–4596.
61. Kersanté, F., Rowley, S.C.S., Pavlov, I., Gutièrrez-Mecinas, M., Semyanov, A., Reul, J.M.H.M., Walker, M.C., and Linthorst, A.C.E. (2013). A functional role for both gamma-aminobutyric acid (GABA) transporter-1 and GABA transporter-3 in the modulation of extracellular GABA and GABAergic tonic conductances in the rat hippocampus. J. Physiol. *591*, 2429–2441.
62. Rivera, C., Voipio, J., Payne, J.A., Ruusuvuori, E., Lahtinen, H., Lamsa, K., Pirvola, U., Saarma, M., and Kaila, K. (1999). The K+/Cl- co-transporter KCC2 renders GABA hyperpolarizing during neuronal maturation. Nature *397*, 251–255.
63. Virtanen, M.A., Uvarov, P., Mavrovic, M., Poncer, J.C., and Kaila, K. (2021). The multifaceted roles of KCC2 in cortical development. Trends Neurosci. *44*, 378–392.
64. Semyanov, A., Walker, M.C., and Kullmann, D.M. (2003). GABA uptake regulates cortical excitability via cell type-specific tonic inhibition. Nat. Neurosci. *6*, 484–490.
65. DeFazio, R.A., Keros, S., Quick, M.W., and Hablitz, J.J. (2000). Potassium-coupled chloride cotransport controls intracellular chloride in rat neocortical pyramidal neurons. J. Neurosci. *20*, 8069–8076.
66. Farahmandfar, M., Zarrindast, M.R., Kadivar, M., Karimian, S.M., and Naghdi, N. (2011). The effect of morphine sensitization on extracellular concentrations of GABA in dorsal hippocampus of male rats. Eur. J. Pharmacol. *669*, 66–70.
67. Bright, D.P., Renzi, M., Bartram, J., McGee, T.P., MacKenzie, G., Hosie, A.M., Farrant, M., and Brickley, S.G. (2011). Profound desensitization by ambient GABA limits activation of δ-containing $GABA_A$ receptors during spillover. J. Neurosci. *31*, 753–763.
68. Wang, Y., Neubauer, F.B., Lüscher, H.R., and Thurley, K. (2010). GABAB receptor-dependent modulation of network activity in the rat prefrontal cortex in vitro. Eur. J. Neurosci. *31*, 1582–1594.
69. Lagrange, A.H., Hu, N., and Macdonald, R.L. (2018). GABA beyond the synapse: defining the subtype-specific pharmacodynamics of non-synaptic GABAA receptors. J. Physiol. *596*, 4475–4495.
70. Berger, T., Schwarz, C., Kraushaar, U., and Monyer, H. (1998). Dentate gyrus basket cell GABAA receptors are blocked by Zn2+ via changes of their desensitization kinetics: an in situ patch-clamp and single-cell PCR study. J. Neurosci. *18*, 2437–2448.
71. Lee, M., McGeer, E.G., and McGeer, P.L. (2011). Mechanisms of GABA release from human astrocytes. Glia *59*, 1600–1611.
72. Héja, L., Barabás, P., Nyitrai, G., Kékesi, K.A., Lasztóczi, B., Toke, O., Tárkányi, G., Madsen, K., Schousboe, A., Dobolyi, A., et al. (2009). Glutamate uptake triggers transporter-mediated GABA release from astrocytes. PLoS One *4*, e7153.
73. Le Meur, K., Mendizabal-Zubiaga, J., Grandes, P., and Audinat, E. (2012). GABA release by hippocampal astrocytes. Front. Comput. Neurosci. *6*, 59.
74. Wu, Y., Wang, W., Díez-Sampedro, A., and Richerson, G.B. (2007). Nonvesicular inhibitory neurotransmission via reversal of the GABA transporter GAT-1. Neuron *56*, 851–865.
75. Borden, L.A. (1996). GABA transporter heterogeneity: pharmacology and cellular localization. Neurochem. Int. *29*, 335–356.
76. Gadea, A., and López-Colomé, A.M. (2001). Glial transporters for glutamate, glycine, and GABA: II. GABA transporters. J. Neurosci. Res. *63*, 461–468.
77. Conti, F., Melone, M., De Biasi, S., Minelli, A., Brecha, N.C., and Ducati, A. (1998). Neuronal and glial localization of GAT-1, a high-affinity gamma-aminobutyric acid plasma membrane transporter, in human cerebral cortex: with a note on its distribution in monkey cortex. J. Comp. Neurol. *396*, 51–63.

78. Krnjević, K., Morris, M.E., and Reiffenstein, R.J. (1980). Changes in extracellular Ca2+ and K+ activity accompanying hippocampal discharges. Can. J. Physiol. Pharmacol. *58*, 579–582.

79. Somjen, G.G., and Giacchino, J.L. (1985). Potassium and calcium concentrations in interstitial fluid of hippocampal formation during paroxysmal responses. J. Neurophysiol. *53*, 1098–1108.

80. Wu, Y., Wang, W., and Richerson, G.B. (2001). GABA transaminase inhibition induces spontaneous and enhances depolarization-evoked GABA efflux via reversal of the GABA transporter. J. Neurosci. *21*, 2630–2639.

81. Lee, S., Yoon, B.E., Berglund, K., Oh, S.J., Park, H., Shin, H.S., Augustine, G.J., and Lee, C.J. (2010). Channel-mediated tonic GABA release from glia. Science *330*, 790–796.

82. Oh, S.J., and Lee, C.J. (2017). Distribution and function of the Bestrophin-1 (Best1) channel in the brain. Exp. Neurobiol. *26*, 113–121.

83. Wang, X.J., and Buzsáki, G. (1996). Gamma oscillation by synaptic inhibition in a hippocampal interneuronal network model. J. Neurosci. *16*, 6402–6413.

84. Traub, R.D., Jefferys, J.G., and Whittington, M.A. (1999). Functionally relevant and functionally disruptive (epileptic) synchronized oscillations in brain slices. Adv. Neurol. *79*, 709–724.

85. Cobb, S.R., Buhl, E.H., Halasy, K., Paulsen, O., and Somogyi, P. (1995). Synchronization of neuronal activity in hippocampus by individual GABAergic interneurons. Nature *378*, 75–78.

86. Ruusuvuori, E., Li, H., Huttu, K., Palva, J.M., Smirnov, S., Rivera, C., Kaila, K., and Voipio, J. (2004). Carbonic anhydrase isoform VII acts as a molecular switch in the development of synchronous gamma-frequency firing of hippocampal CA1 pyramidal cells. J. Neurosci. *24*, 2699–2707.

87. Kaila, K., Lamsa, K., Smirnov, S., Taira, T., and Voipio, J. (1997). Long-lasting GABA-mediated depolarization evoked by high-frequency stimulation in pyramidal neurons of rat hippocampal slice is attributable to a network-driven, bicarbonate-dependent K+ transient. J. Neurosci. *17*, 7662–7672.

88. Leinekugel, X., Khalilov, I., Ben-Ari, Y., and Khazipov, R. (1998). Giant depolarizing potentials: the septal pole of the hippocampus paces the activity of the developing intact septohippocampal complex in vitro. J. Neurosci. *18*, 6349–6357.

89. Sipilä, S.T., Huttu, K., Soltesz, I., Voipio, J., and Kaila, K. (2005). Depolarizing GABA acts on intrinsically bursting pyramidal neurons to drive giant depolarizing potentials in the immature hippocampus. J. Neurosci. *25*, 5280–5289.

90. Sipilä, S.T., Huttu, K., Voipio, J., and Kaila, K. (2006). Intrinsic bursting of immature CA3 pyramidal neurons and consequent giant depolarizing potentials are driven by a persistent Na+ current and terminated by a slow Ca2+ -activated K+ current. Eur. J. Neurosci. *23*, 2330–2338.

91. Khalilov, I., Minlebaev, M., Mukhtarov, M., and Khazipov, R. (2015). Dynamic changes from depolarizing to hyperpolarizing GABAergic actions during giant depolarizing potentials in the neonatal rat hippocampus. J. Neurosci. *35*, 12635–12642.

92. Kasyanov, A.M., Safiulina, V.F., Voronin, L.L., and Cherubini, E. (2004). GABA-mediated giant depolarizing potentials as coincidence detectors for enhancing synaptic efficacy in the developing hippocampus. Proc. Natl. Acad. Sci. USA *101*, 3967–3972.

93. Roux, L., Hu, B., Eichler, R., Stark, E., and Buzsáki, G. (2017). Sharp wave ripples during learning stabilize the hippocampal spatial map. Nat. Neurosci. *20*, 845–853.

94. Oliva, A., Fernández-Ruiz, A., Buzsáki, G., and Berényi, A. (2016). Role of hippocampal CA2 region in triggering sharp-wave ripples. Neuron *91*, 1342–1355.

95. Hulse, B.K., Moreaux, L.C., Lubenov, E.V., and Siapas, A.G. (2016). Membrane potential dynamics of CA1 pyramidal neurons during hippocampal ripples in awake mice. Neuron *89*, 800–813.

96. Papatheodoropoulos, C. (2010). Patterned activation of hippocampal network (approximately 10 Hz) during in vitro sharp wave-ripples. Neuroscience *168*, 429–442.

97. Zheng, K.Y., Jensen, T.P., and Rusakov, D.A. (2018). Monitoring intracellular nanomolar calcium using fluorescence lifetime imaging. Nat. Protoc. *13*, 581–597.

98. Madisen, L., Mao, T., Koch, H., Zhuo, J.M., Berenyi, A., Fujisawa, S., Hsu, Y.W., Garcia, A.J., 3rd, Gu, X., Zanella, S., et al. (2012). A toolbox of Cre-dependent optogenetic transgenic mice for light-induced activation and silencing. Nat. Neurosci. *15*, 793–802.

99. Kopell, N., Borgers, C., Pervouchine, D., Malerba, P., and Tort, A. (2010). Gamma and theta rhythms in biophysical models of hippocampal circuits. In Hippocampal Microcircuits (Springer), pp. 423–457.

100. Tort, A.B.L., Rotstein, H.G., Dugladze, T., Gloveli, T., and Kopell, N.J. (2007). On the formation of gamma-coherent cell assemblies by oriens lacunosum-moleculare interneurons in the hippocampus. Proc. Natl. Acad. Sci. USA *104*, 13490–13495.

101. Gloveli, T., Dugladze, T., Rotstein, H.G., Traub, R.D., Monyer, H., Heinemann, U., Whittington, M.A., and Kopell, N.J. (2005). Orthogonal arrangement of rhythm-generating microcircuits in the hippocampus. Proc. Natl. Acad. Sci. USA *102*, 13295–13300.

STAR★METHODS

KEY RESOURCES TABLE

REAGENT or RESOURCE	SOURCE	IDENTIFIER
Bacterial and virus strains		
AAV2/9-syn-iGABASnFR2-WPRE	GENIE Project Team & Looger Lab	This Paper
AAV2/9-GFAP-iGABASnFR2-WPRE	GENIE Project Team & Looger Lab	This Paper
Biological samples		
Sprague Dawley Rats Postnatal Day 5-8 (Organotypics)	Envigo	RRID: MGI:5651135
C57BL/6 Mice Postnatal Day 5-8 (Organotypics)	Envigo	RRID: MGI:2159769
PV::cre mice (B6;129P2-Pvalb$^{tm1(cre)Arbr}$/J) (Acute slices)	Jackson laboratory	RRID: IMSR_JAX:008069
Mice floxed-stop tdTomato (B6.Cg-Gt(ROSA)26Sortm9 (CAG-tdTomato)Hze/J) (acute slices)	Jackson laboratory	RRID: IMSR_JAX: 007909
Mice cre-dependent EYFP-tagged excitatory opsin channelrhodopsin-2-H134R (B6;129S-Gt(ROSA)26Sortm32 (CAG-COP4*H134R/EYFP)Hze/J) (acutes slices)	Jackson laboratory	RRID: IMSR_JAX: 012569
Chemicals, peptides, and recombinant proteins		
Minimal Essential Media	Thermofisher	Cat # 21090-022
HBSS	Thermofisher	Cat # 14185-045
B27	Thermo Fisher	Cat # 12587010
HEPES solution	Merck	Cat # H0887-20ML
Penicillin/Streptomycin	Thermofisher	Cat # 15070063
Ascorbic Acid	Merck	Cat # A5960-25G
Glucose	Merck	Cat # G8270-100G
Horse Serum	Merck	Cat # H1270-100ML
Molecular Probes Alexa Fluor 488 Hydrazide	Fischer Scientific	Cat# 10296832
NO-711	Biotechne/Tocris	Cat# 1779
NBQX disodium salt	Biotechne/Tocris	Cat# 1044/1
D-AP5	Biotechne/Tocris	Cat# 0106
Picrotoxin	Biotechne/Tocris	Cat# 1128
CGP 52432	Biotechne/Tocris	Cat# 1246
GABA	Merck	Cat # A5835-10G
Experimental models: Organisms/strains		
Sprague Dawley Rats	Envigo	RRID: MGI:5651135
C57BL/6 Mice	Envigo	RRID: MGI:2159769
PV::cre mice (B6;129P2-Pvalb$^{tm1(cre)Arbr}$/J)	Jackson laboratory	RRID: IMSR_JAX:008069
Mice floxed-stop tdTomato (B6.Cg-Gt(ROSA)26Sortm9 (CAG-tdTomato)Hze/J)	Jackson laboratory	RRID: IMSR_JAX: 007909
Mice cre-dependent EYFP-tagged excitatory opsin channelrhodopsin-2-H134R (B6;129S-Gt(ROSA) 26Sortm32(CAG-COP4*H134R/EYFP)Hze/J)	Jackson laboratory	RRID: IMSR_JAX: 012569
Recombinant DNA		
pAAV.hSynap.iGABASnFR	Loren Looger Lab	RRID: Addgene_112159
pAAV.hSynap.iGABASnFR2	GENIE Project team/ Looger Lab	This Paper
Software and algorithms		
MES v4.x-v.6.3	Femontics	RRID: SCR_018309
MESc 3.57	Femontics	https://femtonics.eu/femtosmart-software/
Axon PClamp	Molecular Devices	RRID: SCR_011323

(*Continued on next page*)

Continued		
REAGENT or RESOURCE	SOURCE	IDENTIFIER
MATLAB	Mathworks	RRID: SCR_001622
Fluorescent Imaging Analysis Software (FIMAS)	https://github.com/zhengkaiyu/FIMAS	RRID: SCR_018311
Python Programming Language	Python, Anaconda	RRID: SCR_008394
WinEDR, Strathclyde Electrophysiology Software	Strathclyde University	N/A
OriginPro	OriginLab	RRID: SCR_014212
Network simulation software	In-house	https://github.com/LeonidSavtchenko/Arachne
Other		
Multiclamp 700B	Molecular Devices	RRID: SCR_018455
Digidata 1550	Molecular Devices	Digidata 1550
Femto3D-RC Multiphoton scanning microscope	Femontics	Femto3D-RC
Femto2D Multiphoton scanning microscope	Femontics	Femto2D
Femtosmart microscope	Femontics	Femtosmart
Leica VT1200S vibrating microtome	Leica BioSystems	RRID: SCR_020243
BioRad Helios Gene Delivery System	Bio Rad	RRID: SCR_019723
pE-2 LED illumination system	CoolLED	N/A
Slicescope Pro 2000	Scientifica	RRID: SCR_018405
Patchstar Manipulator	Scientifica	N/A

RESOURCE AVAILABILITY

Lead contact

Further information and requests for resources and reagents should be directed to and will be fulfilled by the lead contact, Dmitri Rusakov (d.rusakov@ucl.ac.uk).

Materials availability

This study did not generate new unique reagents.

Data and code availability

- Data reported in this paper will be shared by the lead contact upon request.
- The original code for network simulation is deposited at GitHub and is publicly available as of the date of publication. Doi is specified in the key resources table.
- Any additional information required to reanalyze the data reported in this paper is available from the lead upon request.

EXPERIMENTAL MODEL AND SUBJECT DETAILS

Animal experimentation

All experiments involving animals were carried out in accordance with the European Commission Directive (86/609/EEC) and the United Kingdom Home Office (Scientific Procedures) Act (1986), under the Home Office Project Licence PPL P2E0141 E1.

Experimental model designs across preparations

In our experimental protocols, we attempted to deviate from the 'normal' physiological conditions as little as possible, to induce similar types of epileptiform activity, rather than reproducing exactly the same protocol that could lead to distinct phenomena depending on the preparation. For instance, we used 0 mM [Mg^{2+}] and 5 mM [K^+] with no glutamatergic transmission blockade to induce interictal spikes, but had to increase [K^+] to 10 mM to induce GABA waves with glutamatergic transmission blocked. In organotypic slices. 0 mM [Mg^{2+}] was sufficient to trigger interictal events without the need to raise [K^+], as further detailed below. To focus on a stable spiking regime, under equilibrated [K^+], a brief period of several initial spikes was not considered for analyses.

Acute slice preparation

In vitro electrophysiological recordings were performed in acute hippocampal slices prepared from 3- to 4-week-old male Sprague-Dawley rats (Harlan Laboratories Inc, Bicester, UK). Animals were kept under standard housing conditions with

12h : 12h light-dark cycle and free access to food pellets and drinking water. After being sacrificed using an overdose of isoflurane, animals were decapitated, brains were rapidly removed, and hippocampi were dissected for slice preparation. Transverse hippocampal slices (350 μm-thick) were cut with a Leica VT1200S vibratome (Germany) in an ice-cold sucrose-based solution containing (in mM): sucrose (70), NaCl (80), KCl (2.5), $MgCl_2$ (7), $CaCl_2$ (0.5), $NaHCO_3$ (25), NaH_2PO_4 (1.25), glucose (22), bubbled continuously with 95% O_2 + 5% CO_2 to yield a pH of 7.4. Slices were allowed to recover in a sucrose-free artificial cerebrospinal fluid (aCSF) solution (in mM): NaCl (119), KCl (2.5), $MgSO_4$ (1.3), $CaCl_2$ (2.5), $NaHCO_3$ (26.2), NaH_2PO_4 (1), glucose (22), bubbled with 95% O_2 and 5% CO_2 in an interface chamber for at least 1 hour at room temperature before being transferred to a submerged recording chamber.

Modified aCSF was used to induce epileptiform activity: nominally 0 mM Mg^{2+} and 5 mM / 10 mM K^+ under intact / blocked glutamatergic transmission, as specified. To facilitate the rapid generation of epileptiform discharges slices were perfused with the solution on both sides. All recordings were done at 32°C. $GABA_B$ receptors were blocked in all experiments by 5 μM CGP52432. Field potential recordings from *stratum pyramidale* were performed with 1-2 MΩ glass electrodes filled with aCSF. Visualized whole-cell voltage-clamp recordings were performed from CA1 pyramidal neurons using an infrared differential interference contrast imaging system. Recording pipettes (3-5 MΩ) were filled with internal solution, containing (in mM): Cs-methanesulfonate (120), HEPES (10), EGTA (0.2), NaCl (8), $MgCl_2$ (0.2), Mg-ATP (2), Na-GTP (0.3), QX-314 Br^- salt (5), pH 7.2, osmolarity 290 mOsm kg^{-1}. $GABA_A$R-mediated currents were recorded from neurons voltage clamped at 0 mV or +10 mV (close to the reversal potential of glutamatergic currents). To further prevent the contribution of NMDA receptors MK801 (1 μM) was included in the recording pipettes in acute slices experiments. CA3 area was cut off in the outside-out patch experiments, but not in other settings: this had no detectable effect on interneuronal network activity in area CA1.

Series resistance (R_s) was monitored throughout the experiment using a –5 mV step command. Cells showing a >20% change in R_s, or values >25 MΩ, or an unstable holding current, were rejected. Recordings were obtained using a MultiClamp 700B amplifier (Molecular Devices, CA, USA), filtered at 4 kHz, digitized and sampled through an AD converter Digidata 1550 (Molecular Devices) or NI PCI-6221M (National Instruments) at 10 kHz and stored on a PC. Data acquisition and off-line analysis were performed using WinEDR 3.0.1 (University of Strathclyde, Glasgow, UK) and Clampfit 10.0 (Molecular Devices Corporation, USA) software, or Femtonics MES (Femtonics, Budapest) and custom-made MATLAB (Mathworks) scripts running in MATLAB r2020b.

Organotypic slice culture preparation and biolistic transfection of iGABASnFR2

Organotypic hippocampal slice cultures were prepared and grown with modifications to the interface culture method from P6–8 Sprague-Dawley rats as previously described[48] using the Stoppini interface method, which ensures no significant spontaneous activity in baseline conditions. In brief, 300 μm thick, isolated hippocampal brain slices were sectioned using a Leica VT1200S vibratome in ice-cold sterile slicing solution consisting of (in mM) Sucrose 105, NaCl 50, KCl 2.5, NaH_2PO_4 1.25, $MgCl_2$ 7, $CaCl_2$ 0.5, Ascorbic acid 1.3, Sodium pyruvate 3, $NaHCO_3$ 26 and Glucose 10. Following washes in culture media consisting of 50% Minimal Essential Media, 25% Horse Serum, 25% Hanks Balanced Salt solution, 0.5% L-Glutamine, 28 mM Glucose and the antibiotics penicillin (100U/ml) and streptomycin (100 μg/ml), three to four slices were transferred onto each 0.4 μm pore membrane insert (Millicell-CM, Millipore, UK). Cultures were then maintained at 37°C in 5% CO_2 and fed by medium exchange every 2-3 days for a maximum of 21 days in vitro (DIV). At 5DIV cultures were treated overnight with 5 μM cytosine arabinoside (Ara-C, a poison inhibitor of DNA and RNA polymerases) to reduce glial reaction following biolistic transfection and returned to standard culture media at 6DIV. At 8DIV cultures were shot with 1.6 micron Gold micro-carriers coated with 30 μg of hSyn-iGABASnFR2 plasmid using the Helios gene-gun system (Bio-Rad). Following transfection cultures remained for 5-10 days before experiments were carried out. In this preparation, modified aCSF with 0 [Mg^{2+}] (without raised [K^+]) was sufficient to trigger interictal activity.

METHOD DETAILS

Viral transduction of iGABASnFR2 for acute slice experiments

We used viral transduction of the optical GABA sensor in C57BL/6J mice (Charles River Laboratories), as detailed earlier, either in neonatal pups[47] or in adult animals.[49] Briefly, an AAV virus expressing the optical GABA sensor, iGABASnFR2 (Janelia Research Campus), under either neuronal (*hSyn*) or astroglial (*GFAP*) promoter, as indicated, was injected into the cerebral ventricles of neonates (P0-P3 of either sex) during aseptic surgery. The viral particles were injected at ∼1.5-2 μl per hemisphere (3-5 x 10^9 genomic copies in total), at a rate not exceeding 0.2 μl/s, guided to a location approximately 0.25 mm lateral to the sagittal suture and 0.50–0.75 mm rostral to the neonatal coronary suture and 2 mm ventral from the surface of the cranium. After pups received AAV injections, they were returned to the mother in their home cage. The animals were systematically kept as a group of litters and monitored for several days thereafter to ensure that no detrimental effects appeared. Imaging experiments were performed 3 to 5 weeks after the injections, at which point acute hippocampal slices were prepared, as described above.

For the viral transduction of iGABASnFR2 in adults, young C57BL/6J mice (1–2-month-old), male and female, were prepared for aseptic surgery and anesthetized with isoflurane (3.5-4% v/v induction). The scalp was shaved and disinfected using topical chlorhexidine; topical lidocaine/prilocaine emulsion (2.5%) and ocular ointment (Lacri-lube, Allergan, UK) were applied. The animal was secured in a stereotaxic frame, and a stable anesthesia level (maintenance at 1.5-2.5% isoflurane) was confirmed before starting surgery. Body temperature was maintained at ∼37.0°C using a feedback rectal thermometer and heating blanket. Perioperative analgesics were administered (subcutaneous buprenorphine, 60 μg kg^{-1}). A small, vertical incision was made to expose the skull. A craniotomy of approximately 1 mm diameter was performed over the right hemisphere using a high-speed drill, at a site overlying

the medial hippocampus. Stereotactic coordinates were 2.5 mm of the anteroposterior distance from bregma to lambda and 2.5 mm lateral to the midline. Pressure injections of AAV9-hsyn-iGABASnFR2-WPRE or AAV9-GFAP-iGABASnFR2-WPRE (totaling 1×10^{10} genomic copies in a volume not exceeding 200 nL) were carried out using a Hamilton syringe needle stereotactically guided to a depth of 2.6 mm ventral from the cortical surface, at a rate of approximately 1 nL sec^{-1}. The total injection volume was delivered in two steps, reducing the depth by 200 μm. Once delivery was completed, the needle was left in place for 5-7 minutes before being retracted. The surgical wound was closed with absorbable 6-0 sutures. Metacam (1 mg kg^{-1}) and saline (0.5 ml) were subcutaneously administered, and the animal was left to recover in a heated chamber. Imaging experiments were performed 3 to 5 weeks after the injections. The procedure of cardiac perfusion of animals was performed under general anesthesia, using an ice-cold aCSF saturated with 95% O_2 and 5% CO_2, before dissecting the brain for the acute hippocampal slice preparations.

Outside-out patch recordings

Single channel $GABA_AR$-mediated currents were recorded in the presence of 0.1 μM CGP-55845 as detailed earlier.[18,38] Outside-out patches were pulled from dentate granule cells, raised above the slice surface and moved to area CA1; recordings were performed in voltage-clamp mode at 33–35°C. For membrane patches held at -70 mV, the recording electrode solution contained (in mM): 120.5 CsCl, 10 KOH-HEPES, 10 BAPTA, 8 NaCl, 5 QX-314-Br^- salt, 2 Mg-ATP, 0.3 Na-GTP; pH 7.2, 295 mOsm kg^{-1}. For membrane patches held at 0 mV, the recording electrode solution contained (in mM): 120 Cs-methanesulfonate, 10 HEPES, 0.2 EGTA, 8 NaCl, 0.2 $MgCl_2$, 2 Mg-ATP, 0.3 Na-GTP, 5 QX-314-Br^- salt; pH 7.2, 290 mOsm kg^{-1}. Recordings were made using MultiClamp 700B amplifier (Molecular Devices, CA, USA); signals were digitized at 10 kHz. The patch pipette resistance was 5–7 MΩ. In experiments where GABA EC_{50} was determined, we used a θ-glass application pipette with ~200 μm tip diameter attached to a micromanipulator. The pipette position was controlled by a piezoelectric element (each switch took 50–100 μs) to allow rapid solution exchange. One pipette channel was filled with the bath aCSF solution; another channel had aCSF with varying concentrations of GABA. The flow rate was driven by gravity.[44]

Analysis of single-channel recordings and iGABASnFR2 titration

The frequency of $GABA_AR$ channel openings was calculated as $N / \Delta t$, where N is the number of openings and Δt is the time of recording. N was counted using a detection threshold of 1.5 pA above the mean baseline value and a minimum opening time of >0.2 ms. Single channel conductance was calculated as $G = I / (V_{rev} - V_{hold})$, where I stands for receptor-mediated current, and V_{rev} – calculated chloride reversal potential. The average charge transfer through receptors was calculated as $Q = G \times N / \Delta t$. The concentration-effect plot was fitted with the Hill equation $E = \frac{[C]^n}{K_d^n + [C]^n}$, where E stands for the percentage of maximal charge transfer, C concentration of GABA, K_d microscopic dissociation constant (equal to EC_{50}), and n Hill's coefficient. A similar equation was applied to obtain the best-fit function for the iGABASnFR2 calibration (Figure 2D).

Two-photon excitation imaging

For imaging of iGABASnFR2 transients associated with interictal activity, organotypic slice cultures were cut from their membrane insert and transferred to the stage of a Femtonics Femto3D-RC, Femto-2D, or in some experiments, Femtosmart imaging system (Femtonics, Budapest) integrated with patch-clamp electrophysiology and linked on the same light path to two femtosecond pulse lasers MaiTai or Insight X3, respectively (SpectraPhysics-Newport) with independent shutter and intensity control as previously described.[48] Imaging settings were adjusted to provide optimal recording conditions for the preparation. Both imaging and electrophysiology recordings were acquired from the microscope acquisition board at 40kHz; electrophysiology data were then decimated to 10kHz and data from the detectors processed by the scope to form frame scans at 10-50Hz. Normally, as the imaging frame rate is lower than the field potential acquisition rate, we sub-divide the imaging bins into equivalent-value bins to match the electrophysiology recording bins, and thus to align the two recordings accurately. Laser light intensity under the objective was adjusted to minimize photobleaching (<7 mW), at a wavelength of $\lambda_x^{2p} = 910$ nm; control for photobleaching, pixel and optical resolution, were set and optimised as detailed previously.[48,97] The iGABASnFR2 sensitivity was several-fold that of iGABASnFR[36] providing a stable readout of resting (equilibrated) GABA-sensitive fluorescence between interictal spikes (Figure 2B): the F_0 value was measured as the average baseline intensity over a ~1 s time window.

Slices were continuously perfused with bicarbonate based artificial cerebrospinal fluid (aCSF) equilibrated with 95% O_2 and 5% CO_2 at 32–34 °C using a gravity driven perfusion system (flow rate 3-4ml/min). aCSF solution contained (in mM): 120 NaCl, 10 glucose, 2.5 KCl, 1.3 $MgSO_4$, 1 NaH_2PO_4, 25 $NaHCO_3$, 2 $CaCl_2$ with osmolarity of 300 ± 5 mOsm kg^{-1}. Biolistic transfection of iGABASnFR2 resulted in sparse labeling of neurons in the CA1 and CA3 regions making identification of iGABASnFR2 expressing cells using two-photon imaging ($\lambda_x^{2p} = 910$ nm) unambiguous. Following identification, a glass field recording electrode filled with standard aCSF was placed within ~30 μm of the cell of interest and interictal activity was then initiated by exchanging the extracellular solution for a 0 mM Mg^{2+} aCSF (otherwise the same composition).

The local field potential was then monitored until stable interictal bursting was observed, at which point curved frame-scan regions of interest following the somatic membrane were chosen for scanning (see Figure 2A for illustration). To avoid photodamage, scan duration was limited to 9 second epochs which were repeated with a one-minute intervals until a minimum of 10 events were captured. For comparison of interictal event timing and associated iGABASnFR2 transients; interictal event peak times were first identified by the *findpeaks* function in MATLAB and were accepted for further analysis where single peaks with a minimum width

CellPress
OPEN ACCESS

of 20ms and amplitude >5 x standard deviation (SD) of baseline noise could be detected. The associated iGABASnFR2 $\Delta F/F_0$ peak was then identified (again using *findpeaks*) within a manually adjusted window -750 ms to + 2s (extremes between cells/slices) over the peak times identified for the interictal event, with a minimum >2.5 x SD baseline and minimum width of 50ms. Events within that window were then analyzed where a clear and stable iGABASnFR $\Delta F/F_0$ 50ms length baseline, rise and visible decay associated could be observed. The rise to peak from baseline was then fitted by polynomial regression and the 20% iGABASnFR2 peak time extrapolated from the fit and used to calculate iGABASnFR transient-interictal event lag times.

Targeted cell-attached recordings and optogenetic experiments

PV-Cre mice (B6;129P2-Pvalb$^{tm1(cre)Arbr}$/J Jackson laboratory stock number: 008069) were crossed with Ai9 or Ai32 mouse line, which has floxed-stop tdTomato (B6.Cg-Gt(ROSA)26Sortm9(CAG-tdTomato)Hze/J Jackson laboratory stock number: 007909) or EYFP-tagged excitatory opsin channelrhodopsin-2-H134R (B6;129S-Gt(ROSA)26Sor$^{tm32(CAG-COP4*H134R/EYFP)Hze}$/J Jackson laboratory stock number: 012569), to produce animals expressing either tdTomato or channelrhodopsin-2 (ChR2) in parvalbumin-positive (PV+) interneurons throughout the brain.[98] Animals were kept under standard housing conditions with 12 h: 12 h light-dark cycle and free access to food pellets and drinking water. Hippocampal slices were prepared from mice of both sexes aged between postnatal day 25 and 50 for the electrophysiology section, but under low light intensity to minimize any adverse effect due to activation of ChR2. Field potential recordings from the *stratum pyramidale* and visualized whole-cell voltage-clamp recordings were performed from CA1 pyramidal neurons using infrared differential interference contrast imaging system as in the electrophysiology section. For the dual cell-attached recordings (Figure 5), borosilicate glass pipettes (3-5 MΩ) filled with aCSF and containing Alexa Fluor 488 (50 μM) were used and the whole-cell recording pipettes was containing the standard Cs-methanesulfonate cited above with the addition of Alexa Fluor 488. In addition, the slices were superfused with aCSF with 0 mM Mg^{2+} and 10 mM K^{+} as well as APV (50 μM), NBQX (10 μM) and CGP52432 (1 μM); the $GABA_AR$ blocker picrotoxin (100 μM) was added where indicated.

Cells showing a series resistance >25 MΩ, or an unstable holding current, were rejected. Recordings were obtained using a MultiClamp 700B amplifier (Molecular Devices, CA, USA), filtered at 2-4 kHz and digitized at 10 kHz. Data acquisition and off-line analysis were performed using WinEDR 3.0.1 (University of Strathclyde, Glasgow, UK) and Clampfit 10.0 (Molecular Devices Corporation, USA) software as well as MATLAB and Python custom-scripts.

Wide-field illumination of the CA1 region of the hippocampus was delivered through a 20x water immersion objective (Olympus). PV tdTomato-positive cells were visualized with 590 nm wavelength and ChR2 was activated by blue light (wavelength 470 nm) generated by pE-2 LED illumination system (CoolLED); light intensity under the objective was in the range of 5 - 10 mW. For the ramp stimulation protocol, a train of 1 s ramps was delivered at on average ~0.3 Hz ranging from 0.12 to 0.33 Hz.

In some experiments (Figure 4), neurons showing the 'wave-like' activity were defined as follows. The voltage clamp trace was lowpass filtered with an IIR Butterworth filter at a cut-off frequency of 2Hz. The FFT was then computed on the filtered trace and if a significant (above 95CI) peak was present on the power spectrum between 0.01-2Hz, the activity was considered as wave-like.

Interictal discharge analysis

The detection of interictal spikes was done using custom-made python script. Local field electrophysiological trace was first downsampled to 1 kHz and filtered using a Butterworth bandpass filter from 1 to 50 Hz. The onset of interictal discharges was determined by using a detection threshold on the second derivative of the local field potential recording. The threshold corresponded to a minimum of 4 SDs above baseline noise (taking from the beginning of each trace). For the probability of inducing an interictal events with 1 ms light pulse stimulation, a window of 100 ms post-stimulation was considered for the detection of an event. Concerning the ramp stimulation, the window was between 500 ms after the start of the ramp and 100 ms post-stimulation.

Modeling: Networks of interneurons and pyramidal neurons

The interneurons and pyramidal neuron (principal cell, PC) networks were simulated on a digital platform ARACHNE with remotely-controlled parallel computing.[41] Similar to the previously explored non-hierarchical networks,[15] the present one featured circular connectivity (Figure 3A), which helped (a) exclude edge effects, (b) equalize cell contribution, and (c) represent the size by a single parameter, network radius R. In individual simulated cells, the channel kinetics were typical of hippocampal fast-spiking basket interneurons.[83] Other incorporated biophysical mechanisms, such as ion channel flux, GABA transporter, synaptic and GABA tonic current were in accord with experimental findings,[99–101] as detailed below. The time course of GABAergic synaptic conductance followed the function $y(t) = G_{ii}(\exp(-t/\tau_1) - \exp(-t/\tau_2))$ where $y(t)$ is the synaptic conductance at time t, τ_1 is the rise time constant, τ_2 (termed tau elsewhere) is the decay time constant, G_{ii} is the peak conductivity (mS cm^{-2}) of synapses.

A network of interneurons and pyramidal cells was simulated using previously published models,[99,100] which was further developed as a full-scale cloud-computing platform ARACHNE,[41] with minor modifications of the internal parameters. The critical addition was the dynamic G_{tonic}, as explained below, with the G_{tonic} range in interneurons twice that of pyramidal cells, in accord with experimental data[15]; the basic cell-circuit model used in the present study was obtained from ModelDB (accession no. 138421). Briefly, the modelled circular network consisted of N fast-spiking interneurons (I-cells) and M pyramidal cells (E-cells). Each cell was modelled as a single compartment using standard Hodgkin–Huxley formalism. The $GABA_AR$ reversal potential V_{GABA} was set between –55 mV and –72 mV, as specified. Unless specified otherwise, the network featured three connection types: *I–I*, *I–E*, and *E–I*, with release probabilities values P_{E-E} = 0.5, P_{E-I} = 0.5, and P_{I-I} = 1.0, respectively. The spatial synaptic connection density was defined as the matrix W_{XY} (i,j) with the Gaussian distribution centered at a given neuron, so that

$$W_{XY}(i,j) = W_{XY\max} \exp\left(-\frac{1}{2}\left(\frac{\max(i,j) - 1}{\sigma_{xy}}\right)^2 \left(\frac{j-1}{\max(j)} - \frac{i-1}{\max(i)}\right)^2 \right)$$

The model consists in bell-shaped strength and Gaussian density of connections. Synaptic conductance matrices of this model are with following strength coefficient $W_{ee,\max} = 2$, $W_{ii,\max} = 0.8$, $W_{ei,\max} = 0.9$; $W_{ie,\max} = 0.3$, and spatial SD: $\sigma_{ee} = 10$ μm, $\sigma_{ei} = 12.5$ μm, $\sigma_{ie} = 8$ μm and $\sigma_{ii} = 5$ μm. The size (radius) of interneuron and principal cell network was set at 250 and 200 μm, respectively, with the signal propagation speed at 0.1 μm ms^{-1}.

Simulations were performed using ARACHNE on the Amazon AWS cloud computing (cluster c5.large, tolerance 10^{-5}, time step 0.02 ms). Random generator *use32BitRng* (MATLAB) was set to generate a delta correlated white noise for any stochastic processes. The initial voltage of interneurons in the network was set uniformly randomly, between -73 and -67 mV. Mechanisms of synaptic plasticity were excluded from the basic model of the neural network.

Modeling: Network synchronization

The network synchronization parameter $k(\tau)$, which was also used for the experiment in PV+ cell pairs (Figure 4), was calculated as an average of all coefficients $k_{i,j}(\tau)$ for each pair of neurons (*i, j*). The time window of synchronization was divided into bins so that $\tau = 0.1/f_m$ where f_m is the network-average spiking frequency (see below). In each bin, an action potential was represented in a binary format (yes-no series). Next, $k_{i,j}(\tau)$ was calculated for each pair of neurons for the time bin τ using the formula:

$$k_{ij}(\tau) = \frac{\sum_{l=1}^{K} X(l)Y(l)}{\sqrt{\sum_{l=1}^{K} X(l) \sum_{l=1}^{K} Y(l)}}$$

where *X* and *Y* are the binary series of the *i*th and *j*th cells, respectively, *l* is the bin number; thus, *X(l)* and *Y(l)* are either 0 or 1 depending on having a spike (1) or no spike (0) event for *i*th or *j*th during the *l*th bin, and *K* is the total number of bins.

Modeling: Tonic GABA conductance

$[GABA]_e$ activates extrasynaptic $GABA_A$Rs, which generate G_{tonic}. Thus, modelled cells generate non-specific tonic current: $I_{tonic} = G_{tonic}(V_{GABA} - V_m)$, where V_{GABA} is $GABA_A$R reversal potential, and V_m is the membrane potential. G_{tonic} depends on the interneuron network firing frequency, which in turn generates $[GABA]_e$ increments, in accord with:

$$\frac{dG_{tonic}}{dt} = N_i A_f (f_m[t - dt] + f_b) - G_{pump}(G_{tonic} - G_{basal}) \quad \text{(Equation 1)}$$

with initial $G_{tonic}(0) = G_{basal}$ and f_m is calculated as the total of cell-generated spikes over time *T*:

$$f_m = \frac{1}{T\ N_i} \sum_{i=1}^{N_i} AP_i(T,t) \quad \text{(Equation 2)}$$

Other parameters were: *N* (or N_i), total number of neurons in the network; A_f (0.01 nS cm^{-2} ms^{-1}, unless specified otherwise), tonic GABA conductance resulting from a single AP over 100 ms; G_{pump} (values as indicated), the GABA uptake rate, $G_{basal} = 0.1$ mS cm^{-2}, $[GABA]_e$ value at which GABA transporters reverse, $f_b = 0.1$ Hz, basal average network spiking frequency at rest; and *dt*, a delay between neurotransmitter release and activation of extrasynaptic receptors (*dt* was <1 ms throughout).

In our simulations, we assumed that G_{tonic} linearly depends on $[GABA]_e$: $G_{tonic} = \alpha[GABA]_e$. This assumption provides a good approximation $[GABA]_e < 1$-2 μM, as in this case it falls into the near-linear approximation of the charge transfer versus $[GABA]_e$ relationship (Figure 1E). The G_{tonic} dynamics was therefore determined by the time course of $[GABA]_e$, which is represented by quasi-instantaneous release followed by uptake (i.e., quasi-exponential decay of $[GABA]_e$ to its basal level). In the basic established model, the GABA transport reversal potential V_{GAT} is given by

$$V_{GAT} = \frac{RT}{F}\left(2\,Ln\frac{[Na]_{out}}{[Na]_{in}} + Ln\frac{[GABA]_{out}}{[GABA]_{in}} - Ln\frac{[Cl]_{out}}{[Cl]_{in}}\right),$$

which was explored previously, together with the estimates of the GAT-1 kinetics, in some detail,[54] so that the solution of equation (1) for constant f_m was

$$G_{tonic}(t) = e^{-t_p\,t}G_{basal} + \frac{A_f f_m N_i}{t_p} e^{-t_p\,t}(e^{t_p\,t} - 1),$$

where t_p is 0.02–0.1 ms^{-1} and A_f is the G_{basal} / G_{tonic} ratio at steady-state for a given frequency f_m: $A_f = \frac{KG_{init}t_p}{f_m N_i}$, where $2 < K < 4$. Thus, A_f was a scaling factor for $[GABA]_e$ action.

Modeling: $[GABA]_e$ dynamics

The dynamics of $[GABA]_e$ was estimated from the cell-spiking raster plot, with individual action potentials releasing GABA at a rate of 0.5 μM ms^{-1} of GABA (~3000 molecules) in an extracellular volume of 20 μm radius, the average distance between neighbouring neurons of the network. $[GABA]_e$ decays due to GABA uptake and diffusion, a typical constant of 0.004 μM ms^{-1} (Savtchenko et al.[54]), unless specified otherwise.

To explore the parameter space with respect to rhythm generation, and for the sake of clarity, we introduced a single dimensionless parameter R as a combined GABA release-uptake factor incorporating A_f and G_{pump}, in accord with

$$\frac{d[GABA]_e}{dt} = \frac{G_{tonic}}{dt} = N_i \cdot R \cdot A_f(f_m[t-dt] + f_b) - R \cdot G_{pump}(G_{tonic} - G_{basal})$$

were term notation is as described above.

Modeling: Code detail

ARACHNE is available with explanatory documentation at https://www.neuroalgebra.com/. The program is made available under MIT license. ARACHNE is written in a way that allows users to run it on any remote platforms. Files with a set of initial parameters for reproducing the results compatible with ARACHNE can be provided upon request. For any computations we provided the exe file of ARACHNE, but also the initial code on https://github.com/LeonidSavtchenko/Arachne.

QUANTIFICATION AND STATISTICAL ANALYSIS

Shapiro-Wilk tests for normality were routinely run for small samples (this test for the means could be misleading for $n > 15$-19 due to Central Limit Theorem). Correspondingly, two-tailed paired and unpaired Student's t-test, or otherwise non-parametric Mann-Whitney tests were used for statistical analyses. Mean difference was considered significant at the null-hypothesis rejection level of $p < 0.05$. Statistical summary data are shown as mean ± SEM unless specified otherwise. To account for the factors of slices versus recorded cell pairs, a Kruskal-Wallis non-parametric ANOVA was performed.

Current Biology, Volume *33*

Supplemental Information

Volume-transmitted GABA waves pace epileptiform rhythms in the hippocampal network

Vincent Magloire, Leonid P. Savtchenko, Thomas P. Jensen, Sergyi Sylantyev, Olga Kopach, Nicholas Cole, Olga Tyurikova, Dimitri M. Kullmann, Matthew C. Walker, Jonathan S. Marvin, Loren L. Looger, Jeremy P. Hasseman, Ilya Kolb, Ivan Pavlov, and Dmitri A. Rusakov

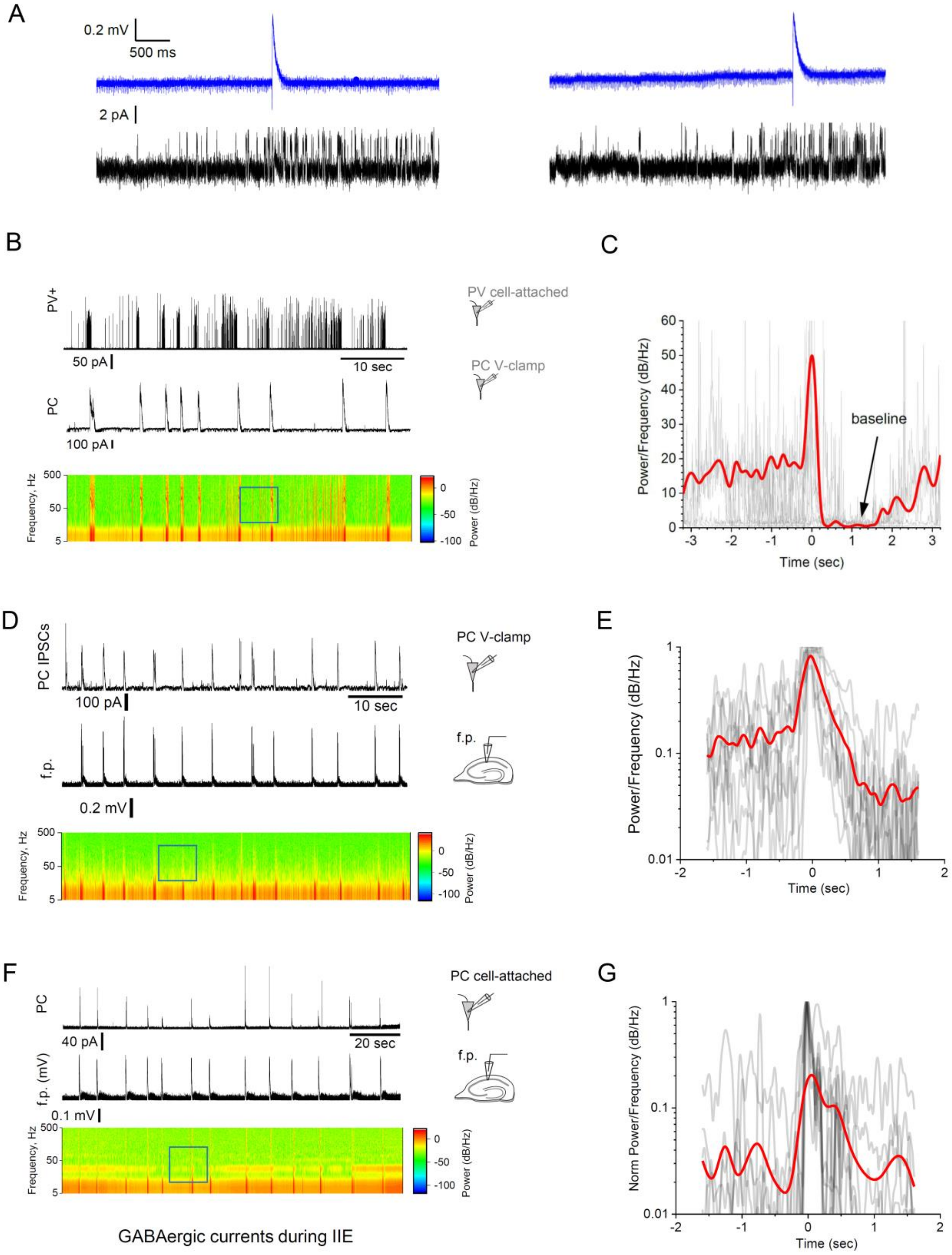

Figure S1. FS PV+ interneurons increase firing rate before inter-ictal events, Related to Figure 1.

(A) Examples of local field potentials (f.p., top trace) and sniffer-patch single-channel activity (bottom) before and after individual interictal discharges in hippocampal slices.

(B) Simultaneous cell-attached spiking recording in a FS PV+ interneuron (top) and whole-cell recording of $GABA_AR$ IPSCs in a pyramidal neuron (middle), with the power spectrum density of interneuronal spiking (bottom). Note that our analysis did not isolate IPSCs, it computed the Fourier transform of the continuous IPSC signal for a wide range of frequencies (the Wavelet Transform, 5-500 Hz) and then averaged the fast-spiking parts of the Wavelet pattern (10-200 Hz) centred at the pyramidal cell current peak onset; blue box, the area of averaging.

(C) Average power density across the spectrum, over the typical cycle of interictal spiking, centred at burst peak (blue square in B); red line, sample average; grey, individual interictal spike traces (n = 10, alignment at field potential spike onset, $t = 0$); arrow, post-spike returns to baseline, before the rise of spiking activity preceding an IIE.

(D) Simultaneous recordings of $GABA_AR$ IPSCs in pyramidal cells (top) and local f.p. (middle), the power spectrum density of IPSCs recorded in pyramidal cells (bottom).

(E) Graph as as in C, but for pyramidal cell spiking shown in D (n = 9).

(F) Simultaneous cell-attached recordings of a pyramidal neuron (top), and local field potential (f.p.) during interictal events, with the power spectrum density of pyramidal cell spiking (bottom).

(G) Graph as as in C, but for pyramidal cell spiking shown in F (n = 10).

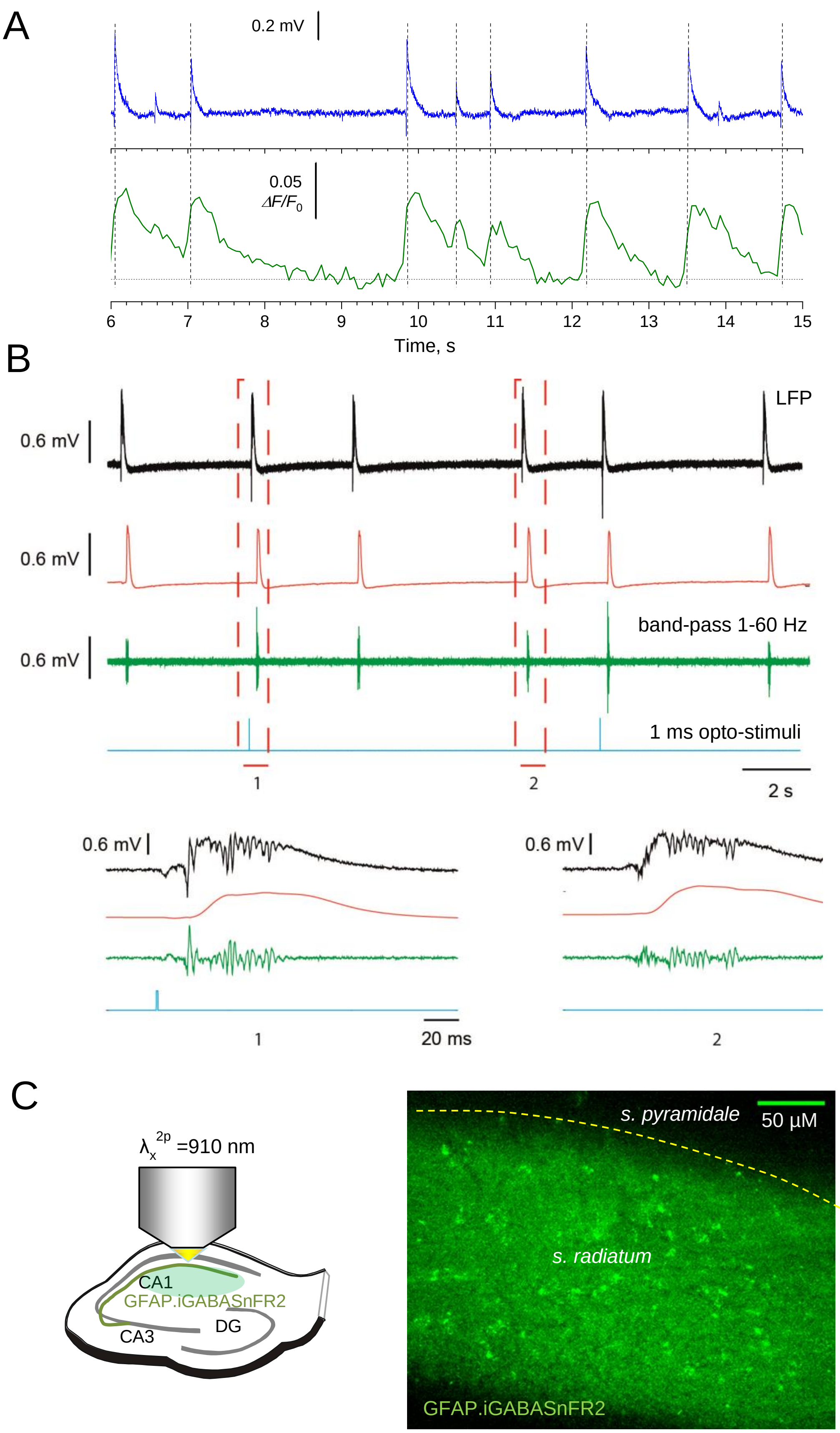

Figure S2. Monitoring interictal discharges and $[GABA]_e$, Related to Figure 2.

(A) Example of fEPSPs (top, blue) and iGABASnFR2 signal (bottom, green) recorded during interictal spikes; vertical dotted lines, onsets of interictal spikes (note that these lag behind the onset of [GABA] rises).

(B) Example to illustrate electrophysiological recordings of interictal activity, with lowpass filtered signals, showing no shift in field potential before the IIE onsets during either spontaneous or optogenetically evoked IIEs; this suggests no significant $[K^+]_{out}$ fluctuations in this context.

(C) Diagram, experimental design illustrating two-photon excitation imaging of astrocyte-expressed iGABASnFR2 in area CA1 of the acute hippocampal slice. Image, low-magnification view of the area CA1 *s. radiatum* (green fluorescence channel, ~100 µm z-stack average) showing relatively homogenous expression of iGABASnFR2; multiple astrocyte somata can be seen.

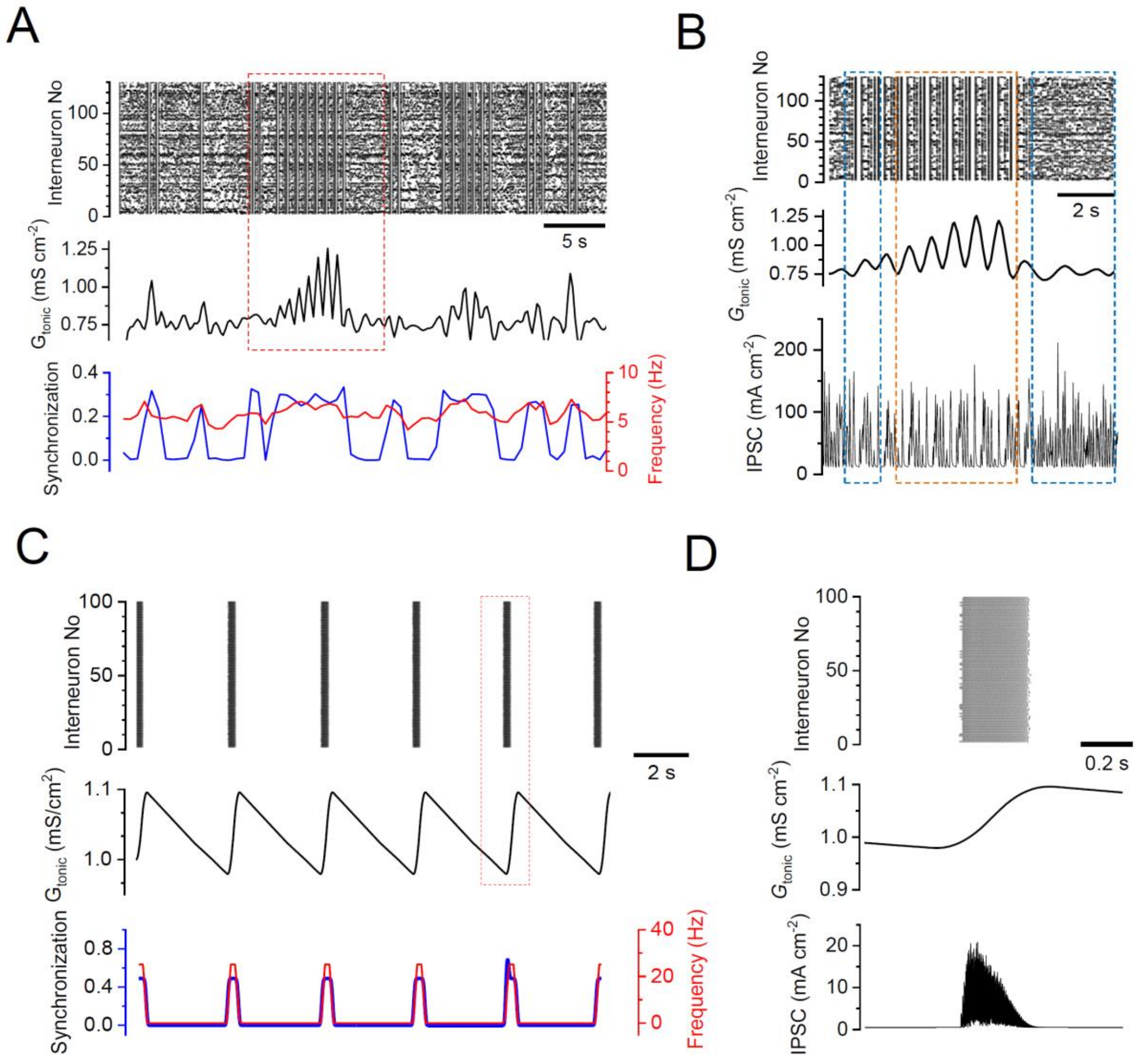

Figure S3. [GABA]$_e$-dependent G_{tonic} drives rhythmic activity of a modelled interneuron network, Related to Figure 3.

(A) Raster plot of interneuronal network spiking activity (top), G_{tonic} driven by [GABA]$_e$ (middle) calculated from integrated interneuronal discharges; and network synchrony and mean network frequency showing quasi-periodicity features (bottom). Key model parameters: cell number N = 130, intra-network peak synaptic conductance G_{ii} = 15 mS cm^{-2}; E-currents (Poisson series) with average synaptic conductance g_s=0.05 mS cm^{-2}, decay constant tau = 3 ms, and frequency f_s = 50 Hz; G_{tonic} reverse potential V_{GABA} = –55 mV, GABA release factor Af = 60 $\times 10^{-7}$ nS cm^{-2} ms^{-1}, G_{pump} =0.02 s^{-1} (see Methods for further detail).

(B) Fragment from A (red dotted rectangle) enlarged, with the IPSC series sampled from an arbitrarily selected simulated interneuron (bottom). Blue and orange rectangles, high-frequency non-synchronised, and oscillating and synchronised IPSC periods, respectively.

(C-D) Graphs as in A-B but with N = 100, g_s = 0.2 mS cm^{-2} , f_s = 50 Hz, G_{ii} = 0.02 mS cm^{-2}, V_{GABA} =–60 mV, A_f = 0.18$\times 10^{-7}$ nS cm^{-2} ms^{-1}, G_{pump} = 0.0004 s^{-1}.

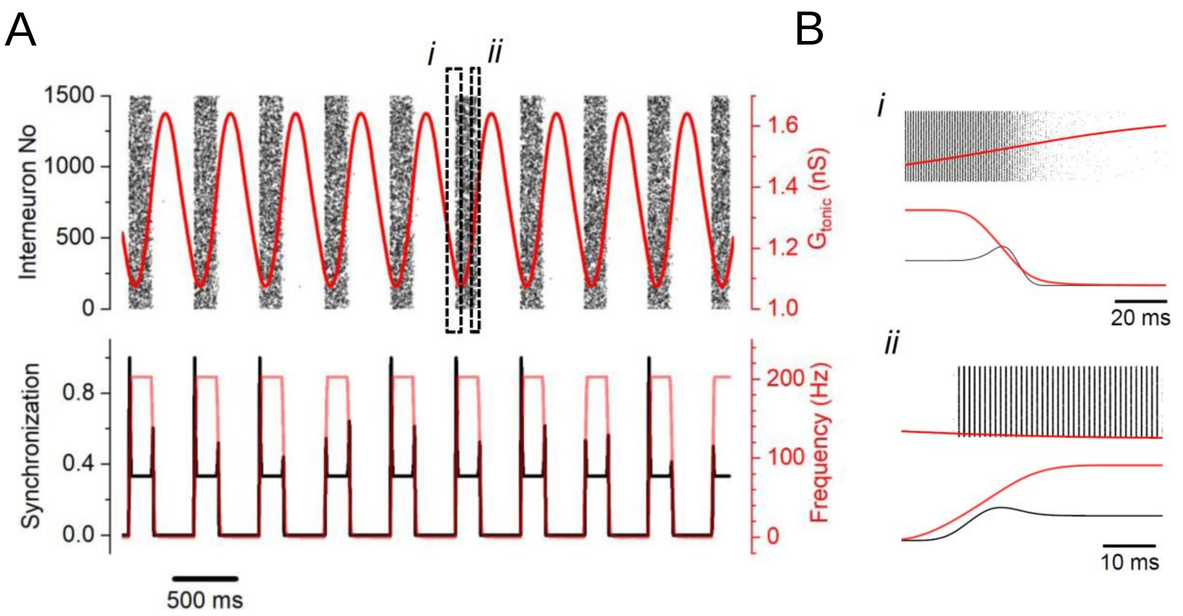

Figure S4. Increasing network size makes burst rhythms more robust, related to Figure 3.

(A) Raster plot of spiking network activity (top), network synchronisation coefficient (bottom, black), and time course of average spiking frequency (bottom, red) for the network as in Fig. 3c; key model parameters: cell number *N* = 1500, intra-network peak synaptic conductance G_{ii} = 15 nS cm^{-2}; E-currents (Poisson series) with average synaptic conductance g_s=0.05 nS, decay constant tau = 3 ms, and frequency f_s = 50 Hz; G_{tonic} reverse potential V_{GABA} = –55 mV, GABA release factor *Af* = 60 $\times 10^{-7}$ nS cm^{-2} ms^{-1}, GABA uptake rate G_{pump} =0.02 s^{-1} (see Methods for further detail).

(B) Fragments *i* and *ii* from A (dotted rectangles), as indicated, on an expanded scale; note that synchronization (black line, bottom traces) roughly follows the first time derivative of frequency (red trace).

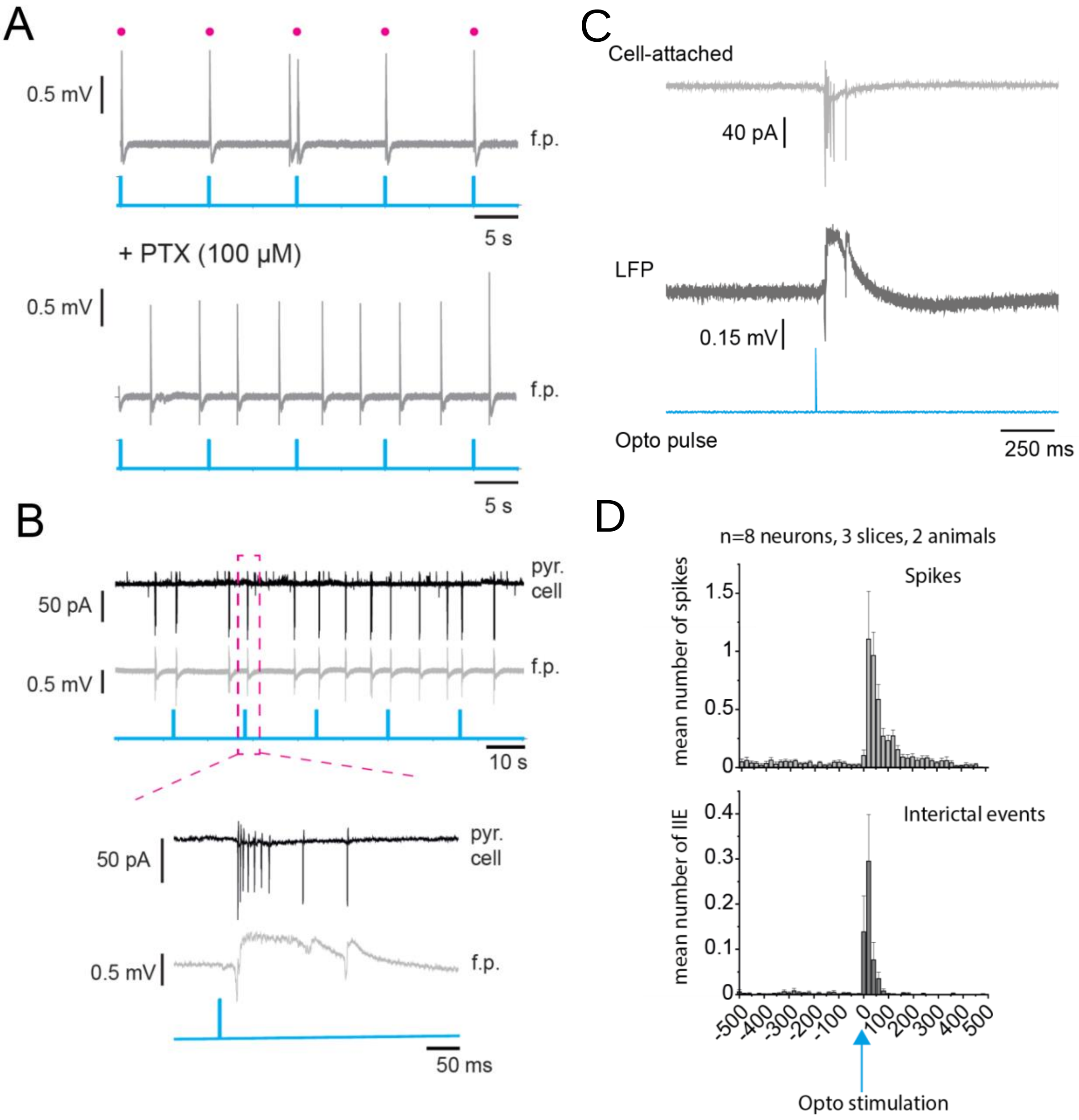

Figure S5. Light-evoked interictal discharges are GABA$_A$R-mediated engaging principal cells, Related to Figure 5.

(A) Example of local field potential (grey trace) recorded in area CA1 (5 mM K^+, 0 mM Mg^{2+}) during optogenetic activation of FS PV+ interneurons (blue). Optogenetic stimulation (1ms pulses) reliably evokes interictal events with intact GABA$_A$Rs (top panel, magenta dots), but not in the presence of picrotoxin (PTX; bottom panel).

(B) Concurrent cell-attached recording of a pyramidal neuron with the local field potential in CA1 region during interictal events.

(C) A characteristic example showing expanded traces of cell-attached current and LFP traces, as indicated.

(D) Spike number histograms; zero, onset of the optogenetic stimulus (n = 8 cells; bin, 20ms); the corrected lag time between optogenetic stimulation and IIE or spikes is 31 ± 6 ms for IIE and 46 ± 4 ms (mean ± SEM) for spikes.

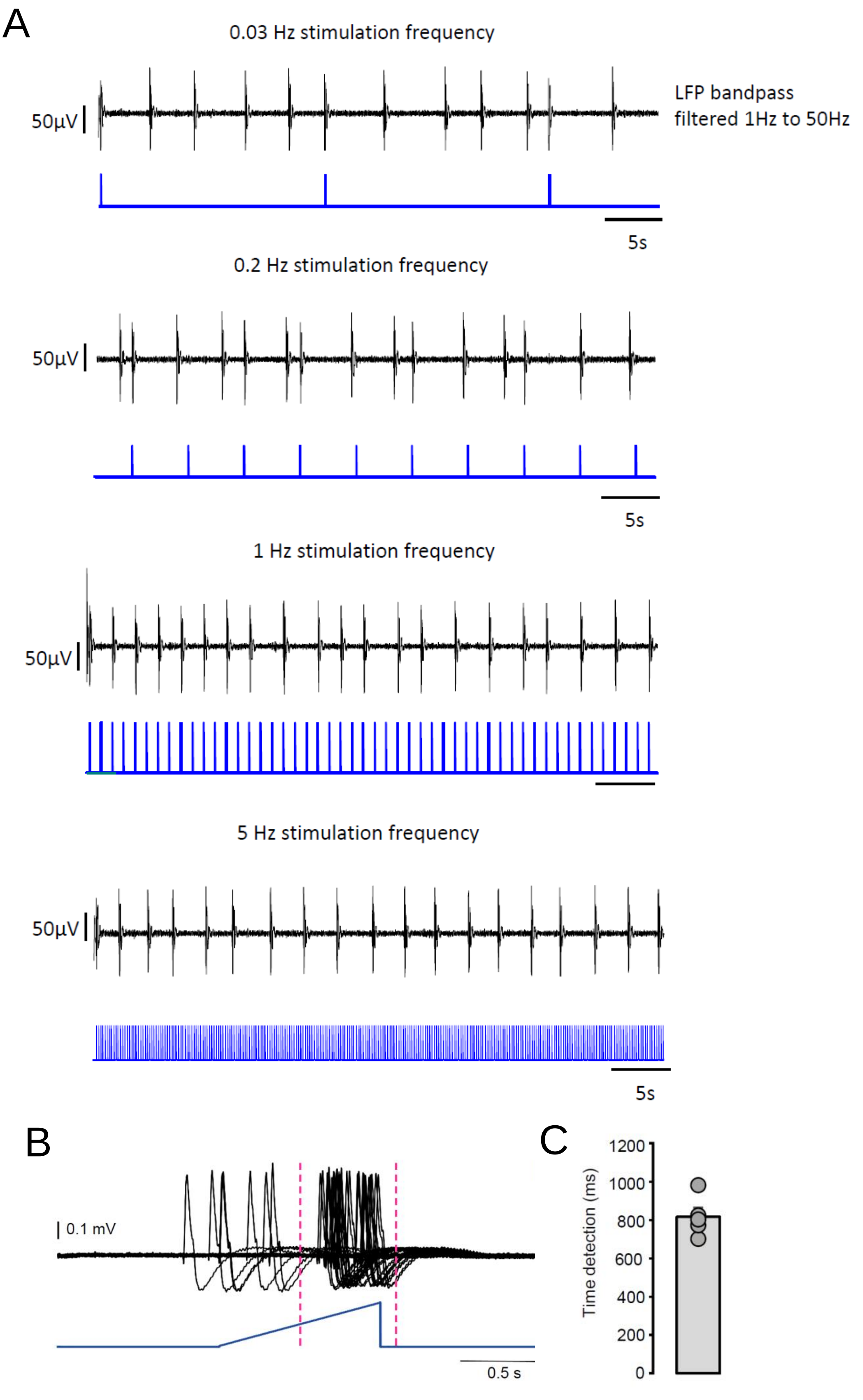

Figure S6. Recording IIEs at varied optogenetic stimulation regimes, Related to Figure 5 and Figure 6.

(A) IIEs (top traces, black; bandpass filter 1-50 Hz) recorded at varied stimulation frequencies (bottom traces, blue; individual 1 ms pulses at 470 nm wavelength).

(B) Example, superimposition of the ramped light stimulus and interictal discharges; dotted lines, 500 ms after the ramp onset, and 100 ms after the ramp endpoint.

(C) Average timepoint of the interictal spike after the ramp onset (bar, mean ± SEM; dots, individual experiments; n = 5 slices in 4 animals).

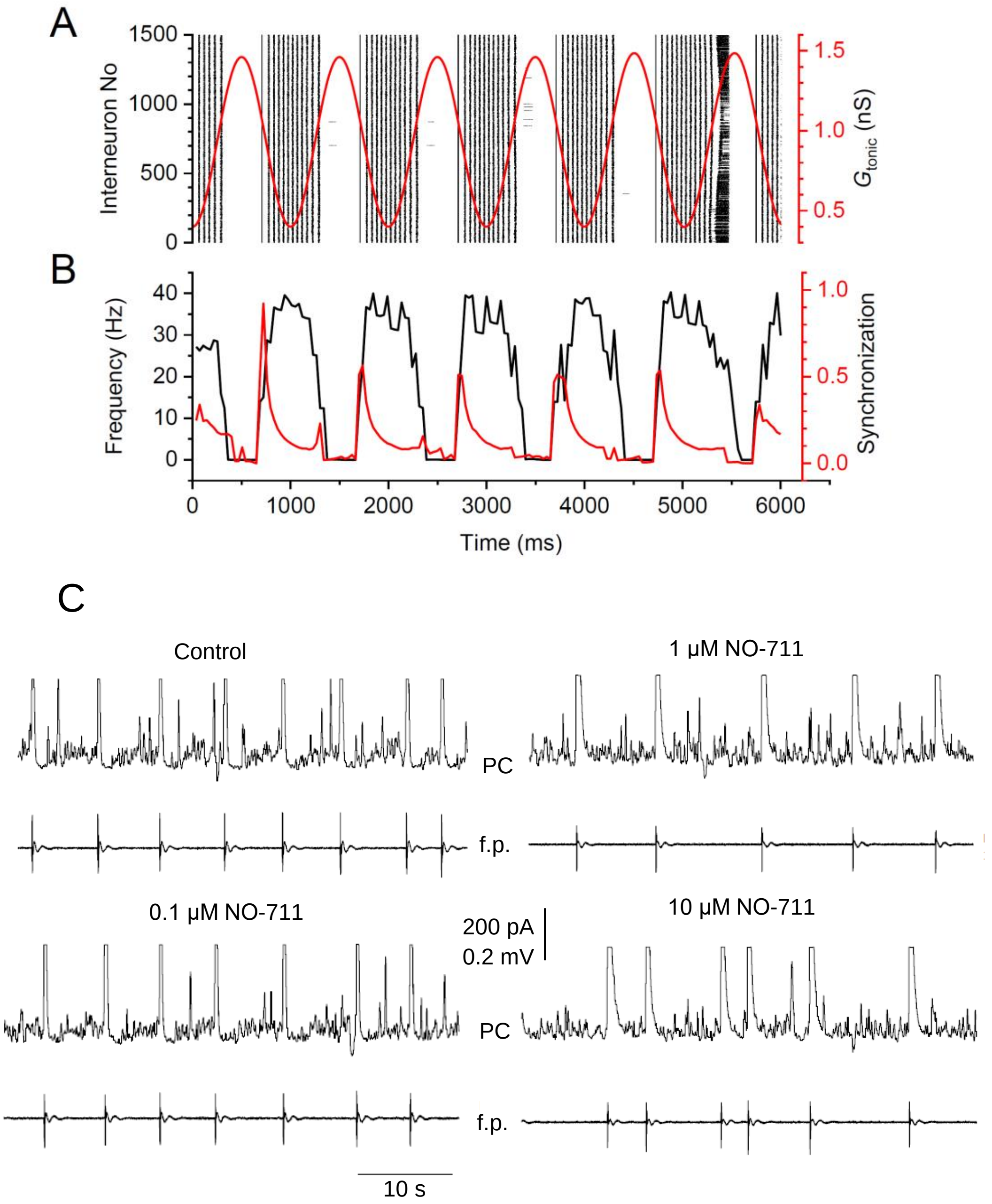

Figure S7. Imposing cycling changes in G_{tonic} or partial blockade of GABA uptake paces rhythmic network activity, Related to Figure 6 and Figure 7.

(A) Raster plot of cell spiking (black) under 1 Hz sin wave of G_{tonic} (red), as indicated.

(B) Average firing frequency and synchronization parameter for network activity shown in A. Key model parameters: cell number N = 1500, intra-network peak synaptic conductance G_{ii} = 0.096 nS cm^{-2}; E-currents (Poisson series) with average synaptic conductance g_s = 0.05 nS, decay constant tau = 3 ms, and frequency f_s = 20 Hz; G_{tonic} reverse potential V_{GABA} = –53 mV, GABA release factor A_f = 10^{-8} nS cm^{-2} ms^{-1}, G_{pump} =0.004 s^{-1} (see Methods for further detail).

(C) Example, simultaneous recording of CA1 pyramidal cell GABAergic currents (top traces; voltage clamp at +10 mV; 30 Hz lowpass filter) and local field potentials (bottom traces, 1-30 Hz bandpass filter), at varied concentrations of NO-711, as indicated; scales as indicated apply throughout the panels.
